# Phospho-tyrosine dependent protein–protein interaction network

**DOI:** 10.15252/msb.20145968

**Published:** 2015-03-26

**Authors:** Arndt Grossmann, Nouhad Benlasfer, Petra Birth, Anna Hegele, Franziska Wachsmuth, Luise Apelt, Ulrich Stelzl

**Affiliations:** Otto-Warburg Laboratory, Max-Planck Institute for Molecular Genetics (MPIMG)Berlin, Germany

**Keywords:** cancer signaling, network biology, post-translational protein modification, yeast two-hybrid

## Abstract

Post-translational protein modifications, such as tyrosine phosphorylation, regulate protein–protein interactions (PPIs) critical for signal processing and cellular phenotypes. We extended an established yeast two-hybrid system employing human protein kinases for the analyses of phospho-tyrosine (pY)-dependent PPIs in a direct experimental, large-scale approach. We identified 292 mostly novel pY-dependent PPIs which showed high specificity with respect to kinases and interacting proteins and validated a large fraction in co-immunoprecipitation experiments from mammalian cells. About one-sixth of the interactions are mediated by known linear sequence binding motifs while the majority of pY-PPIs are mediated by other linear epitopes or governed by alternative recognition modes. Network analysis revealed that pY-mediated recognition events are tied to a highly connected protein module dedicated to signaling and cell growth pathways related to cancer. Using binding assays, protein complementation and phenotypic readouts to characterize the pY-dependent interactions of TSPAN2 (tetraspanin 2) and GRB2 or PIK3R3 (p55γ), we exemplarily provide evidence that the two pY-dependent PPIs dictate cellular cancer phenotypes.

See also: D Ochoa & P Beltrao (March 2015)

## Introduction

Systematic mapping of human protein interactions is indispensable for deciphering the molecular networks that underlie cellular phenotypes (Vidal *et al*, 2011; Woodsmith & Stelzl, 2014). Networks of protein–protein interactions (PPIs) mediate cellular signal transduction with time-dependent contributions of hundreds of proteins to the information flow (Friedman & Perrimon, 2007; Vinayagam *et al*, 2011). In addition, interactome networks are conditional with respect to the signaling status of the cell. The cellular response to signals is frequently initiated by the reversible covalent post-translational modification (PTM), especially phosphorylation of proteins already present. Modifications are recognized by interacting proteins effectively rewiring cellular networks by switching PPIs on or off. Thus, signals are propagated through PTM-mediated, that is, conditional, PPIs in the cell.

A variety of protein families containing folded domains capable of specifically recognizing PTMs have been characterized (Seet *et al*, 2006). To recognize phospho-tyrosine (pY)-mediated signals, proteins largely rely on two well-defined domain modules, the Src homology 2 (SH2) and the phospho-tyrosine-binding (PTB) domains. They are contained in more than 100 human proteins which play a central role in tyrosine kinase signaling pathways critical for cellular growth (Schlessinger & Lemmon, 2003; Liu *et al*, 2006). SH2 domains canonically recognize protein tyrosine phosphorylation. There are several subgroups of PTB domains, some of which function in phospho-tyrosine recognition, while others, for example, bind phospholipids. Linear peptide consensus motifs around phosphorylated tyrosine residues have been deduced from peptide-based *in vitro* assays examining the binding capacities of isolated SH2 or PTB domains (Songyang & Cantley, 2004; Huang *et al*, 2008; Miller *et al*, 2008; Koytiger *et al*, 2013; Tinti *et al*, 2013). Though amino acid residues surrounding the phospho-tyrosine clearly contribute to recognition specificity, their predictive value on a proteome scale for whether a tyrosine phosphorylation promotes an SH2- or PTB-mediated protein interaction is relatively small. *In vivo*, interaction and complex formation with other human proteins, compartmentalization and additional PTMs add to the specificity of pY recognition.

More than thirty thousand *in vivo* tyrosine phosphorylation sites have been identified in human by mass spectrometry-based proteome-wide mapping (Tan *et al*, 2009; Olsen *et al*, 2010; Hornbeck *et al*, 2012; Woodsmith *et al*, 2013). The majority of these sites are not contained in known linear recognition motifs. Thus, the attribution of kinases and pY-binding proteins to these modifications is a daunting task. Kinase specificity has been approached through a network-based computational approach (Linding *et al*, 2007) demonstrating that context contributes 60–80% to substrate specificity. Similarly, specific recognition of pY by binding proteins may also depend on various parameters, for example, the domain composition and 3D-fold of the full-length proteins, which are mostly not accounted for in current approaches for the detection of pY-dependent interactions.

Using Co-IP-MS approaches that concomitantly and quantitatively measure binding and phosphorylation, phosphorylation-regulated signaling complexes can be inferred dynamically (Zheng *et al*, 2013). However, powerful approaches that directly measure modification-dependent binary interactions are lacking. We extend our well-established yeast two-hybrid system employing active human tyrosine kinases to search for pY-dependent protein interactions (pY-Y2H) and detected 292 mostly novel pY-dependent PPIs through a large-scale screening exercise. Our approach is examining phospho-dependent binary interactions of full-length human proteins at cellular concentrations in the lower range in a crowded cellular environment. With this approach, we addressed a critical gap in pY-dependent PPI knowledge providing a unique set of conditional human protein–protein interactions.

## Results

### Screening for pY-dependent interactions with a modified Y2H approach

**Figure 1:**
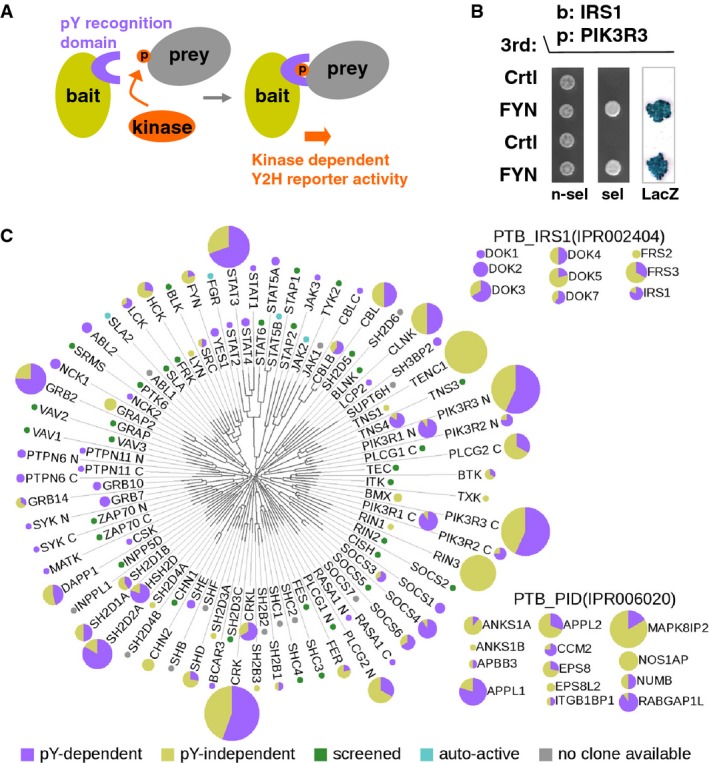
Large-scale pY-Y2H screening for pY-dependent human protein interactions

In a yeast two-hybrid protein interaction analysis, reporter activity depends on the interaction of two hybrid proteins expressed as transcription factor DNA binding and activation domain fusion proteins from the bait or prey plasmid, respectively. Typically, such a system is insensitive to interactions that require post-translational protein modification not sustained by the yeast itself (Braun *et al*, 2009; Venkatesan *et al*, 2009). We introduced a third plasmid expressing human non-receptor tyrosine kinases to enable detection of phospho-tyrosine-dependent interactions in yeast (Fig1A). In agreement with the known pY dependency, the interacting proteins PIK3R3 and IRS1 (Myers *et al*, 1992; Mothe *et al*, 1997) support yeast growth on selective medium and produce a β-galactosidase staining in the presence of an active FYN kinase construct, but not in its absence (Fig1B). Only very little kinase activity, below the detection limit of the standard pan-pY 4G10 antibody, is required to promote this interaction demonstrating the high sensitivity of the pY-Y2H system with respect to kinase activity (Supplementary Fig S1). Because of its modular nature, the pY-Y2H setup is fully compatible with our established resources and screening technology (Stelzl *et al*, 2005; Vinayagam *et al*, 2011; Weimann *et al*, 2013), allowing the screening for pY-dependent PPIs on a proteome-wide scale. Several phospho-tyrosine-dependent interactions have been detected in targeted studies by means of Y2H approaches before (Supplementary Table S1); however, no systematic screening study has been reported.

Schematic of the pY-Y2H assay. An active kinase was introduced to phosphorylate prey (or bait) proteins, thus allowing pY-dependent interactions. Our screen was performed with bait proteins containing at least one pY-recognition, that is, SH2 or PTB, domain (pY readers) on the assumption that the bait will bind to phosphorylated prey. Kinase substrates are thus identified through interaction with pY-recognizing proteins.

Proof-of-principle experiment showing the Y2H phenotypes of the kinase-dependent interaction between IRS1 and PIK3R3 (p55γ). The assay was performed in duplicate: Growth on non-selective media (n-sel), on selective media (sel) without histidine and uracil indicative of an interaction and the result of a β-galactosidase assay (LacZ) are shown in the absence and presence of active FYN kinase.

Overview of the Y2H screen (see also Supplementary Fig S2). The SH2-domain-containing human proteins are presented around an un-rooted tree indicating the sequence similarity between the SH2 domains (Liu *et al*, 2006). Color-coded pie charts show the interaction result for every protein (purple: pY-dependent, yellow: pY-independent PPIs), the sizes of the charts indicate the relative number of interactions found. The results obtained with the IRS1 and PID families of PTB domain-containing proteins are presented aside as insets.

In order to systematically screen for pY-dependent PPIs, we paired bait proteins containing phospho-tyrosine-recognition domains (pY readers, Lim & Pawson, 2010) with non-receptor tyrosine kinases and tested a human proteome-scale prey matrix containing ∽17,000 ORFs for interaction. The pY-PPI screen was based on the assumption that the kinase will phosphorylate a prey protein from our matrix and the pY-reader protein, the bait, will bind to the phosphorylated prey. Thus, knowledge of neither kinase–substrate relationships nor phosphorylation site is prerequisite (Fig1A). The baits were pY readers with SH2 domains and proteins containing PTB domains belonging to subgroups clearly implicated in pY recognition. All UniProt entries with the InterPro identifiers IPR000980 (SH2), IPR002404 (PTB_IRS1) or IPR006020 (PTB_PID) were mapped to 149 NCBI Entrez GeneIDs, and 188 ORFs for 126 genes (85%) were generated (Supplementary Table S2). We selected nine tyrosine kinases (TKs), each as a representative of a different non-receptor tyrosine kinase family (Blume-Jensen & Hunter, 2001), and introduced them on the third plasmid for pY-Y2H screening: FYN (Src family), ABL2 (Abl family), TNK1 (Ack family), FRK (Frk family), FES (Fes family), PTK2 (Fak family), SYK (Syk family), BMX (Tec family) and JAK2 (Jak family). Yeast MATa strains were individually co-transformed with the SH2/PTB pY-reader bait and one TK each and screened four times in pools of eight baits against the prey matrix (24 strains per pY reader per replica screen; for details, see Materials and Methods). To increase coverage, additional ORFs representing pY readers, for which we did not obtain interactions in the first screen, were screened four times with three kinases (FYN, ABL2, TNK1). In total, these screens examined ∽2 million possible unique pairwise interactions (see flow chart Supplementary Fig S2). In the retest, performed for 1,223 primary hits that have shown up at least twice, kinase dependency was assayed individually with all TKs as well as empty control vectors. Both kinase-dependent and kinase-independent interactions are found and distinguished in such an approach (Supplementary Fig S3). We also tested a selected, representative subset of 37 identified interacting pairs with kinase-deficient version of the 9 kinases, rendered inactive by an arginine to methionine mutation in the ATP binding site. None of the kinase-dependent interactions gave positive reporter readouts with the kinase-inactive versions supporting the notion that the identified kinase-dependent interactions are likely phosphorylation dependent in the Y2H assay system. In this assay, we additionally examined these interactions with a comprehensive set of 31 non-receptor tyrosine kinases. We observed high interaction specificity between putative substrates and pY readers, modulated by partially overlapping groups of kinases (Supplementary Fig S4). In summary, we report a large data resource comprising 336 independent and 292 phosphorylation-dependent protein–protein interactions, the latter involving 52 SH2-containing, 19 PTB domain-containing proteins (Fig1C) and 157 putative tyrosine-phosphorylated proteins identified as prey (Supplementary Tables S3 and S4).

### A network of pY-dependent protein interactions

**Figure 2:**
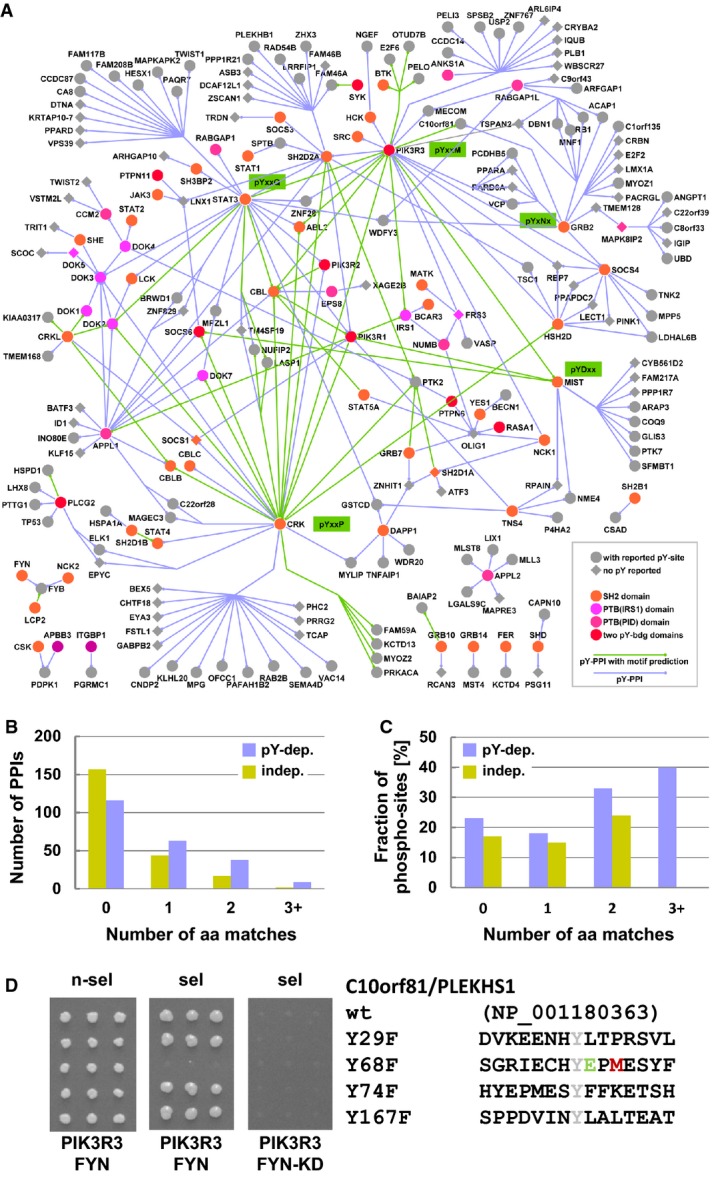
Phospho-tyrosine-dependent protein interaction network

The resulting collated network is unexpectedly dense and highly connected. Taken alone, the network of pY-dependent interactions forms one giant component comprising all but 18 interactions (Fig2A). The number of pY reader–pY reader interactions is much higher than expected. Statistically, little more than 8 interactions are expected in the complete set by chance and less than three in the phospho-tyrosine-dependent set. We found 51 (*P* = 4.41 × 10^−58^) and 30 (*P* = 6.31 × 10^−38^) pY reader–pY reader interactions, respectively. The interacting phospho-proteins identified as prey were highly enriched for GO molecular function and biological process terms related to signaling. For molecular function, the most highly enriched GO terms were “SH3/SH2 adaptor activity”, “protein phosphorylated amino acid binding” and “kinase binding” (Supplementary Fig S5A). For biological process, the most enriched and most common term was “intracellular signal transduction” (Supplementary Fig S5B). The most enriched pathways (Kamburov *et al*, 2013) were cytokine and growth factor receptor signaling pathways, such as BCR, Kit Receptor, ErbB, VEGFR, Tie2, insulin and FGF signaling pathways (Supplementary Fig S5C), as is expected for a network related to phospho-tyrosine signaling. We also performed gene neighborhood enrichment analysis in an integrated human PPI network from ConsensusPathDB (Kamburov *et al*, 2013). The most enriched gene neighborhoods were centered on well-known signaling genes including Cbl, Gab or Sos, non-receptor tyrosine kinases and receptor tyrosine kinases such as ERBB2, KIT or INSR (Supplementary Fig S5D). Notably, receptor tyrosine kinases were not part of the pY reader or kinase set in the screen. Examples for pY-dependent connections to other pathways can be found at the network periphery, for example, in SH2D2A pY-dependent interactions with RAD54B, likely involved in DNA damage response and the cullin-associated WD domain-containing protein DCAF12L1 or the pY-dependent interactions of the AAA+ ATPase VCP (p97), a key player in several cellular processes. Finally, the interacting proteins are highly enriched in the current cancer gene census set (all interacting prey: *P* = 2.38 × 10^−5^, pY-interacting prey 2.83 × 10^−4^, hypergeometric test, Supplementary Table S5) (Futreal *et al*, 2004). This analysis confirmed that phospho-tyrosine-triggered interactions play roles in cellular signaling and growth response pathways related to cancer (Lim & Pawson, 2010).

Static network representation of 292 pY-dependent protein interactions. Proteins (Entrez Gene Symbols) containing a pY-reader domain are colored in orange (SH2) or magenta (PTB) or red (two domains); round nodes indicate proteins reported to be phosphorylated on one or more tyrosines in human cells (Hornbeck *et al*, 2012). Green lines indicate interactions that are predicted to be mediated by a known linear SH2-domain binding motif sequence surrounding a reported phospho-Y site; purple lines indicate interactions that are mediated by unknown linear motifs or are governed by alternative recognition modes. The scale-free network showed a degree distribution with a power law exponent of −1.41 and an average number of 2.75 interactions per protein indicative for high connectivity. Like with any static network representation, this network graph does not capture any likely situation in the cell (Stelzl, 2013). This is even more so in this pY-dependent network as all links displayed are conditional with respect to kinase activity. Interactions will depend on the signaling status of the cell and thus be relevant at very different points in time and location.

Motifs in interacting proteins. Distribution of consensus motif-matching amino acid residues for all pairs of domain-containing proteins and interaction partners in the phosphorylation-dependent and phosphorylation-independent set in purple and yellow, respectively. Number of PPIs is shown according to the number of amino acid (aa) matches in the motif. The distribution corresponding to the phosphorylation-dependent interaction set is shifted to the right. The probability that both distributions are samples drawn from the same underlying distribution is *P *= 3.957 × 10^−6^ (Wilcoxon rank-sum test).

Known phospho-tyrosines in motifs. For each interaction, all tyrosine residues in the putative phosphorylated protein were categorized according to the number of surrounding residues matching the respective consensus motif. Each interaction was then categorized by the highest number of residues matching the respective consensus motif. Percentage of known phospho-tyrosine sites according to the number of matching aa residues in known SH2 binding motifs in the two sets is displayed for the best matching motifs for each interaction. The level for non-matching motifs is higher for pY-dependent partners, around 23 and 17% for pY-dependent and pY-independent PPIs, respectively. For motifs matching in two or more than two positions, the percentages were higher, a trend observed for phosphorylation-dependent and phosphorylation-independent interaction. For three matching positions, only two 3+ matches were found in the pY-independent interactions; however, neither was a known phosphotyrosine site.

Example for linear motif recognition of the novel interaction pair PIK3R3-C10orf81/PLEKHS1. Y68F mutation in C10orf81/PLEKHS1 (NP_001180363) within a linear sequence matching a known PIK3R3 motif abolishes kinase-dependent binding in the pY-Y2H system (FYN). Mutation of the other tyrosine residues to phenylalanine in C10orf81/PLEKHS1 does not affect growth of yeast colonies (triplicate experiment). A kinase-inactive FYN mutant (Fyn-KD, K299M) does not promote the interaction. n-sel: non-selective media (SD3), sel: selective media (SD5).

### Linear motif recognition by SH2-containing pY-reader proteins

Using peptide-based approaches, linear peptide sequences surrounding the pY have been shown to contribute to the recognition specificity of binding domains such as the SH2 domain (Huang *et al*, 2008; Miller *et al*, 2008; Tinti *et al*, 2013). Even though the information content is low, these motif sequences can be searched for among the interactors for each SH2-domain-containing protein in our data set. The tyrosine motif with the highest number of amino acid residue matches to the respective binding motif in the prey sequence is the most probable binding site. Comparing the pY-dependent with the pY-independent data set, the distribution of the highest matching number of amino acids per prey is clearly shifted to higher values (Wilcoxon rank-sum test *P* = 3.975 × 10^−6^, Fig2B). This is also reflected in the number of individual matches for each domain, which is consistently higher in the pY-dependent than in the set of pY-independent interactors (Supplementary Table S6). Though the exact interaction site is not known, we assumed that, on average, tyrosine residues matching the respective binding motif are more likely to be recognized and therefore will be phosphorylated. The fraction of known phospho-sites is indeed higher in the set of pY-dependent interacting proteins than in the pY-independent data. The difference is seen for proteins that do not contain putative binding motifs and is even more pronounced for tyrosines within known SH2/PTB domain recognition motifs (Fig2C). Using motif information in combination with known phospho-sites, we can predict pY-binding sites for 50 interactions in our data (Fig2A, green PPIs). Exemplarily, we tested the interaction between PIK3R3 (p55γ regulatory subunit of PI3 kinase) and C10orf81 in the pY-Y2H assay. C10orf81, a previously uncharacterized protein also named PLEKHS1, is conserved in vertebrates with no related sequences in the human genome. C10orf81/PLEKHS1 has four tyrosine sites, residues 29, 68, 74 and 167. In agreement with an established PIK3R3 recognition motif […pYExM…] (Songyang *et al*, 1993), mutation of Y68 to F, a reported *in vivo* phospho-site (Hornbeck *et al*, 2012), abolished PIK3R3 binding (Fig2D). Selective inactivation of the N-terminal or the C-terminal SH2 domain of PIK3R3 demonstrated that the C-terminal SH2 domain specifically recognizes pY68 of C10orf81/PLEKHS1 (Supplementary Fig S6). In conclusion, a fraction (∽1/6) of the novel pY-dependent interaction can be explained by canonical linear peptide motif recognition of *in vivo* phospho-tyrosine sites, in agreement with the current view on pY recognition (Liu *et al*, 2012).

### Validation of pY-dependent PPIs through literature comparison

Our experimental approach did not assay short peptides but folded full-length proteins in a cellular environment. Therefore, as expected, many interactions do not involve known linear binding motifs suggesting that the pY-Y2H approach also reveals pY-dependent interactions that are mediated by unknown linear epitopes or are governed by alternative recognition modes (Bradshaw & Waksman, 2002; Higo *et al*, 2013). It has been suggested for kinase substrate recognition that only ∽20% of the observed specificity can be explained by the amino acids directly next to the phospho-site in linear sequence (Linding *et al*, 2007) and similar principles may hold true for pY-binding events (Miller *et al*, 2008). Therefore, we independently validated our data (i) by comparison with the literature knowledge and (ii) through co-immunoprecipitation analysis in mammalian cells. The comparison to the literature allows estimating the novelty of the interaction set and the number of interactions in the interaction space awaiting discovery. The protein interaction meta-database ConsensusPathDB (Kamburov *et al*, 2013) was used to manually define 147 literature-curated interactions in the pY-reader–pY-reader search space, 15 of which overlapped with our data set (Supplementary Table S7). Therefore, ∽81% of our data were novel and these numbers suggest that ∽73% of the interactions remain to be discovered in the pY reader–pY reader search space. This space is much better covered by literature-curated interactions and thus better suited for comparison; however, the respective analyses for the whole data set showed that ∽5.6% of the PPIs were reported previously (Supplementary Fig S7).

### Validation of pY-dependent PPIs in co-IP assays from mammalian cells

**Figure 3:**
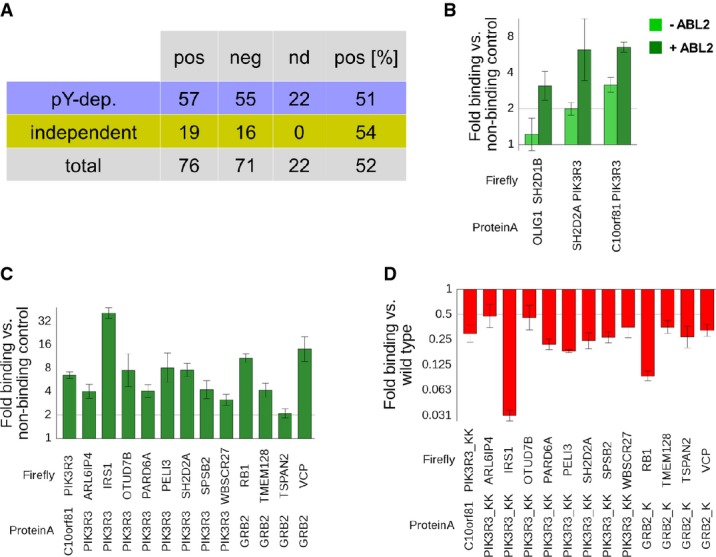
Validation of pY-PPIs through luciferase-based co-IP assays

To validate the precision of the interaction data obtained in this study, a set of 169 interactions was assayed in a medium throughput luminescence-based co-immunoprecipitation (co-IP) assay (Hegele *et al*, 2012; Weimann *et al*, 2013). A total of 147 interacting pairs produced valid results, that is, both proteins were expressed in HEK293 cells at levels that allow detection by Western blotting, and a total of 76 interactions were successfully validated. The co-IP signal for these interacting pairs exceeded background binding by a factor of at least two and at least two standard deviations (Fig3A and Supplementary Table S8). Since most interaction assays are orthogonal, each detecting its own subset of true interactions, validation of data sets means comparing validation rates rather than discarding pairs that do not bind in the co-IP assay (Braun *et al*, 2009; Venkatesan *et al*, 2009). The validation rate of ∽50% (Fig3A) is similar for phospho-tyrosine-dependent and phospho-tyrosine-independent interactions. It is lower than what was observed for more stable PPIs such as spliceosomal interactions (Hegele *et al*, 2012). However, it compares well to validation rates reported for other representative sets of PPIs with this co-IP assay (Braun *et al*, 2009; Weimann *et al*, 2013).

Co-IP results: 187 PPIs were tested in co-IP assays with a success rate of ˜50%. The background rate in this assay was 20–25% (Hegele *et al*, 2012; Weimann *et al*, 2013).

Co-IP results for the three pY-dependent pairs that showed significantly higher binding in the co-IP assay upon induction of ABL2 (dark green) as compared to non-induced cells (light green). *y*-axis displays fold binding in comparison with a non-interacting protein tested with the same firefly-tagged protein (normalized to 1). Error bars show the standard deviation from an experiment performed with triplicate transfections.

Selection of PIK3R3 and GRB2 co-IP results from HEK293 cells. Co-IPs were performed in HEK293 with induction of stably transfected ABL2 kinase and tyrosine phosphatase inhibition throughout the cell lysis. Error bars show the standard deviation from an experiment performed with triplicate transfections.

The same pY-dependent protein pairs as in (C), but tested with SH2-domain mutant versions of GRB2 (R86K) and PIK3R3 (R90K+R383K). The amino acid exchange of the conserved arginine in the SH2 domain is known to reduce phosphorylation-dependent binding. Fold binding is normalized to wild-type GRB2 or PIK3R3 binding (1). Error bars show the standard deviation from an experiment performed with triplicate transfections.

These results show that a large fraction of pY-dependent interactions were recapitulated by co-IP in the standard cell line HEK293. However, only 3 of the tested interactions (PIK3R3-SH2D2A, SH2D1B-OLIG1, PIK3R3-C10orf81/PLEKHS1) showed a strong (> 2-fold) increase in binding upon induction of a stably expressed tyrosine kinase such as ABL2 (Fig3B). We hypothesized that the high success rate in the co-IP approach for pY-dependent interactions (Fig3C) is due to high intrinsic tyrosine kinase activity of this and other fast-growing cell lines (Yaoi *et al*, 2006). To test phosphorylation requirement in mammalian cells in spite of that, we assayed PIK3R3 and GRB2 interactions using point mutations of a conserved arginine in the SH2 domains of PIK3R3 and GRB2 that abolish pY binding (Mayer *et al*, 1992; Bisson *et al*, 2011) (Supplementary Table S9). Indeed, several pY-dependent interactions showed strongly reduced binding when tested with the full-length GRB2 (R86K) mutant (Fig3D). PIK3R3 contains two very similar SH2 domains at the N- and C-termini, respectively. In the majority of the cases, the C-terminal SH2-domain mutation (R383K) had a stronger diminishing effect on binding; however, when both SH2 domains were mutated simultaneously, pY-dependent binding was typically decreased most strongly (Fig3D and Supplementary Fig S8). These experiments orthogonally confirm the phosphorylation requirement for a fraction of the interactions identified in the pY-Y2H system suggesting that kinase activities in proliferating cells may often mask the contribution of phosphorylation to interactions involving SH2-domain-containing proteins (see also Supplementary Table S7, where we liberally inferred phosphorylation dependency for 70 of the 167 literature interactions). This information is, however, important as the phosphorylation status of interacting proteins is highly variable with respect to the signaling status of the cell (Olsen *et al*, 2010) and will lead to substantial rewiring of protein interactions dictating cellular phenotypes.

### Phospho-tyrosine-dependent interactions with TSPAN2

We next provide a specific example for an edge-driven cellular phenotype, that is, when a conditional interaction of a protein is associated with a distinct phenotypic outcome. We investigated the pY-dependent interactions of TSPAN2 with two pY-reader proteins, GRB2 and PIK3R3, in parallel. TSPAN2 is one of the 33 members of the tetraspanin protein family found on the plasma membrane and membranes of intracellular organelles in nearly all cell and tissue types. Tetraspanins are known to form microdomains and to bind integrins, receptor tyrosine kinases (RTKs) and intracellular signaling molecules influencing signaling outcome in terms of cell adhesion, migration, invasion or cell–cell fusion properties associated with various disease phenotypes, in particular with cancer (Hemler, 2014). GRB2 is an adaptor protein with a central SH2 domain, flanked by two SH3 domains, and is an important hub in growth factor signaling (Bisson *et al*, 2011). PIK3R3 (p55γ) is one of five regulatory phosphoinositide-3-kinase subunits active in a variety of growth-promoting responses implicated in human cancer (Wong *et al*, 2010). The two proteins have occasionally been found in signaling complexes together (Bisson *et al*, 2011; Higo *et al*, 2013).

**Figure 4:**
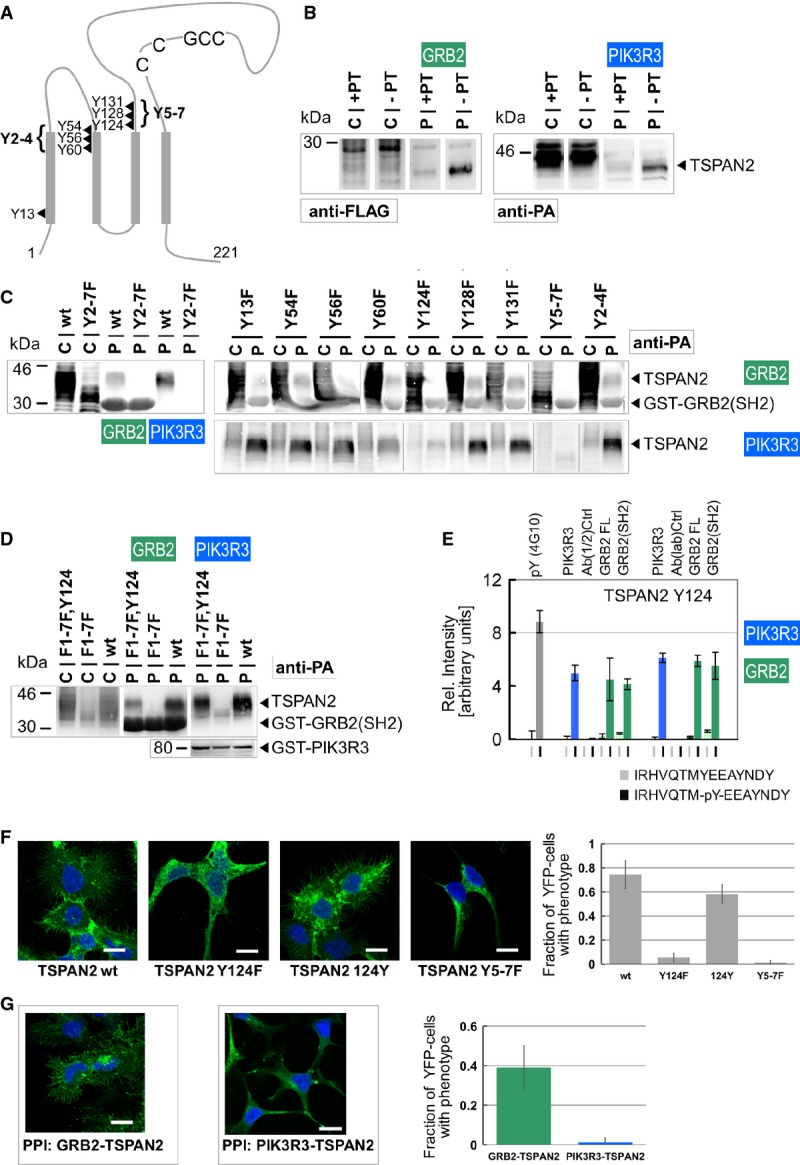
Phosphorylation-dependent interactions of TSPAN2 with GRB2 and PIK3R3

The TSPAN2 sequence comprises 7 tyrosines, a solitary N-terminal one and two patches of three tyrosines in the primary sequence (amino acid positions 13, 54, 56, 60, 124, 128 and 131; Fig4A). TSPAN2 was co-immunoprecipitated with GRB2 requiring an SH2 domain capable of pY binding (Fig3). In GST pull-down experiments, phosphatase treatment of the cell lysate strongly reduced TSPAN2 binding to GRB2 and PIK3R3 (Fig4B). We mutated all 7 tyrosine residues in TSPAN2 individually and those in proximity in batches. Both GRB2 and PIK3R3 bound wild-type TSPAN2 but not a TSPAN2 version that has all tyrosine residues mutated to phenylalanine. The expression levels of the individual mutant version of this membrane protein vary somewhat. Nevertheless, all TSPAN2 versions carrying the Y124F mutation abolished GRB2 binding and strongly affected PIK3R3 binding, suggesting that Y124 phosphorylation is critical for both interactions (Fig4C). Finally, restoring Y124 in an otherwise all Y to F mutant TSPAN2 version (TSPAN2-124Y) restored binding to GRB2 and PIK3R3 in the pull-down assay (Fig4D). We additionally probed a large peptide array including each 15 amino acid TSPAN2 fragment centered on a tyrosine for binding of *E. coli*-purified GRB2 and PIK3R3. We observe selective binding of the GRB2-SH2 domain, full-length GRB2 and PIK3R3 to the phosphorylated version of the Y124 peptide only (Fig4E and Supplementary Fig S9). Together, we conclude that TSPAN2 phospho-Y124 is necessary and sufficient for interaction with both GRB2 and PIK3R3.

Secondary structure sketch (Lazo, 2007) of 221 aa TSPAN2 (NP_005716) indicating seven tyrosines by position.

GST pull-down experiment in the presence and absence of phosphatase (+/− PT) with immobilized GRB2(SH2) or PIK3R3(FL) and FLAG-tagged or protein A (PA)-tagged TSPAN2, respectively. Lanes labeled with “C” indicate whole-cell lysate, “P” indicates the sample from the pull-down. The bound TSPAN2 protein was treated with deglycosylation mix to obtain more focused bands on the blot in this experiment (P), which consequently runs at lower size than the input (C) on the SDS–PAGE.

GST pull-down experiments assaying PA-TSPAN2 point mutations for interaction with GRB2(SH2) or PIK3R3. All constructs that contain a Y124F mutation show strongly reduced binding.

Reintroducing Y124 to PA-TSPAN2 in GST pull-down experiments. TSPAN2 that has a single tyrosine at position 124 binds to GRB2(SH2) and PIK3R3.

Peptide binding results from a peptide microarray. Phosphorylated 15-mer surrounding Y124 did react with the pY-specific antibody 4G10. Also, GST-tagged, purified PIK3R3, GRB-FL and GRB2(SH2) domain bound the phosphorylated peptide, when detected either with a combination of primary and secondary antibodies (middle 1/2) or a directly labeled anti-GST-DyLight649 antibody. *y*-axis shows relative AB intensity (arbitrary units); error bars indicate the standard deviation from an experiment performed with six replicate spots on the array. Full results are shown in Supplementary Fig S7.

Left: Immunofluorescence pictures of transfected YFP-TSPAN2 constructs in HEK293. Green: YFP-TSPAN2; blue staining: nuclei with DAPI. In contrast to TSPAN2-Y124F and TSPAN2-Y5-7F, TSPAN2-Wt and TSPAN2-124Y show many long cell protrusions, spread over a larger cell surface area and frequently form cell–cell contacts. Scale bars: 10 μM. Right: Quantification of the TSPAN2 cellular phenotype, characterized by spreading and formation of many membrane protrusions. Fraction of YFP-TSPAN2-transfected cells (efficiency 68–83%) with phenotype (membrane protrusions) was determined through blinded counting. YFP-TSPAN2wt: *n *= 503, 74.5%; TSPAN2-Y124F: *n *= 531, 5.6%; TSPAN2-124Y: *n *= 523, 58.2%; TSPAN2-Y5-7F: *n *= 551, 1.5%. Error bars show the standard deviation from the counting of 10 distinct images.

Left: Immunofluorescence pictures of YFP complementation assays with YFP-F1-TSPAN2 and YFP-F2-GRB2 or YFP-F2-PIK3R3, respectively. Green: YFP complementation that depends on the interaction of the YFP-F1- and YFP-F2-fusion proteins; blue staining: nuclei with DAPI. The GRB2-TSPAN2 interaction promoted long cell protrusions, the spreading over a larger cell surface area and frequent cell–cell contacts, while cells positive for the PIK3R3-TSPAN2 interaction hardly showed any cellular protrusions. Scale bars: 10 μM. Right: Quantification of the TSPAN2 cellular phenotype; fraction of PCA-positive transfected cells (efficiency 67–68%) with phenotype (membrane protrusions) was determined through blinded counting: YFP-F1-TSPAN2/YFP-F2-GRB2: *n *= 763, 39%; YFP-F1-TSPAN2/YFP-F2-PIK3R3: *n *= 667, 1.3%. Error bars show the standard deviation from the counting of 10 distinct images.

Source data are available online for this figure.

TSPANs like CD9 (the closest TSPAN2 relative) or CD151 are associated with many stages in tumor formation or increased metastasis in various cancers (Lazo, 2007; Hemler, 2014; Sadej *et al*, 2014). Recently, TSPAN2 has been shown to be involved in motility and invasion in lung cancer. High TSPAN2 expression levels, caused by p53 inactivation, are associated with the poor prognosis in lung adenocarcinomas (Otsubo *et al*, 2014). Similarly, other tetraspanins contribute to cytoskeletal dynamics. Loss of CD9 function influences cell migration (Castro-Sanchez *et al*, 2010) and expression of CD9 increases cell aggregation and promotes microvilli formation and spreading in different cell lines (Wang *et al*, 2011). Ectopic expression of CD151 induces apparently related phenotypes as transfected podocytes produced remarkable long, thin membrane processes (Blumenthal *et al*, 2012). We determined the subcellular localization of transiently expressed TSPAN2 in HEK293 cells and examined the cellular phenotype. YFP-TSPAN2 shows cytoplasmic and plasma membrane fluorescence, specifically highlighting membrane protrusions. Notably, expression of TSPAN2 induced a pronounced phenotype, where ∽75% of the transfected HEK293 cells formed many long membrane protrusions, spread over a larger surface area and built extensive cell–cell contacts (Fig4F). This cellular phenotype is reminiscent of the phenotype observed for ectopic expression of CD9 or CD151 in other human cells (Wang *et al*, 2011; Blumenthal *et al*, 2012). Formation of membrane extensions, spreading and cell–cell contact formation were strongly reduced when expressing the TSPAN2-Y124F (5.6%) and hardly observed with the TSPAN2-Y5-7F mutant (1.5%). The TSPAN2 version where all tyrosines except Y124 were replaced by phenylalanine (TSPAN2-124Y) was indistinguishable from wild-type TSPAN2 (Fig4F). This suggests that tyrosine 124, which is critical for the phosphorylation-dependent interaction with GRB2 and PIK3R3, is also critical for inducing a membrane extension phenotype in HEK293 cells.

To monitor the interaction of GRB2 or PIK3R3 and TSPAN2 with regard to subcellular localization in intact cells, we employed a YFP-based protein complementation assay (PCA), where the fluorescence signal indicates interacting rather than co-localizing proteins (Michnick *et al*, 2007). The GRB2-TSPAN2 interaction was observed at the plasma membrane predominantly at the long membrane protrusions of spreading cells (Fig4G). In contrast, the PIK3R3 interaction signal was located mainly in the cytoplasm. Importantly, only 1% of the PCA-positive HEK293 cells showed the YFP-labeled, TSPAN2-induced membrane extensions compared to 39% in the TSPAN2-GRB2 PCA (Fig4G). The specificity of the assay is indicated by assaying different GRB2/PIK3R3 interaction partners with PCA in intact cells corroborating our finding that different interactions of one protein can take place at very distinct subcellular locations (Supplementary Fig S10). We note that the effects of the interactions on the TSPAN2-induced phenotype may be enhanced by the reduced interaction dynamics of the YFP-based PCA system (Michnick *et al*, 2007). In our view, this experimental setup, which essentially pins conditional interactions, is advantageous for observing cellular edge phenotypes. It revealed that alternative interactions of TSPAN2 with its pY-interaction partners can led to a different phenotypic outcome (Fig4G). The GRB2-TSPAN2 interaction took place in structures distinct to the PIK3R3-TSPAN2 complex, where the former promoted spreading and membrane extensions and the latter apparently inhibited this phenotype. Since the phosphorylation of the same tyrosine residue is critical for GRB2 and PIK3R3 binding, the experiments suggest that interaction specificity, rather than kinase or phosphatase specificity, may determine the phenotype.

## Discussion

Protein interaction networks are essential in promoting our understanding of cellular phenotypes, and recording of a reliable, static representation of possible human protein–protein interactions is in progress (Stelzl, 2013; Rolland *et al*, 2014). However, conditional protein interaction rewiring is key to cellular responses (Ideker & Krogan, 2012; Woodsmith & Stelzl, 2014). Analysis of interaction rewiring provides mechanistic insight into cellular processes (Hegele *et al*, 2012) and a basis to assess the impact of genetic variation in disease (Zhong *et al*, 2009; Wei *et al*, 2014). In cellular responses, signals are often propagated through PTMs such as tyrosine phosphorylation. Reader proteins mediate these changing PTMs through their recognition (Seet *et al*, 2006), widely reshaping interaction networks in response to the signaling state of the cell.

This study presents a data set of 292 phospho-tyrosine-dependent interactions generated a by large-scale Y2H approach employing human tyrosine kinases (pY-Y2H) which covers part of an interaction space previously unamenable to direct experimental testing. Our approach assays kinase-dependent interactions of full-length proteins in a crowded cellular environment. To ensure specificity of the assay and avoid fitness defects of the yeast due to tyrosine kinase overexpression, bait, prey and protein kinases are expressed at very low levels (Worseck *et al*, 2012). The interaction patterns obtained show high specificity with respect to human kinases. Kinase-dependent yeast growth as indicator of phosphorylation-dependent interactions requires two recognition events, that is, prey phosphorylation by the kinase and binding of the phosphorylated protein by the phospho-recognition domain-containing pY-reader protein. Two recognition events decrease the chance of spurious or false-positive signals compared to non-conditional binary protein interactions. However, it is important to note that the interaction patterns observed depend on several parameters that can vary greatly between kinases and interacting pairs. This includes: (i) protein expression levels, (ii) kinase activity, (iii) kinase specificity and (iv) interaction specificity. For example, ABL2 promotes the highest number of pY-dependent protein interactions, maybe because its catalytic activity is optimal for the pY-Y2H assay.

Non-receptor tyrosine kinases are subject to tight regulation in human cell as they are typically inhibited in the absence of stimuli (Blume-Jensen & Hunter, 2001). It is prohibitively difficult to control the origin of phosphorylation in any human system as tyrosine kinases have localized activity levels, largely overlapping substrate specificity and function in signaling cascades. *Saccharomyces cerevisiae* does not contain *bona fide* tyrosine kinases, and any trans-regulatory components are not in place when screening for pY-PPI in yeast. However, in the pY-Y2H system, we unambiguously reveal kinase candidates which can phosphorylate human proteins, thereby promoting pY-dependent interactions. In an assay particularly designed to control for the absence of interactions, we tested a selected set of 37 interactions using kinase-dead versions of the nine tyrosine kinases used in the pY-Y2H screen. No comparable interaction signal was obtained with the kinase-dead versions for any of the tested interactions which led us conclude that the vast majority of the kinase-dependent interactions are indeed phosphorylation dependent in the pY-Y2H assay. Phospho-tyrosine binding proteins are multi-domain proteins, 24 of which contain a tyrosine kinase domain as well. Using them as bait, we found both phospho-dependent interactions and phospho-independent interactions. In the latter cases, we cannot exclude that these interactions are phosphorylation dependent as the phosphorylation of the interaction partner could be due to the kinase activity of the bait itself.

Network analysis of the data set confirms that phospho-tyrosine signaling has evolved as a highly connected modular system governing processes involved in cellular signaling, cell–cell communication and growth response pathways related to cancer (Lim & Pawson, 2010). Substrate binder annotation statistics also revealed strong agreement with our current knowledge of pY function validating our pY-PPI network. The PPI search was performed through matrix screening examining ∽13,900 proteins (Entrez GeneID level, covered by ∽17,000 ORFs) in a highly parallel fashion searching for pY-dependent binary relationships in a biologically unbiased manner. Nevertheless, we identified pY-dependent interactions revolving around a module of relatively few interacting proteins in the human proteome in agreement with the observation that PTMs, including tyrosine phosphorylation, selectively accumulate on human protein complexes (Woodsmith *et al*, 2013). However, widespread Y-phosphorylation was reported on more than 5,000 human proteins in large-scale phospho-proteomics studies (Tan *et al*, 2009; Hornbeck *et al*, 2012) and our literature analysis indicates that many more human pY-PPIs are still to be discovered. A fraction of the recorded pY sites may be spurious mass spectrometry identifications or may not function through recognition by pY-reader proteins. However, as with any interaction detection method, false negatives can arise in the pY-Y2H approach. Low expression levels of the hybrid proteins (Venkatesan *et al*, 2009; Worseck *et al*, 2012), kinases insufficiently active under the conditions used, and the fact that only a subset of the human non-RTKs was tested limits coverage of pY-mediated interactions.

Using known phospho-tyrosine sites in known linear amino acid motifs, we predicted a fraction of about 1/6 of the interactions likely mediated through linear peptide motif recognition. Supported by the high fraction of interactions that can be validated in co-IPs in mammalian cells, the data suggest that unknown linear epitopes surrounding the phospho-tyrosine sites or the full-length protein context are crucial for interaction specificity. Thus, this data set provides a wealth of pY-dependent links to investigate potentially novel modes of pY recognition case by case. For example, the SH2 domain of GRB2 is known to bind [pYxNx] peptides that form a β-turn conformation with the specificity-determining N(+2) residue next to W121 in the GRB2 EF-loop closing the binding side (Rahuel *et al*, 1996). However, a series of peptides with amino acids other than N at the (+2) positions (including A, D, E, G, H, I, M, P, Q and R) have been found to bind the GRB2(SH2) domain in systematic array studies with peptides resembling known pY sites (Miller *et al*, 2008; Tinti *et al*, 2013). In a series of pull-down experiments (Fig4A–D) and including a standard peptide binding array with purified proteins (Fig4E and Supplementary Fig S9), we identified Y124 of TSPAN2 as a critical site for GRB2 and PIK3R3 binding. Also in this case, the amino acid sequence surrounding TSPAN2-Y124 may not support binding of the pY peptide in the canonical binding mode (Higo *et al*, 2013) suggesting further investigation.

The role of tetraspanins in cancer is connected to their interactions with integrins, matrix metalloproteases and receptor kinases (Sadej *et al*, 2014). For example, CD9 and CDC151, like other TSPANs, are directly associated with RTKs such as DDR1 (Castro-Sanchez *et al*, 2010), MET (Klosek *et al*, 2005; Franco *et al*, 2010) or EGFR (Murayama *et al*, 2004, 2008). On the basis of those reports, it can be speculated that TSPANs are targets of RTKs or of other yet unidentified TK activity (Bordoli *et al*, 2014). Information about tyrosine phosphorylation has been collected in large-scale phospho-proteomics data acquisition projects for 17 TSPAN family members (Hornbeck *et al*, 2012). In particular, TSPAN8 is reported to be phosphorylated at Y122 (Moritz *et al*, 2010), which aligns in the corresponding region with Y124 of TSPAN2. In A549 lung adenocarcinoma cells, it has been shown that silencing of CD151 has major effects on downstream tyrosine phosphorylation signaling events involving p130Cas, FAK, paxillin and Src (Yamada *et al*, 2008). CD151 or CD9 influence cytoskeletal dynamics inducing cellular spreading and long protrusions similar to the phenotype observed when transfecting TSPAN2 into HEK293 cells (Wang *et al*, 2011; Blumenthal *et al*, 2012). Otsubo *et al* demonstrated that RNAi-mediated TSPAN2 knockdown decreases cell motility and invasive activity in small airway epithelial cells, the putative origin of lung adenocarcinomas, and promotes survival in metastatic mouse models (Otsubo *et al*, 2014). Our data open up a possible function of phospho-tyrosine 124-mediated interactions related to TSPAN2's involvement in adenocarcinoma invasion and migration. However, as we do not directly demonstrate TSPAN2-Y124 phosphorylation *in vivo*, we cannot rule out indirect effects of the Y124F mutation, for example, on TSPAN2 glycosylation, related to the observed cellular phenotype. Thus, we provide a mechanistic entry point to unravel the effects of elevated TSPAN2 levels in cancer such as lung adenocarcinomas (Otsubo *et al*, 2014). The hypothesis is that conditional pY-dependent interactions with different adaptor proteins, GRB2 or PIK3R3, respectively, may be altered in cancer, suggesting further investigation of TSPAN2 and its interactions in the transition of cells to abnormal growth (Lazo, 2007; Hemler, 2014; Otsubo *et al*, 2014).

The more detailed characterization of the TSPAN2–GRB2 and TSPAN2–PIK3R3 interactions exemplarily demonstrates how PPIs are spatially constrained. The signaling status of the cell dictates pY-dependent interactions and can give rise to a multitude of differential PPI networks involving an overlapping set of proteins each specifying the pY-mediated signal flow under certain conditions. Signaling hubs, like GRB2 or PIK3R3, can mediate several distinct responses, triggered, for example, by elevated kinase activity or alterations in local protein concentration, simultaneously and possibly independently. Therefore, assessing the consequences of conditional interactions, that is, edge phenotypes, rather than alterations of proteins alone may prove important for a better understanding of the molecular changes that occur, for example, during cancer development. To this end, our pY-PPI data set may serve as a useful resource.

## Materials and Methods

### Y2H screening

The basic PPI interaction mating procedure was described in detail previously (Stelzl *et al*, 2005; Worseck *et al*, 2012). For the pY-Y2H analysis, each bait strain (L40ccU2: MATa, his3Δ200, trp1-901, leu2-3,112, ade2, lys2-801am, cyh2, can1, ura3::(lexAop)8-GAL1TATA-lacZ, LYS2::(lexAop)4-HIS3TATA-HIS3) was individually created by co-transformation with plasmids expressing bait constructs (pBTM116-D9) or human tyrosine kinases (pASZ-DM). The pASZ-DM plasmids are gateway-compatible destination vectors based on pASZ-11 (Stotz & Linder, 1990), with an ARS-CEN origin of replication, an ADE2 auxotrophy marker, a yeast CUP1 promoter, a yeast CYC1 terminator and an optional nuclear localization sequence (MGSRKAELIPEPPKKKRKVELGTAS) at the N-terminus of the gene of interest. Using independently transformed yeast colonies, groups of 192 bait strains, each containing one of eight SH2/PTB domain-containing ORFs with one tyrosine kinase (24 versions for each pY-reader bait, including 9 kinases in two vectors plus six vector controls), were grown individually, pooled and mated on YPD agar plates (30°C, 36 h) with one of up to ∽17,000 prey strains four times in 384 array format. Interacting bait–prey pairs were identified by growth on selective agar plates (SD5:-Leu-Trp-Ura-His-Ade, 30°C, 5–7 days). Only bait–prey pairs (1,223) that showed growth at least two times were considered for further evaluation and were tested in independent pairwise retest experiments (Worseck *et al*, 2012). In the retest, all bait/kinase combinations, including empty vector controls, were tested individually in duplicates with the prey strain. In this test, kinase-dependent interactions were well distinguished from kinase-independent interactions (Supplementary Fig S3).

#### Kinase plate assays

Pairs of bait and prey were co-transformed and mated against an array containing 31 human non-RTKs, 9 kinase-dead mutant constructs [rendered inactive by an arginine to methionine mutation in the ATP binding site (Varjosalo *et al*, 2008)] and five empty vector controls. Diploid strains were selected on SD3 agar plates (Leu-Trp-Ade, 30°C, 3–4 days) and subsequently transferred to selective agar plates (SD5:-Leu-Trp-Ura-His-Ade, 30°C, 5–7 days) in order to control the growth of strains that express kinases that do not promote the interaction (Supplementary Fig S4).

The PPI data are reported in Supplementary Tables S3 and S4 and have been submitted to the IMEx consortium (http://www.imexconsortium.org) and are available through the IntAct database (assigned identifier IM-22632).

### Co-immunoprecipitation assays in mammalian cells

ORFs were transferred to firefly-V5 fusion vectors (pcDNA3.1V5Fire-DM; “firefly-tag”) and to protein A fusion vectors (pcDNA3.1PA-D57; “PA-tag”) using standard Gateway cloning procedures. Protein expression was assessed by immunoblotting and luciferase assays. HEK293 cells stably expressing ABL2 kinase were obtained by Flp-mediated recombination with a single FRT site present in the HEK293 Flp-In TREX host cell line (Invitrogen, R78007). Kinase expression was induced by addition of doxycycline (1 μM, Sigma, D9891) to the media 24 h before harvesting. For luciferase-based co-IP assays (Hegele *et al*, 2012; Weimann *et al*, 2013), in a well of a 96-well plate, 3 × 10^4^ HEK293 cells were transiently transfected with a total 100 ng of plasmid DNA using Lipofectamine 2000 (Invitrogen, 52887). Forty-eight hours after transfection (i.e. 24 h after kinase induction), cells were lysed in ice-cold 100 μl lysis buffer H [50 mM Hepes (pH 7.4); 150 mM NaCl; 1 mM EDTA; 10% glycerol; 1% Triton X-100; 1% phosphatase inhibitor cocktail 2 (Sigma-Aldrich, P5726), protease inhibitor (Roche, 11836170001)] for 30 min at 4°C. Protein complexes were precipitated from 70 μl cleared cell extract in IgG-coated microtiter plates for 1 h at 4°C and washed three times with 100 μl ice-cold PBS. The binding of the firefly-V5-tagged fusion protein (co-IP) to the PA-tagged fusion protein (IP) was assessed by measuring the firefly luciferase activity in a luminescence plate reader [Beckmann DTX800, Bright-Glo Luciferase Assay (Promega)]. Assays were performed as triplicate transfections. Log_2_-fold change binding for the protein pair is calculated from relative luciferase intensities in comparison with background binding measured in parallel with the firefly-tagged and a non-related protein A fusion protein. Ratios larger than two and a Z-score larger than two are considered positive (Hegele *et al*, 2012; Weimann *et al*, 2013). The full co-IP data are reported in Supplementary Tables S8 and S9.

### Computational analyses

#### Literature comparison

All pairs of proteins interacting in this study, as well as each pair of two bait genes, were used to query the protein–protein interaction meta-database ConsensusPathDB, v19 (Kamburov *et al*, 2013) for reported interactions. The results were manually validated by reading all publications for each interaction until an actual experiment showing a physical interaction was found. Where possible, phosphorylation dependency was inferred liberally. For example, an interaction that required growth factor treatment would have been counted as phosphorylation dependent, even if this experiment is not a strict demonstration of pY dependency.

#### Over-representation analysis

For Gene Ontology terms, NESTs and pathways, the webserver provided on the ConsensusPathDB website, v21 (Kamburov *et al*, 2013), was used. The Entrez GeneIDs corresponding to the prey were analyzed against the Entrez GeneIDs corresponding to all prey in our matrix. For Gene Ontology terms, level four terms were analyzed; for pathways, all pathways reported in PID, Reactome, NetPath, Wikipathways, KEGG, INOH and Biocarta as provided in CPDB were used. NESTs have a radius of 1. The over-representation of bait genes and cancer genes was performed accordingly using R and the list of cancer genes provided by Futreal *et al* (2004). For each analysis, the *P*-value corrected for the number of tests is provided.

#### Clustering of kinase plate experimental results

The kinase plate assay results were clustered using the APCluster package for R. The similarity for each pair of interactions was calculated as the square of the number overlapping kinase spots divided by the product of the two total numbers of kinase spots, for the 14 kinase spots that came up most. The preference was set to the median globally.

#### Network statistical analysis

Network analysis and visualization were performed with Cytoscape, R and custom PERL scripts.

### TSPAN2 binding assays

#### GST pull-down

GRB2(SH2) (aa 75–160), GRB2 (FL, aa 1–217) and PIK3R3 (FL, aa 1–461) were produced as N-terminal GST fusion proteins in *E. coli* SCS1. Overnight cultures were inoculated into TB broth containing 100 μg/ml ampicillin and 34 μg/ml chloramphenicol and were grown at 37°C to a OD_600_ of 1. Protein expression was induced by addition of IPTG to 0.25 mM final concentration for 22 h at 18°C. For each binding reaction, 50 ml bacterial culture was harvested by centrifugation at 2,000× *g* for 20 min, resuspended in 0.5 ml lysis buffer B [50 mM Hepes (pH 7.4); 150 mM NaCl; 1 mM EDTA; 5% glycerol; 1 mM DTT; 1 mg/ml lysozyme; 0.5% Brij 58; protease inhibitor (Roche, 11836170001)] and lysed by sonication. The lysate was then cleared by a 30-min centrifugation at 20,000× *g*. Fractions of the lysates with comparable amount of soluble protein were mixed for immobilization in a final volume of 0.5 ml lysis buffer B with 50 μl of 50% glutathione agarose slurry and incubated for 1 h. The agarose beads were washed twice with lysis buffer B and resuspended as a 50% slurry in lysis buffer H [50 mM Hepes (pH 7.4); 150 mM NaCl; 1 mM EDTA; 10% glycerol; 1% Triton X-100; 1% Phosphatase Inhibitor cocktail 2 (Sigma-Aldrich, P5726), Protease Inhibitor (Roche, 11836170001)].

PA-fusions of TSPAN2 were transiently expressed in HEK293 cells stably expressing ABL2. For each binding reaction, 6 × 10^6^ HEK293 cells were harvested from a 10-cm dish after 24-h ABL2 induction by addition of 1 μM doxycycline or, alternatively, after unspecific pY-phosphatase inhibition by addition of 8.82 mM H_2_O_2_ for 15 min, respectively. The cells were washed twice with ice-cold PBS and resuspended in 0.5 ml lysis buffer H. After a 30-min incubation, the lysate was cleared by a 30-min centrifugation at 20,000× *g*. The lysate was incubated with immobilized GRB2/PIK3R3 proteins for 2 h, beads were washed twice with wash buffer [50 mM Hepes (pH 7.4); 150 mM NaCl; 1 mM EDTA; 10% glycerol; 1% Triton X-100], and proteins were eluted in 30 μl SDS-loading buffer. Bound proteins were resolved by SDS–PAGE and detected via immunoblotting. PA-tagged TSPAN2 was directly detected with a rabbit anti-goat HRP antibody (Invitrogen, 611620). As a control, the blots were stripped and subsequently re-probed with polyclonal anti-GST antibody (GEHealthcare, 27457701V).

#### Peptide array assays

Peptide arrays were custom-made PepStar peptide microarrays from JPT, Berlin. All peptides were contained in triplicates of duplicates in both phosphorylated and non-phosphorylated versions, including control peptides for direct antibody recognition (total ∽1,200 peptides per slide). Peptide arrays were probed with serial dilutions of purified GST-GRB2, GST-GRB2(SH2) or GST-PIK3R3 proteins according to the manufacture's recommendations. Binding was visualized either with a directly fluorescence-labeled anti-GST-DyLight649 (Biomol 600-143-200) antibody or using combinations of primary and secondary antibodies. The fluorescence signal of the microarray was recorded with an Affimetrix 428™ array scanner.

### Immunofluorescence microscopy

HEK293 cells were seeded in a 24-well plate on poly-D-lysine-coated cover slips and transfected with plasmids encoding tagged proteins [i.e. protein A, FLAG, YFP or YFP-F1/F2; F1: aa 1–158; F2: aa 159–239 of Venus YFP (Stefan *et al*, 2011)] for protein fragment complementation, respectively. Twenty-four hours post-transfection, cells were fixed with 2% paraformaldehyde for 20 min, permeabilized with 0.2% Triton X-100 for 10 min and incubated with the specific antibody for 1 h at room temperature. Thereafter, nuclei were counter-stained with DAPI. For immuno-staining of endogenous proteins, the immuno-labeling step with the primary antibody was done overnight. For protein fragment complementation assay (PCA) and YFP detection, the permeabilization and immuno-labeling step were omitted. Fixed and stained cells were viewed with a confocal microscope LSM700 (Zeiss).

### Antibodies

The antibodies used were as follows: pAB anti-GST (goat, GE Healthcare, 27457701V), mAB anti-Flag M2-peroxidase HRP (mouse, Sigma, A8592), secondary mAB anti-goat HRP (rabbit, Invitrogen, 611620), secondary mAB anti-mouse (sheep, GE Healthcare, LNA931V), mAB anti-phospho-tyrosine clone 4G10 (mouse, Millipore, 05-321), pAB anti-TSPAN2 (rabbit, Sigma, SAB2102591), mAB anti-GRB2 (mouse, BD, 610112), pAB anti-PIK3R3 clone N13 (goat, Santa Cruz, sc-48644), pAB anti-PIK3R3 clone Z-8 (rabbit, Santa Cruz, sc-423), pAB anti-PIK3R3 (rabbit, Abcam, ab37825), mAb anti-GST (mouse, Sigma G1160); DyLight 488-conjugated AffiniPure anti-mouse (rabbit, Dianova, 315-485-003), DyLight 488-conjugated AffiniPure anti-rabbit (donkey, Dianova, 711-485-152), anti-GST-DyLight649 (Biomol 600-143-200) and secondary pAB anti-mouse IgG-DyLight 649 (donkey, Dianova, 715-495-150).

## Supporting Information

Supplementary Table S9

Review Process File
