# Supplementary material for: Phospho-tyrosine dependent protein–protein interaction network
Source: Mol Syst Biol. 2015 Mar 26;11(3):0794. doi: 10.15252/msb.20145968 (PMC4380928; doi:10.15252/msb.20145968)
Supplement: Supplementary file 1 [file msb0011-0794-sd1.pdf]

Supplemental data, Grossmann & Benlasfer et al.

Suppl. Figure S1. Kinase activity in the modif-Y2H.

Suppl. Figure S2. Flow chart of the pY-Y2H screen.

Suppl. Figure S3. Retest plates (selective agar).

Suppl. Figure S4. Kinase plate pY-Y2H assays.

Suppl. Figure S5. Statistical annotation of prey proteins.

Suppl. Figure S6. Kinase plate assay for the PIK3R3-C10orf81/PLEKHS1 interaction.

Suppl. Figure S7. Data comparison with the literature.

Suppl. Figure S8. Luciferase based co-IP results with SH2-domain mutant versions of PIK3R3

Suppl. Figure S9. Peptide array results.

Suppl. Figure S10. PCA results of GRB2-RB1 and PIK3R3- C10orf81/PLEKHS1 interactions.

Suppl. Table S1: Modified Y2H literature**.**

Suppl. Table S2: Bait strains.

Suppl. Table S3: pY-PPIs (separate file).

Suppl. Table S4: pY-independent PPIs (separate file).

Suppl. Table S5: Overrepresentation analysis of cancer genes

Suppl. Table S6: Linear recognition motif matches.

Suppl. Table S7: Literature set of pY-PPIs (separate file).

Suppl. Table S8: Co-IP data (separate file).

Suppl. Table S9: Co-IP data of SH2-domain mutants (separate file).

Legends to suppl. Figures S1-S8

Suppl. Figure S1. Kinase activity in the pY-Y2H.

Western blot with 4G10 antibody recognizing tyrosine phosphorylation on yeast proteins from total lysate of yeast growing on selective media (SD5:-Leu-Trp-Ura-His-Ade). The FRS3-FRS3 homomeric interaction was independent of exogenous human protein kinases, while PIK3R3-IRS1 required the expression of an active tyrosine kinase in the pY-Y2H system. The kinase domain of the human non-receptor tyrosine kinase FYN was under control of the yeast CUP1 promoter and expression levels can be increased through addition of 20 µM Cu^{2+} in the media. Western blot analysis showed that basal expression levels of the kinase, the activity of which was indistinguishable from background signal, was sufficient to promote the pY-dependent PIK3R3-IRS1 interaction (Mothe et al, 1997). Activity of the kinase could be visualized on the blot where induced by Cu^{2+}. Coomassie-stained gel served as loading control.

Suppl. Figure S2. Flow chart of the pY-Y2H screen.

The flow diagram presents all essential details including references to figures and tables of the pY-Y2H screen. 1,801,847 possible interactions were tested leading to the report of 292 pY-dependent and 336 independent interactions. Subsets of the interactions were subject to various validation approaches (green boxes).

Suppl. Figure S3. Retest plates (selective agar).

Primary hits qualifying as likely indicating interactions (see Materials and Methods) were retested by mating each corresponding prey strain to a 384-well microplate corresponding to the bait pool from the primary screen, *i.e.* eight bait with 24 kinase and control constructs on the third plasmid in duplicate (two rows each). Positions of the kinase constructs are indicated in the legend. The 9 Kinases were expressed from a vector that adds a nuclear localization sequence (+NLS) or not (-NLS) at the N-terminus of the proteins. The indicated prey was thus tested against 8 bait (24 bait strains in duplicate each) on one 384 plate. Pairs of proteins interacting in a kinase independent manner showed two full rows of growth on selective agar (SD5:-Leu-Trp-Ura-His-Ade), while pY-dependent interactions resulted characteristic diagonal pairs (duplicates of one kinase) of growing yeast colonies. For example a single pair of colonies was seen for SPTB-SH2D2A, while in the case of DAPP1-WDR20 six different kinase constructs promoted the growth of pairs of colonies. Both kinase-dependent and independent interactions were thus well distinguished in the retest approach. (* GULP1-WDR20 was removed from the data, as GULP1 did not pass the re-sequencing of the bait clones in a subsequent step.).

Suppl. Figure S4. Kinase plate pY-Y2H assays.

Pairs of interacting bait and prey were prepared in haploid MATα yeast and mated against an array of MATa yeast carrying different kinase plasmids in microplate format. That is 34 NRTK constructs representative of 31 different NRTKs (all human 32 NRTKs (Blume-Jensen & Hunter, 2001) but JAK1 for which our cloning attempts failed) plus 9 kinase-deficient constructs (FYN, ABL2, TNK1, FRK, FES, PTK2, SYK, BMX and JAK2) and 5 empty vector controls (inset for positions). Relatively large amount of 5 µl suspension of diploid yeast was pipetted on selective agar using a robot to provide confidence in negative results. Differential growth signals with different kinases were specific to the interactions. The kinase plate assay which comprised 5 empty vector controls (position labelled in gray in the inset) and 9 selected kinase death mutant versions (kinases labelled in blue in the inset) demonstrated that the kinase-dependent interactions that showed a differential signal in the retest were indeed phosphorylation-dependent (as opposed to kinase-dependent). Kinases showed different levels of activity in terms of numbers of growth-enabled interactions, highly active kinases are marked red in the inset. The most active kinases were the Src family kinases FYN, HCK, YES1, BLK and FGR and the non-Src family kinases ABL2 and FER. Interacting pairs of bait and prey function with different numbers of kinases. For example, the interaction between RASA1 and OLIG1 is enabled by FER kinase only, while others, such as the interaction between PIK3R3 and C10orf81/PLEKHS1 function with 12 kinase constructs. However, kinase patterns are not subsets of each other, and thus cannot readily be explained by different levels of kinase activity. Rather, combinations of overlapping and mutually exclusive kinase pattern suggest kinase and interaction specificity in the assay.

In total, 37 interactions were successfully assayed in two independent replicates. The resulting growth patterns were evaluated. 14 kinases that gave rise to most of the signals (blue dots in the schema above the plate) were used to for affinity propagation clustering of the patterns. Affinity propagation clustering (Bodenhofer et al, 2011; Frey & Dueck, 2007) is a method for the identification of groups of similar elements in many data sets (input preference was set as the median of the similarity matrix, q=0.5). Similarity between interactions was calculated as the square of the intersection of kinases that promote both interactions divided by the product of the number of kinases that promote each of the two interactions. Six clusters of kinase patterns were obtained (exemplars are displayed with color underlay). Purple: TNK1-/SRC-; Yellow: TNK1+/SRC+; Orange: PTK2+(IRS1); Dark red: FYN+/FGR+; Green: TNK1-/SRC+, Light blue: ABL2/FER(OLIG1); NCK1-PIK3R3, SH2D1B-STAT4 and FYB-NCK2 remain unassigned. (Grb2* indicates that the experiment using the GRB2(SH2) domain construct is displayed).

Suppl. Figure S5. Statistical over-representation of prey proteins.

GO molecular function (**a**), GO biological process (**b**), pathway (**c**) and gene neighborhood (**d**) categories were tested for over-representation in the set of prey proteins with all prey contained in the matrix as background (ENTREZ Gene ID level) were assessed using tools implemented on the ConsensusPathDB website(Kamburov et al, 2013). The most significantly enriched annotations are shown ($p \leq 0.01$ after Bonferroni multiple testing correction). Bar color indicates the p-value of enrichment; bar length indicates number of genes in the network with a given annotation. The circle indicates that the category that is enriched in the prey set is also enriched in the bait set. In the gene neighborhood analysis (**d**) centers that are receptor (RTK) and non-receptor tyrosine kinases (NRTK) are indicated.

Suppl. Figure S6. Kinase plate assay for the PIK3R3-C10orf81/PLEKHS1 interaction.

Kinase plate assay (as described in **Suppl. Figure S3**) is shown for the pY-dependent interaction PIK3R3-C10orf81/PLEKHS1. PIK3R3 contains two very similar SH2-domains at the N- and C-terminus, respectively. Mutations of a conserved arginine in the SH2 domains is known abolish pY-binding (Bisson et al, 2011; Mayer et al, 1992). In the kinase plate assay R90L mutation in the N-terminal domain had no effect on the pY-dependent interaction, while the R383K mutation in the C-terminal SH2-domain abolished growth of the pY-Y2H stains on selective media. The PIK3R3-PIK3CA interaction between the catalytic and regulatory subunit of PI3 Kinase was independent of kinase activity.

Suppl. Figure S7. Comparison with the literature.

The protein interaction meta-database ConsensusPathDB (Kamburov et al, 2013) was used to mine 221 relevant interactions and their references in the pY-reader - pY-reader (bait / bait) search space, which is much better covered in the literature than the pY-reader – binding partner (bait / prey) space. We next surveyed the references to find at least one piece of evidence reported for a physical interaction between the two respective proteins. Thus we defined 147 literature-curated interactions that could be validated in the bait-bait space (**Suppl. Table S7**). This data set was compared to the set of interactions detected in this study. Eigthy-one protein-protein interactions were found in the bait / bait space, 51 phoshotyrosine-dependent and 30 independent ones. Of these, 15 overlapped with the literature-curated interaction set (marked red).

The comparison to the literature allowed the assessment of two interesting parameters. The first one was the novelty of the interaction set, i.e. the degree to which the detected interactions expand the protein interaction knowledge. The novelty is approximated by the ratio of interactions not covered by the literature and the

complete set. This means that about 81% of the interactions were novel. The total number of interaction in the search space can be estimated from the size of the two interaction sets and the overlap. If we assume the interactions found in this study and the interactions in the literature-curated set to be two samples independently drawn from a common pool of protein-protein interactions in the interaction space and the probability of finding an interaction to be uniform, the number of unknown interactions about 581 (grey box).

To get a better estimate of the novelty, the same databases used to generate the literature-curated interaction set were queried for the remaining of the detected interactions and controlled in the same way. Interestingly, there are only 35 interactions in the literature for the complete set of 628 interactions (5.46%). This indicates a relatively strong research bias towards interactions among phospho-tyrosine recognizing domain-containing proteins.

Suppl. Figure S8. Luciferase based co-IP results with SH2-domain mutant versions of PIK3R3

The amino acid exchange of the conserved arginine in the SH2 domain is known to reduce phosphorylation-dependent binding substantially. PIK3R3 contains two SH2-domains at the N- and C-termini, respectively. In luciferase based co-IP assays, we assayed R to K mutant versions of the individual domains as well as PIK3R3 that has both SH2-domains mutated simultaneously. Fold binding is normalized to wild type PIK3R3 binding (1) (See **Suppl. Table S9**).

Suppl. Figure S9. Peptide array results

Peptides of 15 amino acids containing a phosphorylated or non-phosphorylated tyrosine in position 8 were probed for binding with purified PIK3R3, GRB2-FL and GRB2(SH2) domain proteins. Y-axis shows relative fluorescence intensity (arbitrary units), error bars indicate the standard deviation from an experiment performed with six replicate spot on the array. All phospho-peptides did react with the pY-specific antibody 4G10 while the non-phosphorylated version did not give signals (grey bars).

A. Known GRB2 and PIK3R3 binding sites (from IRS1 or RAB11 respectively) that also reflect known linear binding motifs (Songyang et al, 1993; Tinti et al, 2013) for GRB2 (pYxN) and PIK3R3 (pYxxM) were used as positive controls. **B.** The 15-mer peptide surrounding Y124 of TSPAN2 bound GRB2, GRB2SH2 and PIK3R3 but not the matched non-phospho version. Some binding of PIK3R3 to the pY60 containing peptide was observed for PIK3R3, however mutation of Y60 to phenylalanine did not affect any other binding assay. All other peptides, although reactive with the 4G10 antibody, did not show binding signals.

Suppl. Figure S10. PCA results of GRB2/PIK3R3 interactions.

Two pY-dependent interactions assayed via YFP-protein complementation (Stefan et al, 2011) in intact Hek293 cells involving proteins GRB2 and PIK3R3. The interactions take place in distinct subcellular locations as compared to the TSPAN2 interactions. GRB2-RB1 in the nucleus, PIK3R3-C10orf81/PLEKHS1 primarily at the membrane and in the cytoplasm. Green: protein interaction signal from YFP complementation, blue: nuclei (DAPI), red: membrane (WGA).

Supplemental Table S1.

Selection of studies reporting pY-dependent interactions using a Y2H set-up. Please see, for example, also a special chapter reviewing "Three-Hybrid Systems" by Stynen et al. (Stynen et al, 2012).

Study	System/technique	Interaction	PMID	Ref.
Osborne, Biotechnology (Nat Biotechnol) 1995	Lyc or Lyn Kinase on a third plasmid	Fc epsilon RI(cytoplasmic tail)::SH2-interactions	9636306	(Osborne et al, 1995)
Keegan and Cooper, Oncogene 1996	Modified two hybrid technique	SHPTP2(tyrosine 580)::Grb7	8622870	(Keegan & Cooper, 1996)
Dombrosky-Ferlan, Oncogene 1997	Yeast three-way screen with active Lyn on pEG202	Cbl::p85 which depends on kinase-active LYN kinase	9160881	(Dombrosky-Ferlan, 1997)
Fuller, Biotechniques 1998	Yeast-Trihybrid: p56 lck, [MET3 promoter] integrated into the yeast chromosome (Ylck4.1)	p56 lck: TCRzeta::ZAP-70;<br>p56 lck: TCRzeta::Csk	9668981	(Fuller et al, 1998)
Marti, PNAS 1998	"double bait constructs"; LexA-Lck: LexA DNA-binding domain-LCK kinase domain (241– 509) fusion	CL42::SHP2; CL6::SHP2, CL6::p85a; NKAT3::SHP2; NKAT3::SHP2; NKAT4::GRB2; CD28::GRB2; CD28::p85a	9751747	(Marti et al, 1998)
Rocchi, Mol Endocrinol 1998	Co-expression of human insulin receptor beta (INSR) fragment and a LexA fusion protein	PIK3R1::GAB1 (Y472);<br>PTPN11::GAB1 (Y827)	9658397	(Rocchi et al, 1998)
Xu, J Biol Chem 1999	cytoplasmic domain of the IR, IGF1R, or IRR was fused to the LexA + LexA-IR-IRS3 fusions	IR-IRS1, IR-IRS2, IR-IRS3<br>IR-IRS::p85(PIK3R1);<br>2 -4 YXXM motifs in IRS3 required for p85 PPI	10329736	(Xu et al, 1999)
Delahaye, Endocrinology. 2000	IR coexpression	FRS2::PTPN11	10650943	(Delahaye et al, 2000)
Volpers, Meth Mol Biol 2000	Src family kinases on a third plasmid	T-cell receptor zeta /beta::Fyn-SH2, katalysed by Lyc and Src	11100482	(Volpers et al, 2001)
Warner, Biochem J 2000	Classical Y2H, active kinase is the bait	KDR (Y1175)::Sck-SH2 domain;<br>Flt1::Shc SH2 domain;<br>Flt1::Sck SH2 domain;	10749680	(Warner et al, 2000)
Sayos, Blood 2000	Fyn and SAP expressed from bi-cistronic pBridge vector	SAP::LY-9;<br>SAP::CD84;	11389028	(Sayos et al, 2001)
Ellis, J Immunol 2000	Tri-hybrid system: Ylck4.1	GRID(SH2)::CD28	10820259	(Ellis et al, 2000)
Yamada, J Biochem 2001	Two-and-a-half system: third plasmid expressing rat Ntrk2	PTPN11:: GRB2;<br>PTPN11:: SIRPA;	11432792	(Yamada et al, 2001)
Woodfield, Biochem J 2001	Tri-hybrid system: Ylck4.1	Interaction p85::b-catenin is negatively regulated by tyrosine phosphorylation	11716761	(Woodfield et al, 2001)
Sathish, J Immunol 2001	Tri-hybrid system: Ylck4.1	SHP-1 SH2 domain::p85beta; SHP-1 SH2 domain::LAIR-1; SHP-1 SH2 domain::PD-1	11160222	(Sathish et al, 2001)
Clark, Proteome Res 2002<br>Clark, Chem Bio Chem 2003<br>Clark, Chem Bio Chem 2005	Yeast tribrid systems as a measure of PTK activity (e.g. v-Abl and v-Src)	LexA /GFP-AAYANAAVE substrate::GRB2(SH2)	12645896<br>12512083<br>16003805	(Clark & Peterson, 2002; 2003; 2005)
Cao, J Biol Chem 2002	Co-expression of Abl	Caveolin(Y14)::CSK;<br>Caveolin(Y14)::TRAF2;	11805080	(Cao et al, 2002)
Sayos, BBRC 2004	Fyn and CD85j expressed from bi-cistronic pBridge vector	CD85j::Csk	15474475	(Sayos et al, 2004)
Ingley, J Biol Chem 2006	Co-expression of FL-Lyn (pRSADE-MET-Lyn)	CBP(Y381+409)::Lyn(SH2);<br>CBP(Y314)::CSK(SH2);<br>CBP(Y314)::SOCS1(SH2)	16920712	(Ingley et al, 2006)
Verbrugge, Eur J Immunol 2006	Tri-hybrid system: Ylck4.1	LAIR-1::Csk	16380958	(Verbrugge et al, 2006)
Sylvester, PLoS One 2010	3rd plasmid with Fyn kinase	ADAP(FYB)::NCK1; ADAP::NCK2;<br>ADAP::SLP76; ADAP::FYN(SH2)	20661443	(Sylvester et al, 2010)

Table S2: **Bait strains**

Symbol	GeneID	UniProtID	Number of Domains SH2	IRS1	PID	Number of Baits total	screene	with PPIs
ABL1	25	ABL1_HUMAN	1			0	0	0
ABL2	27	ABL2_HUMAN	1			1	1	1
ANKS1A	23294	ANKS1_HUMAN			1	1	1	1
ANKS1B	56899	ANS1B_HUMAN			1	2	2	0
APBA1	320	APBA1_HUMAN			1	0	0	0
APBA2	321	APBA2_HUMAN			1	1	1	0
APBA3	9546	APBA3_HUMAN			1	0	0	0
APBB1	322	APBB1_HUMAN			2	2	1	0
APBB2	323	APBB2_HUMAN			2	2	2	0
APBB3	10307	APBB3_HUMAN			2	2	2	1
APPL1	26060	DP13A_HUMAN			1	2	2	2
APPL2	55198	DP13B_HUMAN			1	1	1	1
BCAR3	8412	BCAR3_HUMAN	1			1	1	0
BLK	640	BLK_HUMAN	1			1	1	0
BLNK	29760	BLNK_HUMAN	1			2	1	0
BMX	660	BMX_HUMAN	1			0	0	0
BTK	695	BTK_HUMAN	1			1	1	1
CBL	867	A3KMP8_HUMAN	1			1	1	1
CBLB	868	CBLB_HUMAN	1			2	1	1
CBLC	23624	CBLC_HUMAN	1			2	2	0
CCM2	83605	CCM2_HUMAN			1	2	2	1
CHN1	1123	CHIN_HUMAN	1			2	2	0
CHN2	1124	CHIO_HUMAN	1			1	1	1
CISH	1154	CISH_HUMAN	1			1	1	0
CRK	1398	CRK_HUMAN	1			4	3	3
CRKL	1399	CRKL_HUMAN	1			2	1	1
CSK	1445	CSK_HUMAN	1			1	1	1
DAB1	1600	DAB1_HUMAN			1	0	0	0
DAB2	1601	DAB2_HUMAN			1	3	1	0
DAPP1	27071	DAPP1_HUMAN	1			2	2	2
DOK1	1796	DOK1_HUMAN		1		2	1	1
DOK2	9046	DOK2_HUMAN		1		2	1	1
DOK3	79930	DOK3_HUMAN		1		0	0	0
DOK4	55715	DOK4_HUMAN		1		1	1	1
DOK5	55816	DOK5_HUMAN		1		1	1	1
DOK6	220164	DOK6_HUMAN		1		1	1	0
DOK7	285489	DOK7_HUMAN		1		2	1	1
EPS8	2059	EPS8_HUMAN			1	1	1	1
EPS8L2	64787	ES8L2_HUMAN			1	1	1	0
FER	2241	FER_HUMAN	1			1	1	1
FES	2242	FES_HUMAN	1			2	2	0
FGR	2268	FGR_HUMAN	1			1	0	0
FRK	2444	FRK_HUMAN	1			2	2	0
FRS2	10818	FRS2_HUMAN		1		1	1	1
FRS3	10817	FRS3_HUMAN		1		2	2	1
FYN	2534	FYN_HUMAN	1			2	2	1
GRAP	10750	GRAP_HUMAN	1			2	1	0
GRAP2	9402	GRAP2_HUMAN	1			3	1	1
GRB10	2887	GRB10_HUMAN	1			1	1	1
GRB14	2888	GRB14_HUMAN	1			1	1	1
GRB2	2885	GRB2_HUMAN	1			3	2	2
GRB7	2886	GRB7_HUMAN	1			2	2	1
GULP1	51454	GULP1_HUMAN			1	0	0	0
HCK	3055	HCK_HUMAN	1			3	3	1
HSH2D	84941	HSH2D_HUMAN	1			1	1	1
INPP5D	3635	SHIP1_HUMAN	1			2	2	0
INPPL1	3636	SHIP2_HUMAN	1			0	0	0
IRS1	3667	IRS1_HUMAN		1		1	1	1
IRS2	8660	IRS2_HUMAN		1		0	0	0
ITGB1BP1	9270	ITBP1_HUMAN			1	2	2	2
ITK	3702	ITK_HUMAN	1			1	1	0
JAK1	3716	JAK1_HUMAN	1			0	0	0
JAK2	3717	JAK2_HUMAN	1			1	0	0
JAK3	3718	JAK3_HUMAN	1			1	1	1
LCK	3932	LCK_HUMAN	1			0	0	0
LCP2	3937	LCP2_HUMAN	1			2	2	0
LDLRAP1	26119	ARH_HUMAN			1	1	1	0
LYN	4067	LYN_HUMAN	1			3	3	1
MAPK8IP1	9479	JIP1_HUMAN			1	1	1	0
MAPK8IP2	23542	JIP2_HUMAN			1	2	2	1
MATK	4145	MATK_HUMAN	1			1	1	0
MIST	116449	CLNK_HUMAN	1			1	1	1
NCK1	4690	NCK1_HUMAN	1			2	2	2
NCK2	8440	NCK2_HUMAN	1			2	0	0
NOS1AP	9722	CAPON_HUMAN			1	1	1	1

Entrez Symbol	Entrez GeneID	Representative UniProtID	Number of Domains SH2	TB_IR	TB_PI	Number of Baits total	screene	with PPIs
NUMB	8650	NUMB_HUMAN			1	1	1	1
NUMBL	9253	NUMBL_HUMAN			1	0	0	0
PID1	55022	PCLI1_HUMAN			1	1	1	0
PIK3R1	5295	P85A_HUMAN	2			2	1	0
PIK3R2	5296	Q96CK7_HUMAN	2			1	1	1
PIK3R3	8503	P55G_HUMAN	2			2	2	1
PLCG1	5335	PLCG1_HUMAN	2			1	1	0
PLCG2	5336	PLCG2_HUMAN	2			1	1	1
PTK6	5753	PTK6_HUMAN	1			1	1	0
PTPN11	5781	PTN11_HUMAN	2			1	1	1
PTPN6	5777	PTN6_HUMAN	2			3	3	1
RABGAP1	23637	RBGP1_HUMAN			1	2	2	0
RABGAP1L	9910	RBG1L_HUMAN			1	3	3	1
RASA1	5921	RASA1_HUMAN	2			2	2	1
RGS12	6002	RGS12_HUMAN			1	0	0	0
RIN1	9610	RIN1_HUMAN	1			1	1	1
RIN2	54453	RIN2_HUMAN	1			1	1	0
RIN3	79890	RIN3_HUMAN	1			3	3	3
SH2B1	25970	SH2B1_HUMAN	1			1	1	1
SH2B2	10603	SH2B2_HUMAN	1			0	0	0
SH2B3	10019	SH2B3_HUMAN	1			0	0	0
SH2D1A	4068	SH21A_HUMAN	1			2	2	1
SH2D1B	117157	SH21B_HUMAN	1			3	3	2
SH2D2A	9047	SH22A_HUMAN	1			1	1	1
SH2D3A	10045	SH23A_HUMAN	1			1	1	1
SH2D3C	10044	SH2D3_HUMAN	1			1	1	0
SH2D4A	63898	SH24A_HUMAN	1			1	1	1
SH2D4B	387694	SH24B_HUMAN	1			0	0	0
SH2D5	400745	SH2D5_HUMAN	1			1	1	0
SH2D6	284948	SH2D6_HUMAN	1			0	0	0
SH3BP2	6452	3BP2_HUMAN	1			1	1	1
SHB	6461	SHB_HUMAN	1			0	0	0
SHC1	6464	SHC1_HUMAN	1		1	0	0	0
SHC2	25759	SHC2_HUMAN	1		1	0	0	0
SHC3	53358	SHC3_HUMAN	1		1	1	1	0
SHC4	399694	SHC4_HUMAN	1		1	1	1	0
SHD	56961	SHD_HUMAN	1			2	1	1
SHE	126669	SHE_HUMAN	1			1	1	1
SHF	90525	SHF_HUMAN	1			0	0	0
SLA	6503	SLAP1_HUMAN	1			2	1	0
SLA2	84174	SLAP2_HUMAN	1			1	0	0
SOCS1	8651	SOCS1_HUMAN	1			1	1	1
SOCS2	8835	SOCS2_HUMAN	1			1	1	0
SOCS3	9021	SOCS3_HUMAN	1			2	1	1
SOCS4	122809	SOCS4_HUMAN	1			1	1	1
SOCS5	9655	SOCS5_HUMAN	1			2	2	0
SOCS6	9306	SOCS6_HUMAN	2			1	1	1
SOCS7	30837	SOCS7_HUMAN	1			0	0	0
SRC	6714	SRC_HUMAN	1			2	2	2
SRMS	6725	SRMS_HUMAN	1			1	1	0
STAP1	26228	STAP1_HUMAN	1			1	1	0
STAP2	55620	STAP2_HUMAN	1			1	1	0
STAT1	6772	STAT1_HUMAN	1			1	1	1
STAT2	6773	STAT2_HUMAN	1			0	0	0
STAT3	6774	STAT3_HUMAN	1			4	2	2
STAT4	6775	STAT4_HUMAN	1			0	0	0
STAT5A	6776	STA5A_HUMAN	1			2	1	1
STAT5B	6777	STA5B_HUMAN	1			2	0	0
STAT6	6778	STAT6_HUMAN	1			1	1	0
SUPT6H	6830	SPT6H_HUMAN	1			0	0	0
SYK	6850	KSYK_HUMAN	2			2	1	1
TBC1D4	9882	TBCD4_HUMAN			2	1	1	0
TEC	7006	TEC_HUMAN	1			1	1	0
TENC1	23371	TENC1_HUMAN	1		1	1	1	1
TNS1	7145	TENS1_HUMAN	1		1	0	0	0
TNS3	64759	TENS3_HUMAN	1		1	1	1	0
TNS4	84951	TENS4_HUMAN	1			1	1	1
TXK	7294	TXK_HUMAN	1			1	1	0
TYK2	7297	TYK2_HUMAN	1			1	1	0
VAV1	7409	VAV_HUMAN	1			1	1	0
VAV2	7410	VAV2_HUMAN	1			1	1	0
VAV3	10451	VAV3_HUMAN	1			1	1	0
YES1	7525	YES_HUMAN	1			1	1	1
ZAP70	7535	ZAP70_HUMAN	2			2	2	0
					**total**	**188**	**159**	**82**

Supplemental Table S5: Overrepresentation analysis of cancer genes.

The cancer genes (Futreal et al, 2004) were tested for over-representation in the protein-protein interactions using the hypergeometric test. The analysis was performed on the basis of cancer genes found as prey and on the basis of interactions involving cancer genes as prey. For the first part, the number of total prey genes screened (prey matrix size), cancer genes screened (cancer prey genes total), total number of prey genes interacting with at least one bait (prey genes found) and the number of cancer prey genes interacting with at least one bait (cancer prey genes found) were determined for all interactions found in this study (complete network) and all phosphotyrosine-dependent interactions found in this study (phosphotyrosine- dependent only). For the second part, the number of bait genes interacting with at least one prey gene (bait genes) was multiplied with the number of prey genes to calculate the total number of interactions (interactions possible) and the number of interactions involving cancer prey genes (cancer interactions possible) that could have been found. These values, and the actual number of total interactions (interactions found) and interactions involving cancer prey genes (cancer PPIs found) found in this study were used to calculate the probability of seeing cancer gene enrichment by chance alone (p-value).

	complete network	phosphotyrosine-dependent only
prey matrix size	13931	13931
cancer prey genes total	303	303
prey genes found	366	187
cancer prey genes found	22 *	13 *
p-value	2.38×10^{-5}	2.83×10^{-4}
bait genes	70	59
interactions possible	975170	821929
cancer interactions possible	21210	17877
interactions found	628	292
cancer PPIs found	64	35
p-value	1.96×10^{-23}	1.13×10^{-15}

* ABL2(pY), AKT1(npY), CBL(both), CBLB(both), CBLC(pY), DDX5(npY), EVI1(pY), EZH2(npY), FANCG(npY), FOXO1A(npY), GATA1(npY), LASP1(both), LCK(both), PAFAH1B2(both), PER1(npY), PIK3CA(npY), PIK3R1(both), RB1(both), SEPT6(npY), SOCS1(pY), TP53(both), TSC1(both)

Supplemental Table S6. Motif matches

			pY-dependent			independent			
BaitGeneID	BaitSymbol	Motif	1	2	3+	1	2	3+	
27	ABL2	[pY][E/T/M][N/E/D][P/V/L]	2	1					(Songyang et al, 1993)
867	CBL	[N][X][pY][S/T][X][X][P]	3	5		4	1		(Lupher et al, 1997)
1398	CRK	[pY][D/K/N][H/F/R][P/V/L]	16	19	7	18	10		(Songyang et al, 1993)
2534	FYN	[pY][E/T][E/D/Q][I/V/M]				1	3		(Songyang et al, 1993)
2887	GRB10	[F/Y][pY][E/T/Y/S][N][I/L/V/P/T/Y/S]	1		1				(Rodriguez et al, 2004)
2885	GRB2	[pY][Q/Y/V][N][Y/Q/F]	7	4		1	1		(Songyang et al, 1993)
2885	GRB2	[V][pY][Q][N][W/F]	6	2		1	1		(Dente et al, 1997)
2885	GRB2	[pY][I/V][N][I/L/V]	9	6		1	2	1	(Rodriguez et al, 2004)
2886	GRB7	[F/Y][pY][E/T/Y/S][N][I/L/V/P/T/Y/S]		2	1				(Rodriguez et al, 2004)
3932	LCK	[pY][E/T/Q][E/D][I/V/M]	1	1		1			(Songyang et al, 1993)
8503	PIK3R3	[pY][M/I/V/E][X][M]	22	7		16	1		(Songyang et al, 1993)
8503	PIK3R3	[pY][M/L/I][X][M]	22	2		17	2		(Songyang et al, 1993)
5781	PTPN11	[I/L/V][I/L/V][I/F/V][pY][T/V][I/L][I/L/V/P]			1				(Rodriguez et al, 2004)
5777	PTPN6	[L][Y/H][pY][M/F][X][F/M]	1						(Beebe et al, 2000)
5777	PTPN6	[V/I/L][X][pY][A][X][L/V]	1	1					(Beebe et al, 2000)
5777	PTPN6	[pY][F][X][F/P/L/Y]	1						(Songyang et al, 1994)
4068	SH2D1A	[T][I][pY][X][X][V/I]	2	1		4			(Poy et al, 1999)
117157	SH2D1B	[T][I][pY][X][X][V/I]	1			2	1		(Poy et al, 1999)
6452	SH3BP2	[pY][E/M/V][N/V/I][X]	2						(Songyang et al, 1994)
6714	SRC	[pY][EDT][ENY][IML]		1			1		(Songyang et al, 1993)
6774	STAT3	[pY][X][X][Q]	15				5		(Stahl et al, 1995)
6850	SYK	[pY][Q/T/E][E/Q][L/I]		1					(Songyang et al, 1994)
7145	TNS1	[pY][D/E][N][I/F/V]						1	(Auger et al, 1996)
			78	47	10	49	27	2	

Suppl Figure S1

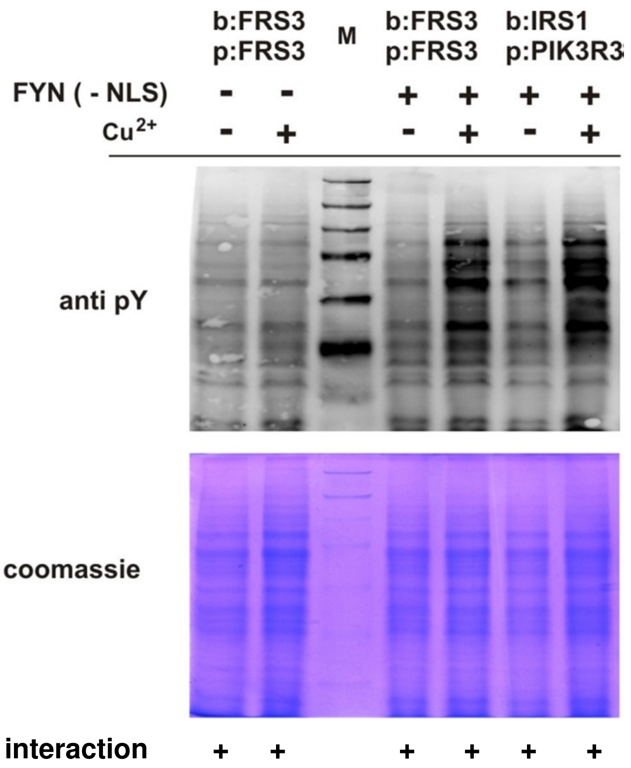

Suppl Figure S2

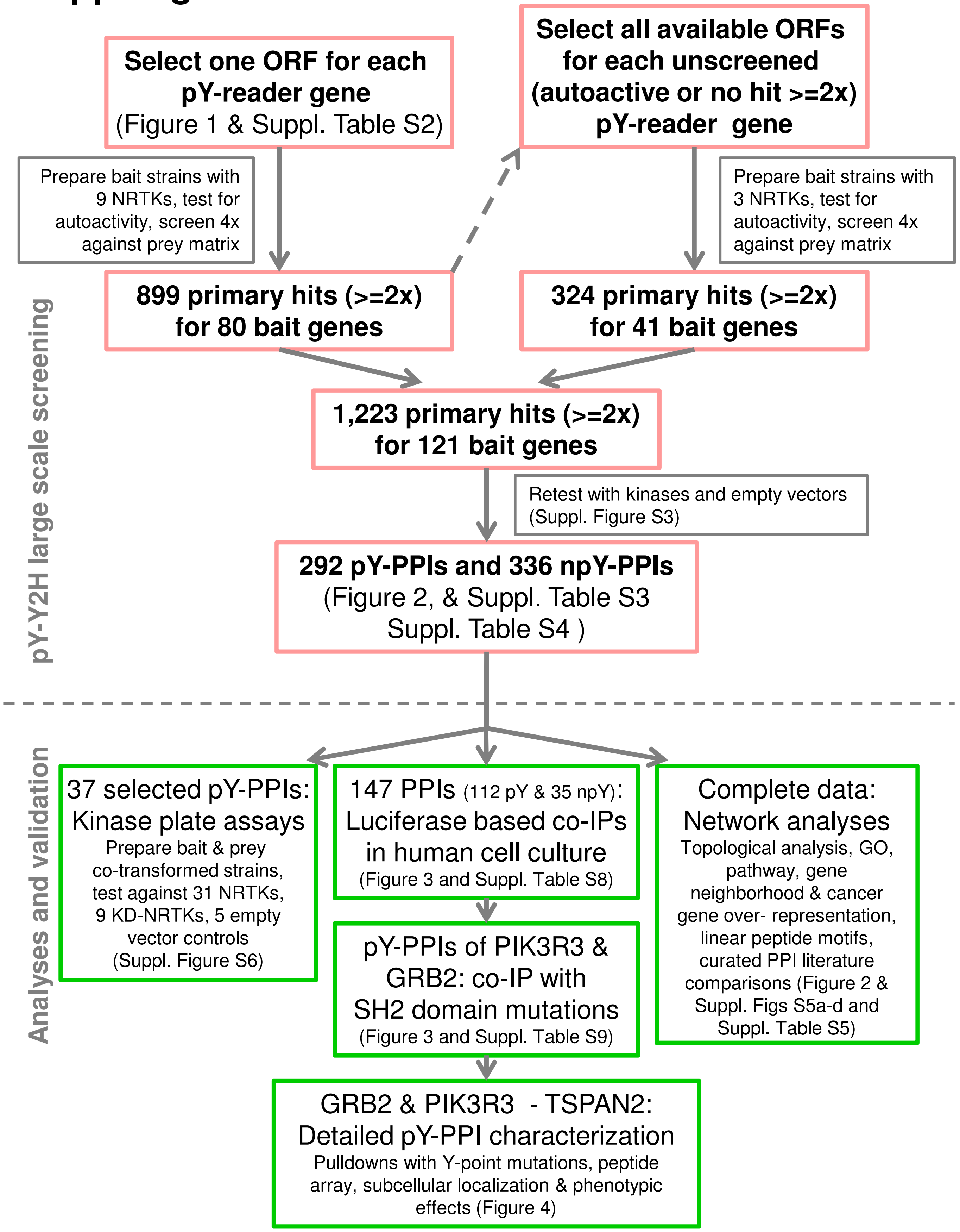

Suppl Figure S3

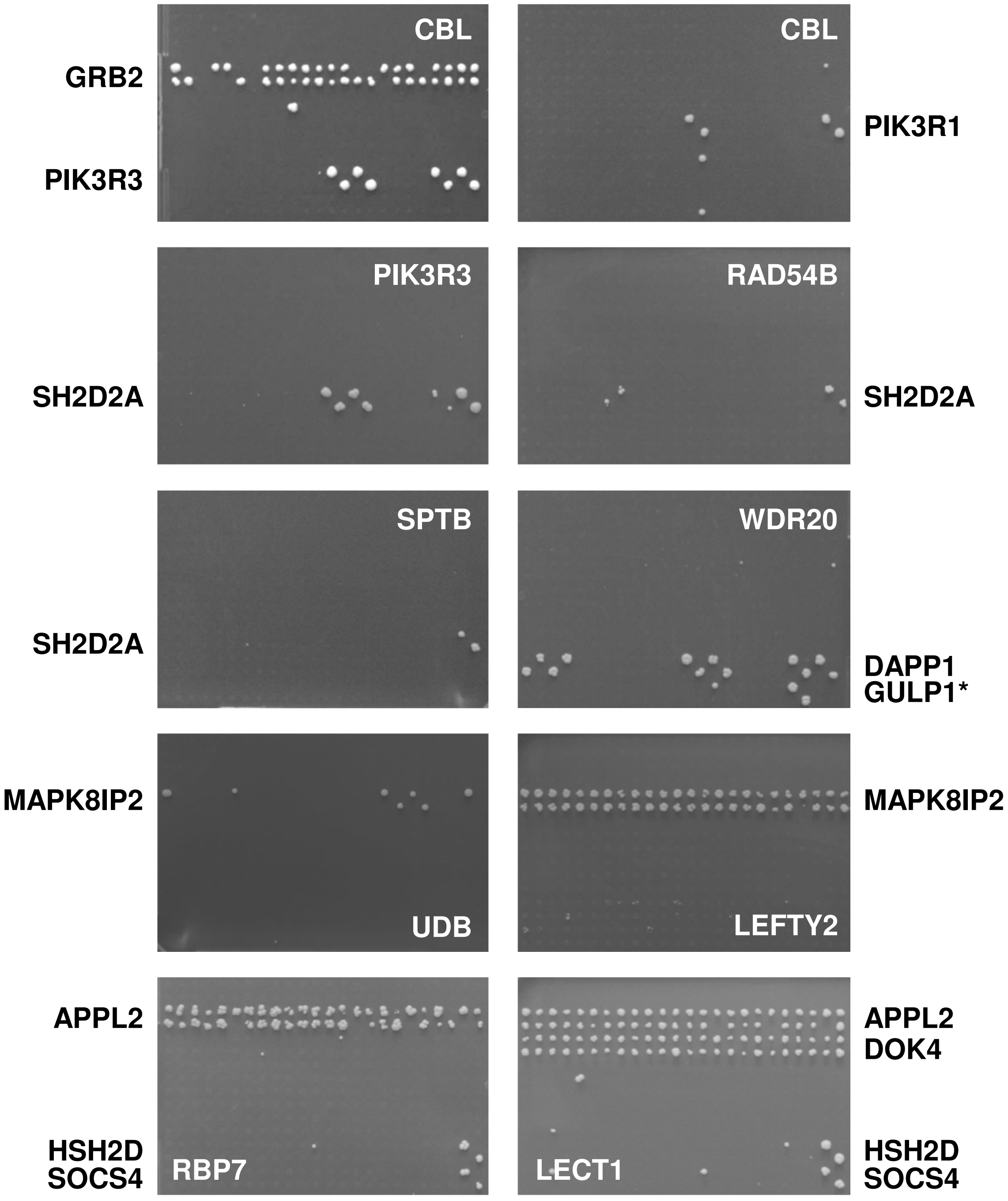

BAIT																								
	1	2	3	4	5	6	7	8	9	10	11	12	13	14	15	16	17	18	19	20	21	22	23	24
	2	1	4	3	6	5	8	7	10	9	12	11	14	13	16	15	18	17	20	19	22	21	24	23

	Kinase	NLS
1	PTK2	-
2	FRK	-
3	PTK2	+
4	FRK	+
5	SYK	-
6	TNK1	-
7	SYK	+
8	TNK1	+
9	BMX	-
10	FES	-
11	BMX	+
12	FES	+
13	FYN	-
14	empty	-
15	FYN	+
16	empty	+
17	JAK2	-
18	empty	-
19	JAK2	+
20	empty	+
21	ABL2	-
22	empty	-
23	ABL2	+
24	empty	+

Suppl Figure S4

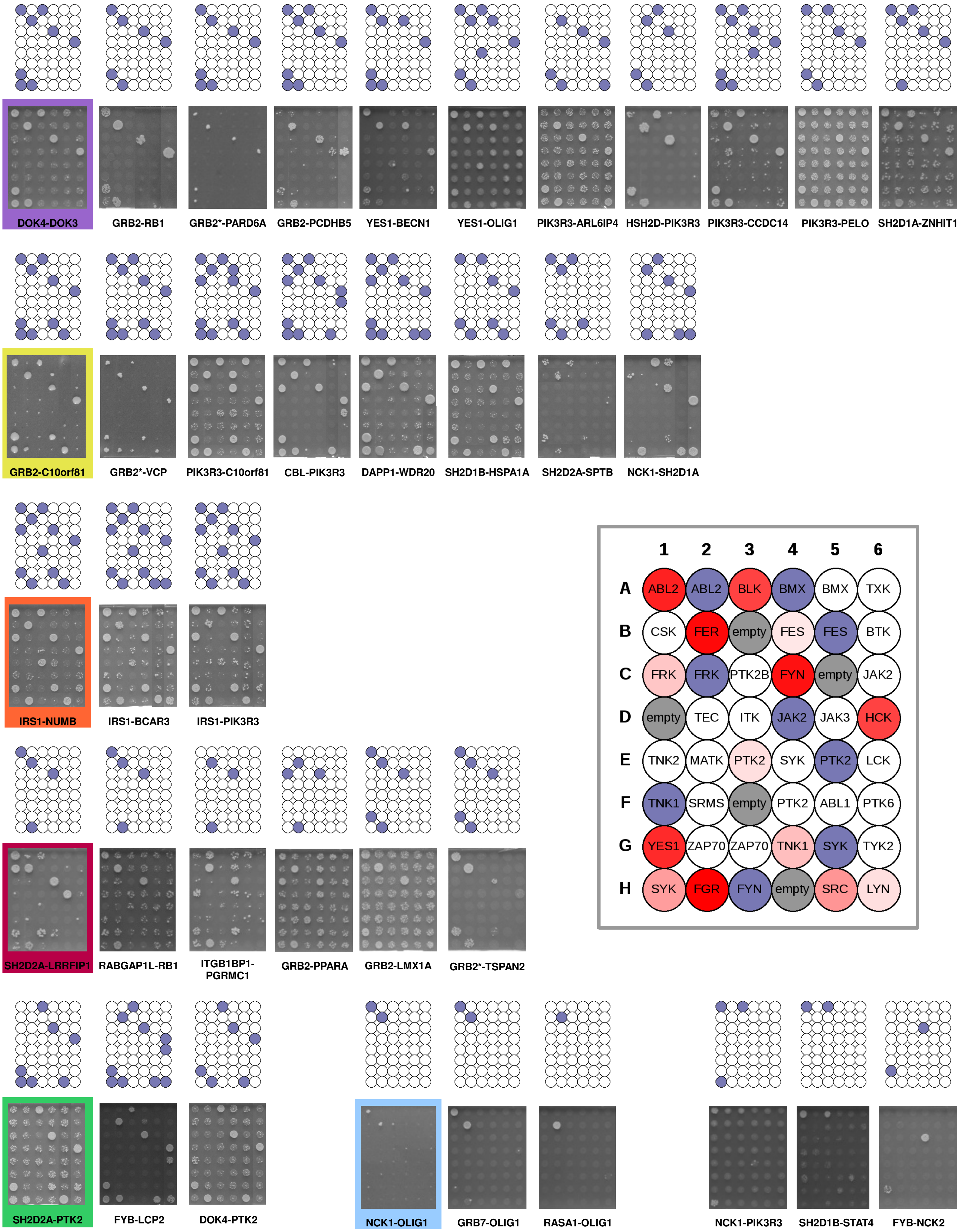

Suppl Figure S5a: GO Function

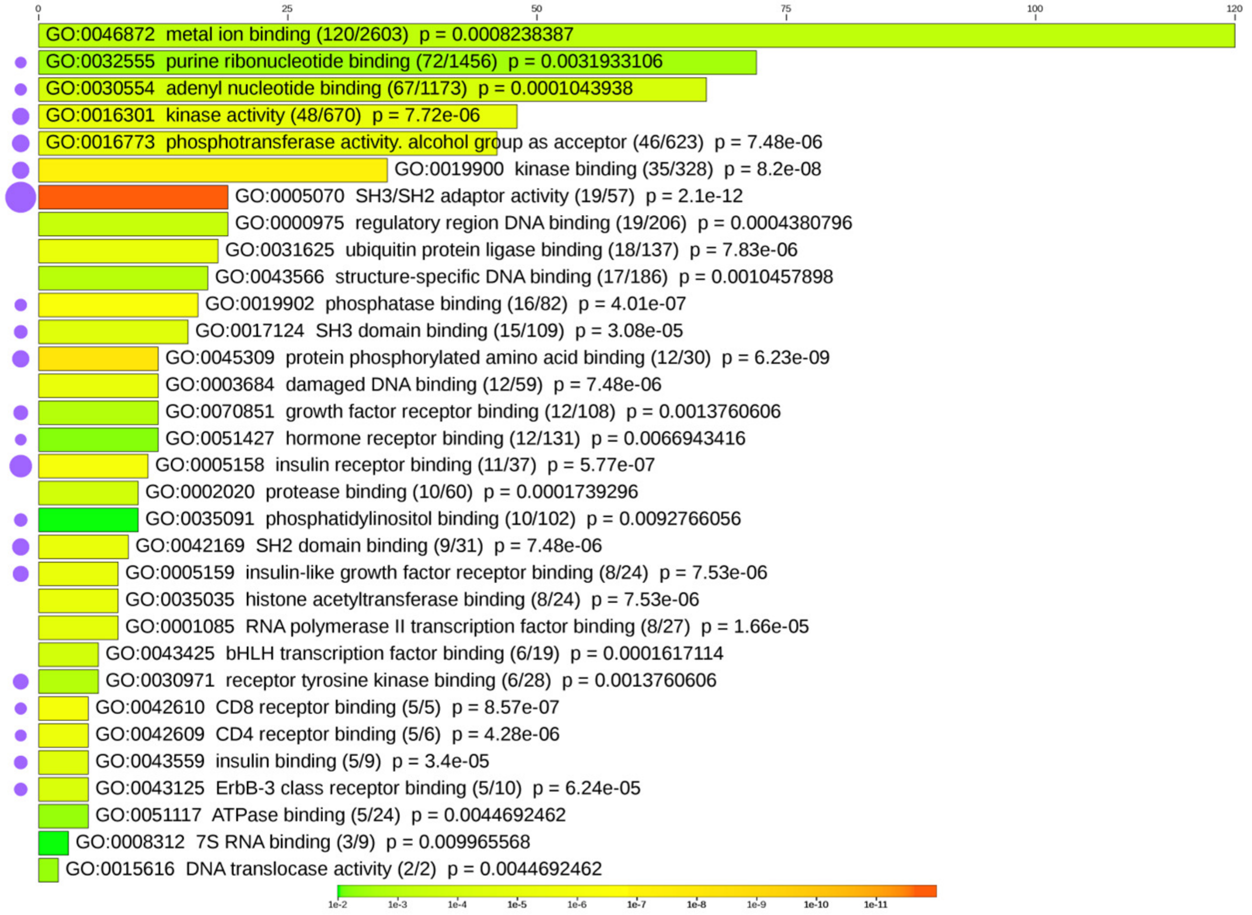

Suppl Figure S5b : GO Process

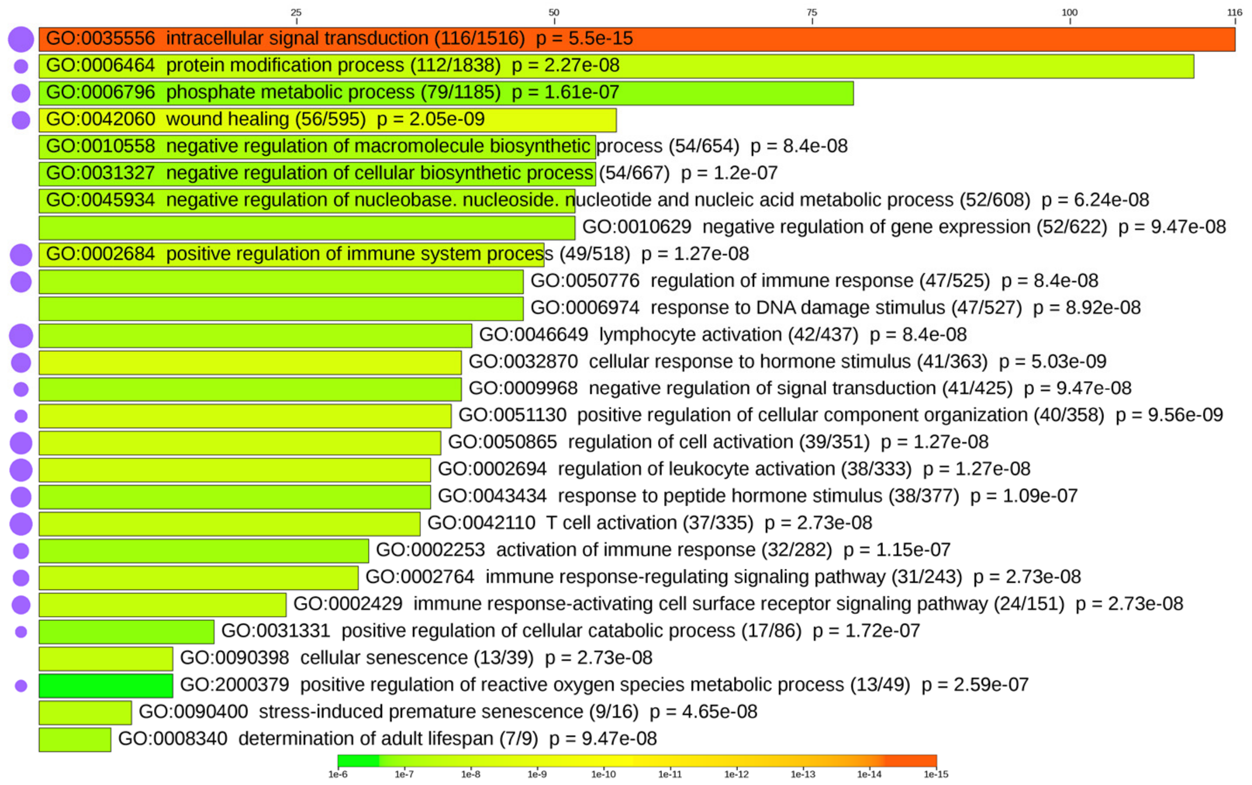

Suppl Figure S5c : Pathways

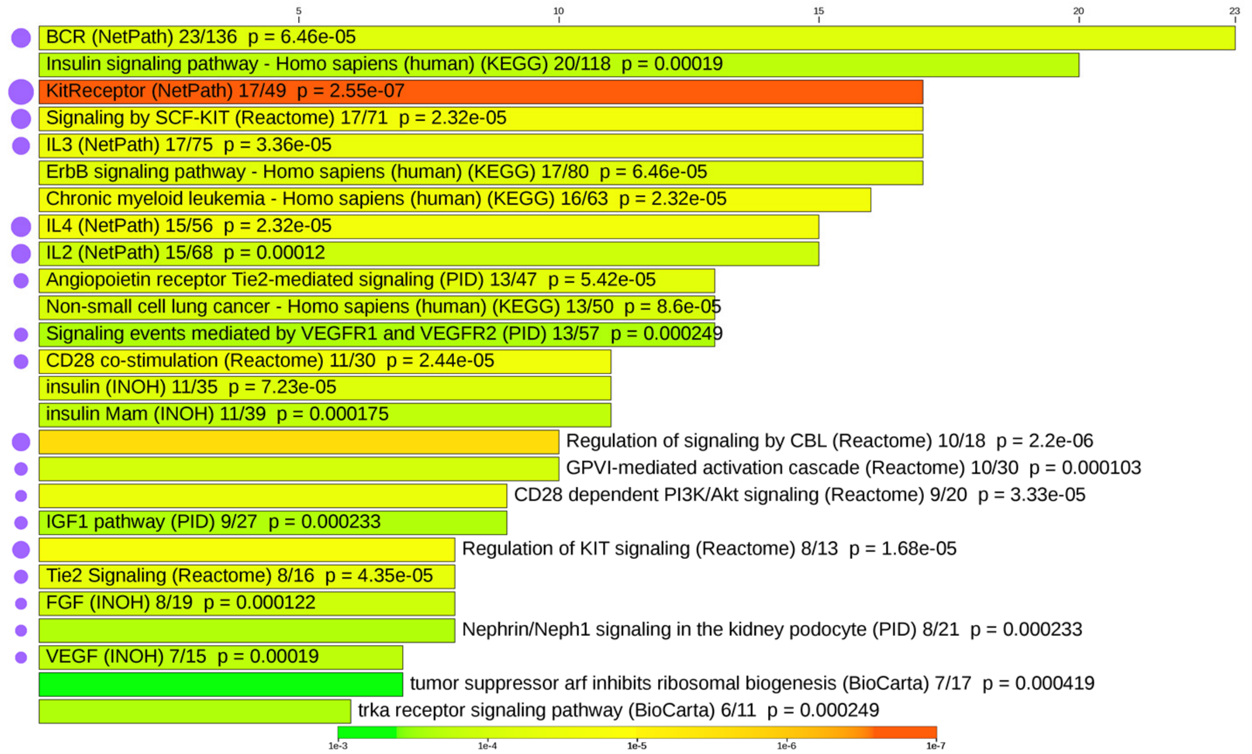

Suppl Figure S5d : Gene neighborhood

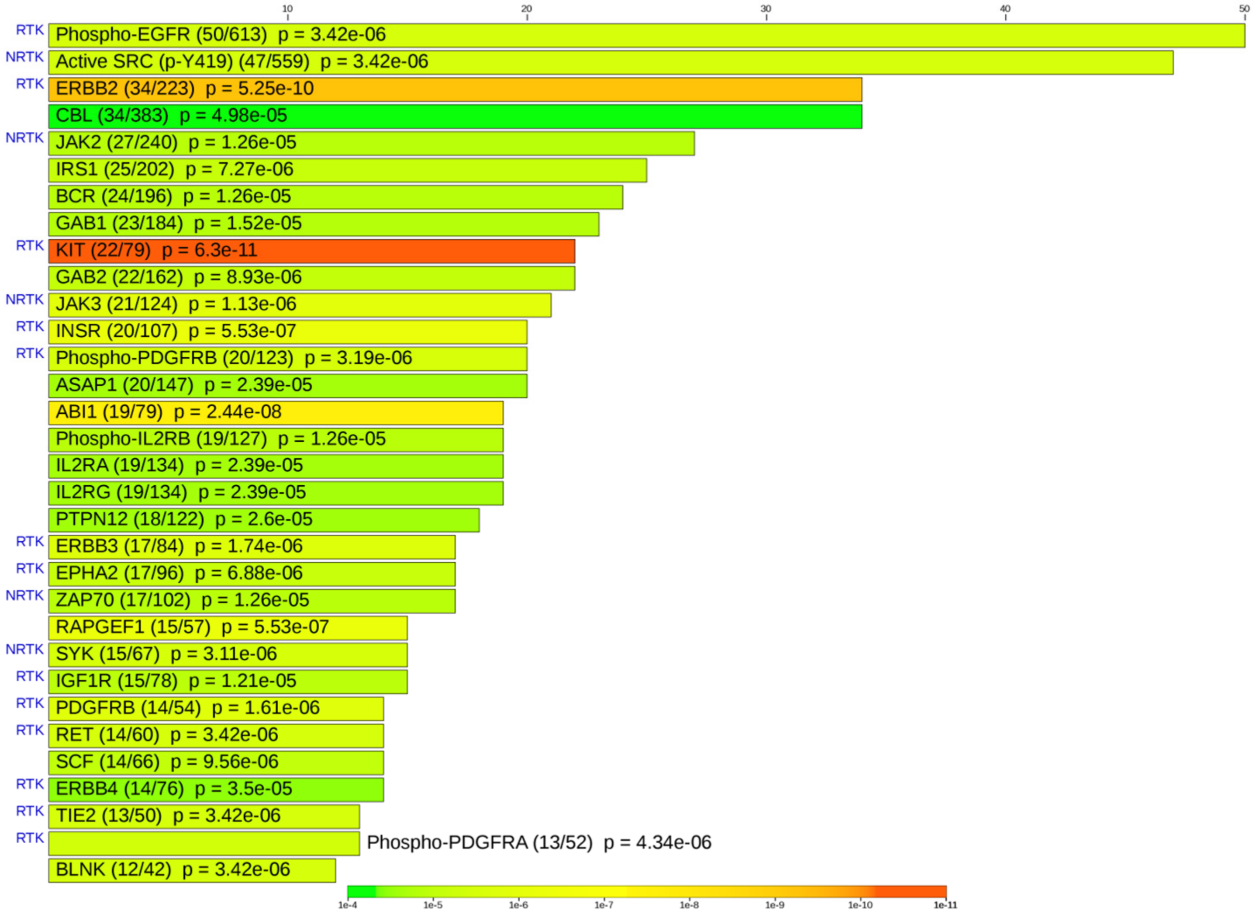

Suppl Figure S6

ABL2	ABL2-KD	BLK	BMX-KD	BMX	TXK
CSK	FER	ctrl	FES	FES-KD	BTK
FRK	FRK-KD	PTK2B	FYN	ctrl	JAK2
ctrl	TEC	ITK	JAK2-KD	JAK3	HCK
TNK2	MATK	PTK2	SYK	PTK2-KD	LCK
TNK1-KD	SRMS	ctrl	PTK2	ABL1	PTK6
YES1	ZAP70	ZAP70	TNK1	SYK-KD	TYK2
SYK	FGR	FYN-KD	ctrl	SRC	LYN

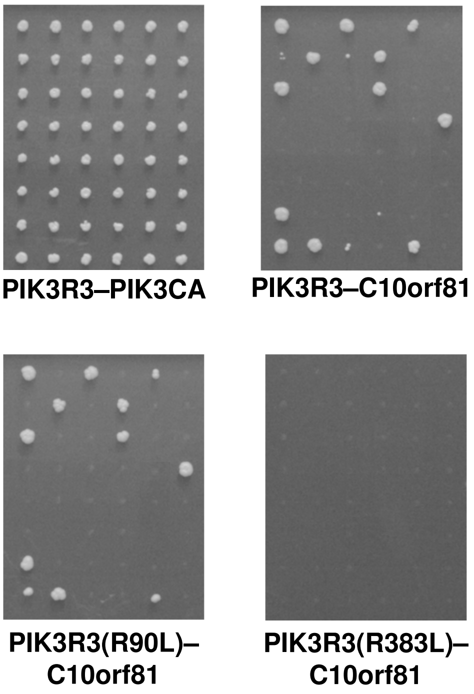

Suppl Figure S7

Overlap with phospho-tyrosine-recognizing domain interaction set		Total	Literature curated: dependent	independent	both	no information	Total
	Bait-Bait Total		23	31	5	88	**147**
Bait-Bait	Dependent	**51**	3	2	0	5	**10**
	Independent	**30**	0	3	1	1	**5**
	**Total**	**81**	**3**	**5**	**1**	**6**	**15**
Complete	Dependent	**292**	7	4	0	7	**18**
	Independent	**336**	3	5	1	8	**17**
	**Total**	**628**	**10**	**9**	**1**	**15**	**35**

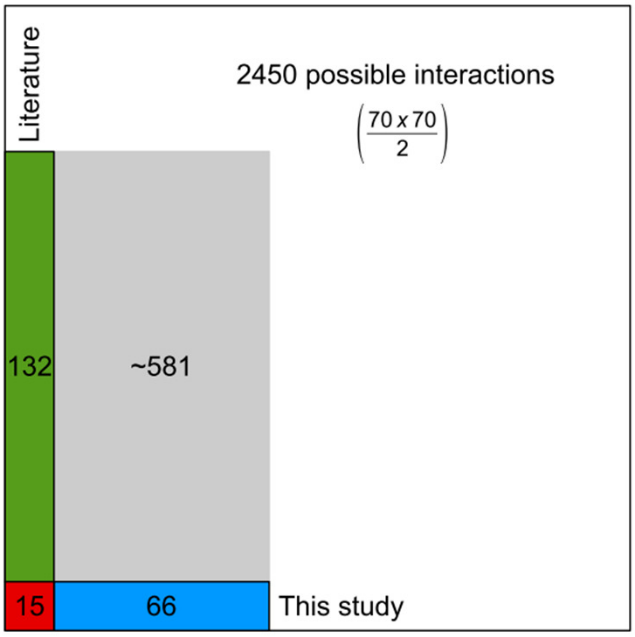

Suppl Figure S8

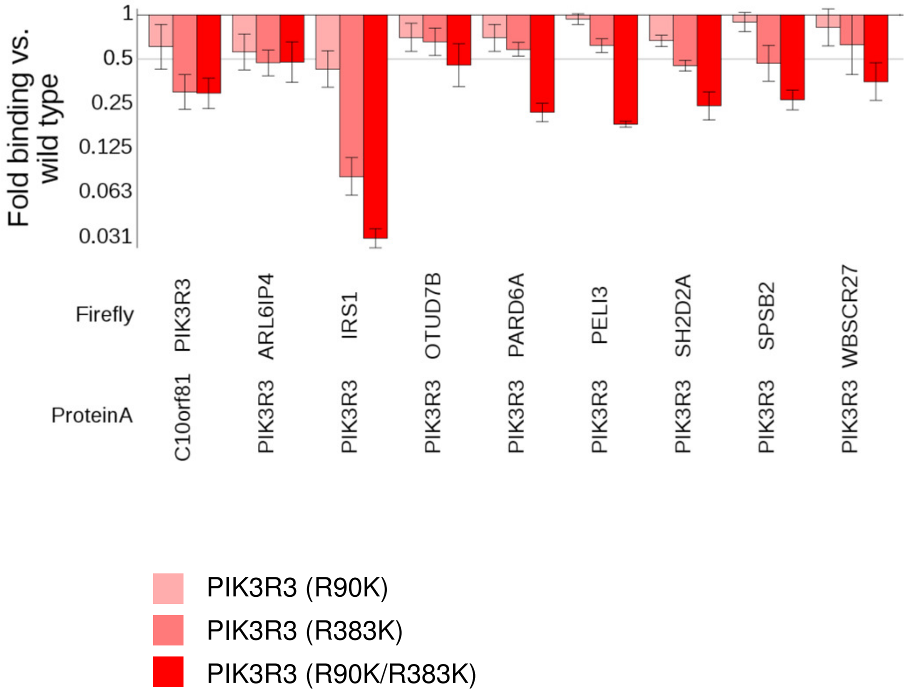

Suppl Figure S9

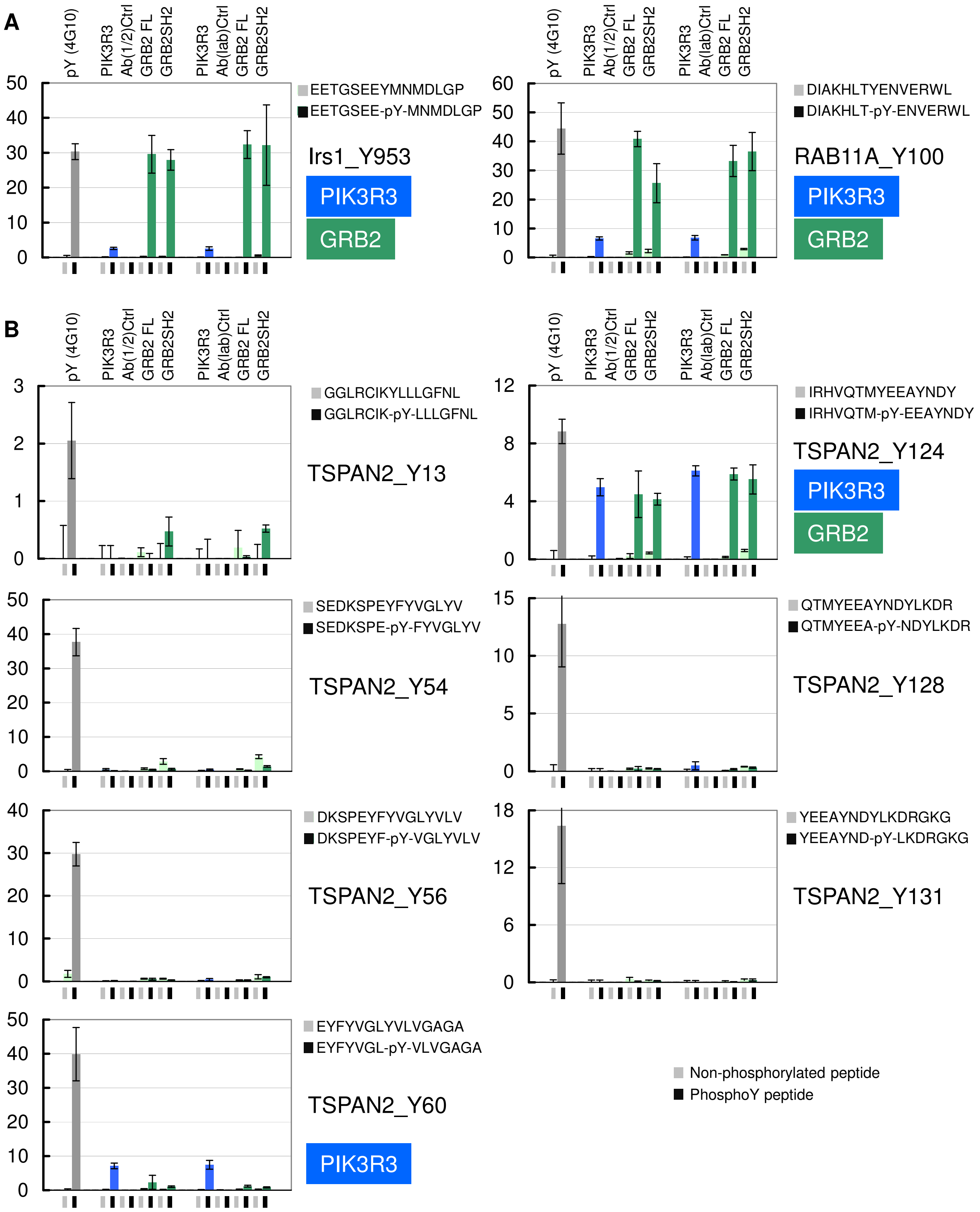

Suppl Figure S10

PCA merge

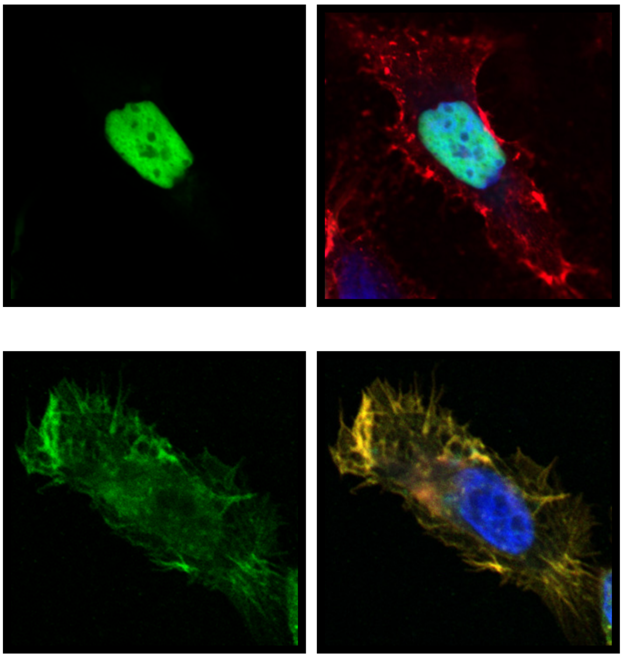

GRB2-RB1

PIK3R3-
C10orf81/PLEKHS1

Suppl. References

Auger KR, Songyang Z, Lo SH, Roberts TM, Chen LB (1996) Platelet-derived growth factor-induced formation of tensin and phosphoinositide 3-kinase complexes. *J Biol Chem* 271: 23452-23457

Beebe KD, Wang P, Arabaci G, Pei D (2000) Determination of the binding specificity of the SH2 domains of protein tyrosine phosphatase SHP-1 through the screening of a combinatorial phosphotyrosyl peptide library. *Biochemistry* 39: 13251-13260

Bisson N, James DA, Ivosev G, Tate SA, Bonner R, Taylor L, Pawson T (2011) Selected reaction monitoring mass spectrometry reveals the dynamics of signaling through the GRB2 adaptor. *Nat Biotechnol* 29: 653-658

Blume-Jensen P, Hunter T (2001) Oncogenic kinase signalling. *Nature* 411: 355-365

Bodenhofer U, Kothmeier A, Hochreiter S (2011) APCluster: an R package for affinity propagation clustering. *Bioinformatics* 27: 2463-2464

Cao H, Courchesne WE, Mastick CC (2002) A phosphotyrosine-dependent protein interaction screen reveals a role for phosphorylation of caveolin-1 on tyrosine 14: recruitment of C-terminal Src kinase. *J Biol Chem* 277: 8771-8774

Clark DD, Peterson BR (2002) Rapid detection of protein tyrosine kinase activity in recombinant yeast expressing a universal substrate. *J Proteome Res* 1: 207-209

Clark DD, Peterson BR (2003) Analysis of protein tyrosine kinase inhibitors in recombinant yeast lacking the ERG6 gene. *Chembiochem* 4: 101-107

Clark DD, Peterson BR (2005) Fluorescence-based cloning of a protein tyrosine kinase with a yeast tribrid system. *Chembiochem* 6: 1442-1448

Delahaye L, Rocchi S, Van Obberghen E (2000) Potential involvement of FRS2 in insulin signaling. *Endocrinology* 141: 621-628

Dente L, Vetriani C, Zucconi A, Pelicci G, Lanfrancone L, Pelicci PG, Cesareni G (1997) Modified phage peptide libraries as a tool to study specificity of phosphorylation and recognition of tyrosine containing peptides. *J Mol Biol* 269: 694-703

Dombrosky-Ferlan PM, Corey SJ (1997) Yeast two-hybrid in vivo association of the Src kinase Lyn with the proto-oncogene product Cbl but not with the p85 subunit of PI 3-kinase. *Oncogene* 14: 2019-2024

Ellis JH, Ashman C, Burden MN, Kilpatrick KE, Morse MA, Hamblin PA (2000) GRID: a novel Grb-2-related adapter protein that interacts with the activated T cell costimulatory receptor CD28. *J Immunol* 164: 5805-5814

Frey BJ, Dueck D (2007) Clustering by passing messages between data points. *Science* 315: 972-976

Fuller KJ, Morse MA, White JH, Dowell SJ, Sims MJ (1998) Development of a yeast trihybrid screen using stable yeast strains and regulated protein expression. *Biotechniques* 25: 85-88, 90-82

Futreal PA, Coin L, Marshall M, Down T, Hubbard T, Wooster R, Rahman N, Stratton MR (2004) A census of human cancer genes. *Nature reviews Cancer* 4: 177-183

Ingley E, Schneider JR, Payne CJ, McCarthy DJ, Harder KW, Hibbs ML, Klinken SP (2006) Csk-binding protein mediates sequential enzymatic down-regulation and degradation of Lyn in erythropoietin-stimulated cells. *J Biol Chem* 281: 31920-31929

Kamburov A, Stelzl U, Lehrach H, Herwig R (2013) The ConsensusPathDB interaction database: 2013 update. *Nucleic Acids Res* 41: D793-800

Keegan K, Cooper JA (1996) Use of the two hybrid system to detect the association of the protein-tyrosine-phosphatase, SHPTP2, with another SH2-containing protein, Grb7. *Oncogene* 12: 1537-1544

Lupher ML, Jr., Songyang Z, Shoelson SE, Cantley LC, Band H (1997) The Cbl phosphotyrosine-binding domain selects a D(N/D)XpY motif and binds to the Tyr292 negative regulatory phosphorylation site of ZAP-70. *J Biol Chem* 272: 33140-33144

Marti F, Xu CW, Selvakumar A, Brent R, Dupont B, King PD (1998) LCK-phosphorylated human killer cell-inhibitory receptors recruit and activate phosphatidylinositol 3-kinase. *Proc Natl Acad Sci U S A* 95: 11810-11815

Mayer BJ, Jackson PK, Van Etten RA, Baltimore D (1992) Point mutations in the abl SH2 domain coordinately impair phosphotyrosine binding in vitro and transforming activity in vivo. *Mol Cell Biol* 12: 609-618

Mothe I, Delahaye L, Filloux C, Pons S, White MF, Van Obberghen E (1997) Interaction of wild type and dominant-negative p55PIK regulatory subunit of phosphatidylinositol 3-kinase with insulin-like growth factor-1 signaling proteins. *Mol Endocrinol* 11: 1911-1923

Osborne MA, Dalton S, Kochan JP (1995) The yeast tribrid system--genetic detection of trans-phosphorylated ITAM-SH2-interactions. *Bio/technology* 13: 1474-1478

Poy F, Yaffe MB, Sayos J, Saxena K, Morra M, Sumegi J, Cantley LC, Terhorst C, Eck MJ (1999) Crystal structures of the XLP protein SAP reveal a class of SH2 domains with extended, phosphotyrosine-independent sequence recognition. *Mol Cell* 4: 555-561

Rocchi S, Tartare-Deckert S, Murdaca J, Holgado-Madruga M, Wong AJ, Van Obberghen E (1998) Determination of Gab1 (Grb2-associated binder-1) interaction with insulin receptor-signaling molecules. *Mol Endocrinol* 12: 914-923

Rodriguez M, Li SS, Harper JW, Songyang Z (2004) An oriented peptide array library (OPAL) strategy to study protein-protein interactions. *J Biol Chem* 279: 8802-8807

Sathish JG, Johnson KG, Fuller KJ, LeRoy FG, Meyaard L, Sims MJ, Matthews RJ (2001) Constitutive association of SHP-1 with leukocyte-associated Ig-like receptor-1 in human T cells. *J Immunol* 166: 1763-1770

Sayos J, Martin M, Chen A, Simarro M, Howie D, Morra M, Engel P, Terhorst C (2001) Cell surface receptors Ly-9 and CD84 recruit the X-linked lymphoproliferative disease gene product SAP. *Blood* 97: 3867-3874

Sayos J, Martinez-Barriocanal A, Kitzig F, Bellon T, Lopez-Botet M (2004) Recruitment of C-terminal Src kinase by the leukocyte inhibitory receptor CD85j. *Biochem Biophys Res Commun* 324: 640-647

Songyang Z, Shoelson SE, Chaudhuri M, Gish G, Pawson T, Haser WG, King F, Roberts T, Ratnofsky S, Lechleider RJ, et al. (1993) SH2 domains recognize specific phosphopeptide sequences. *Cell* 72: 767-778

Songyang Z, Shoelson SE, McGlade J, Olivier P, Pawson T, Bustelo XR, Barbacid M, Sabe H, Hanafusa H, Yi T, et al. (1994) Specific motifs recognized by the SH2 domains of Csk, 3BP2, fps/fes, GRB-2, HCP, SHC, Syk, and Vav. *Mol Cell Biol* 14: 2777-2785

Stahl N, Farruggella TJ, Boulton TG, Zhong Z, Darnell JE, Jr., Yancopoulos GD (1995) Choice of STATs and other substrates specified by modular tyrosine-based motifs in cytokine receptors. *Science* 267: 1349-1353

Stefan E, Malleshaiah MK, Breton B, Ear PH, Bachmann V, Beyermann M, Bouvier M, Michnick SW (2011) PKA regulatory subunits mediate synergy among conserved G-protein-coupled receptor cascades. *Nat Commun* 2: 598

Stynen B, Tournu H, Tavernier J, Van Dijck P (2012) Diversity in genetic in vivo methods for protein-protein interaction studies: from the yeast two-hybrid system to the mammalian split-luciferase system. *Microbiology and molecular biology reviews : MMBR* 76: 331-382

Sylvester M, Kliche S, Lange S, Geithner S, Klemm C, Schlosser A, Grossmann A, Stelzl U, Schraven B, Krause E, Freund C (2010) Adhesion and degranulation promoting adapter protein (ADAP) is a central hub for phosphotyrosine-mediated interactions in T cells. *PLoS One* 5: e11708

Tinti M, Kiemer L, Costa S, Miller ML, Sacco F, Olsen JV, Carducci M, Paoluzi S, Langone F, Workman CT, Blom N, Machida K, Thompson CM, Schutkowski M, Brunak S, Mann M, Mayer BJ, Castagnoli L, Cesareni G (2013) The SH2 domain interaction landscape. *Cell reports* 3: 1293-1305

Verbrugge A, Rijkers ES, de Ruiter T, Meyaard L (2006) Leukocyte-associated Ig-like receptor-1 has SH2 domain-containing phosphatase-independent function and recruits C-terminal Src kinase. *European journal of immunology* 36: 190-198

Volpers C, Lubinus M, Osborne MA, Kochan JP (2001) cDNA expression cloning and characterization of phosphorylation dependent protein interactors using the yeast tribrid system. *Methods Mol Biol* 124: 271-293

Warner AJ, Lopez-Dee J, Knight EL, Feramisco JR, Prigent SA (2000) The Shc-related adaptor protein, Sck, forms a complex with the vascular-endothelial-growth-factor receptor KDR in transfected cells. *Biochem J* 347: 501-509

Woodfield RJ, Hodgkin MN, Akhtar N, Morse MA, Fuller KJ, Saqib K, Thompson NT, Wakelam MJ (2001) The p85 subunit of phosphoinositide 3-kinase is associated with beta-catenin in the cadherin-based adhesion complex. *Biochem J* 360: 335-344

Xu P, Jacobs AR, Taylor SI (1999) Interaction of insulin receptor substrate 3 with insulin receptor, insulin receptor-related receptor, insulin-like growth factor-1 receptor, and downstream signaling proteins. *J Biol Chem* 274: 15262-15270

Yamada M, Suzuki K, Mizutani M, Asada A, Matozaki T, Ikeuchi T, Koizumi S, Hatanaka H (2001) Analysis of tyrosine phosphorylation-dependent protein-protein interactions in TrkB-mediated intracellular signaling using modified yeast two-hybrid system. *J Biochem* 130: 157-165
