# Supplementary figures and images for: Phospho-tyrosine dependent protein–protein interaction network
Source: Mol Syst Biol. 2015 Mar 26;11(3):0794. doi: 10.15252/msb.20145968 (PMC4380928; doi:10.15252/msb.20145968)

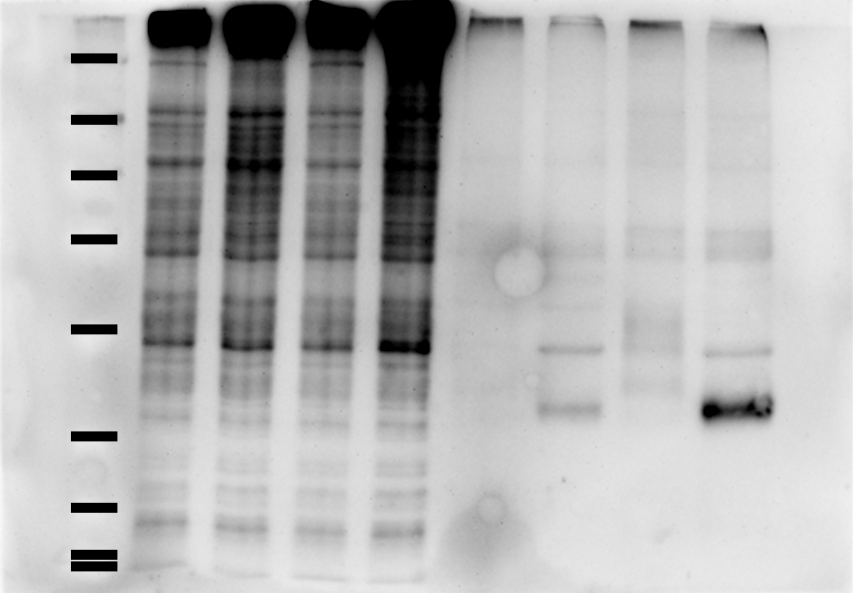

Supplement: Supplementary file 8 [file msb0011-0794-sd8.zip › Source Data for Figure 4/Fig4B-1-antiFLAG_Grb2.tif]

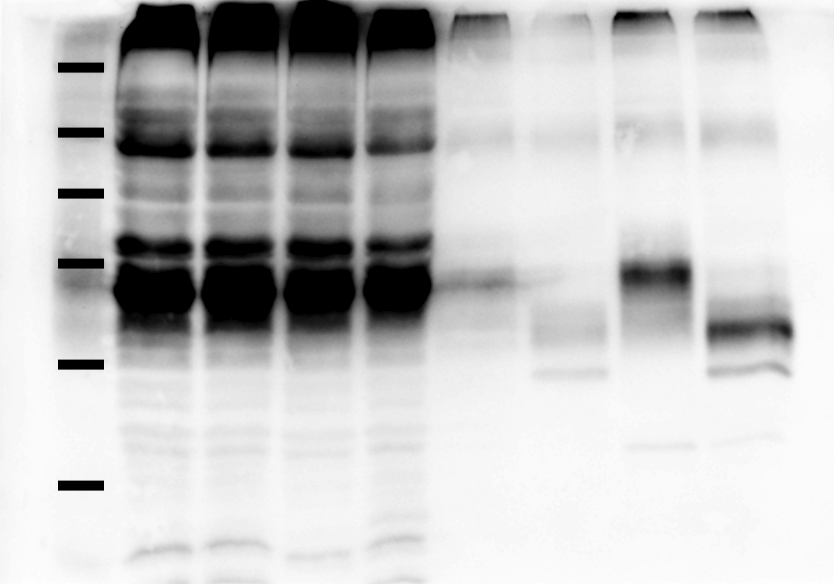

Supplement: Supplementary file 8 [file msb0011-0794-sd8.zip › Source Data for Figure 4/Fig4B-2-antiPA_PIK3R3.tif]

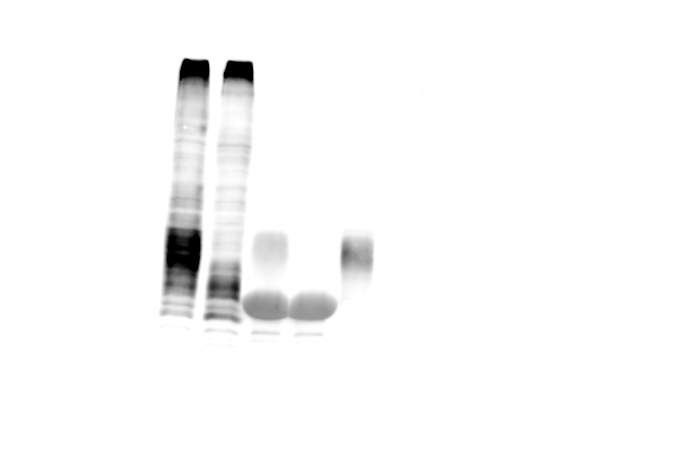

Supplement: Supplementary file 8 [file msb0011-0794-sd8.zip › Source Data for Figure 4/Fig4C-1-antiPA_PIK3R3_Grb2.tif]

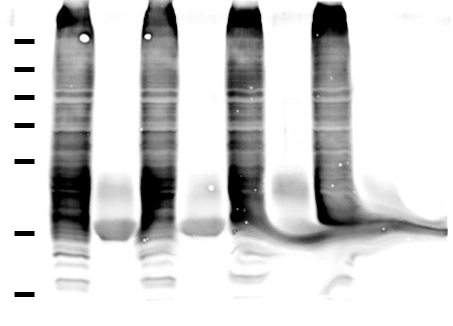

Supplement: Supplementary file 8 [file msb0011-0794-sd8.zip › Source Data for Figure 4/Fig4C-2-antiPA_Grb2_Blot1.tif]

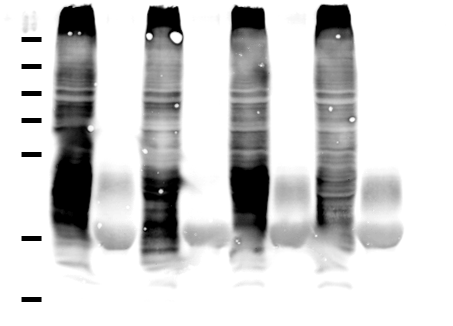

Supplement: Supplementary file 8 [file msb0011-0794-sd8.zip › Source Data for Figure 4/Fig4C-3-antiPA_Grb2_Blot2.tif]

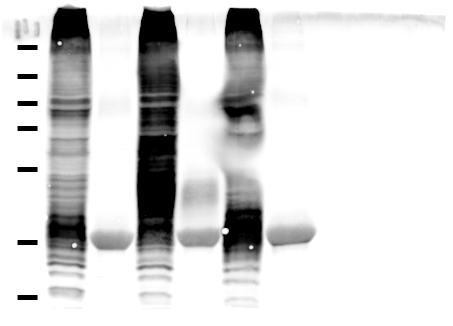

Supplement: Supplementary file 8 [file msb0011-0794-sd8.zip › Source Data for Figure 4/Fig4C-4-antiPA_Grb2_Blot3.tif]

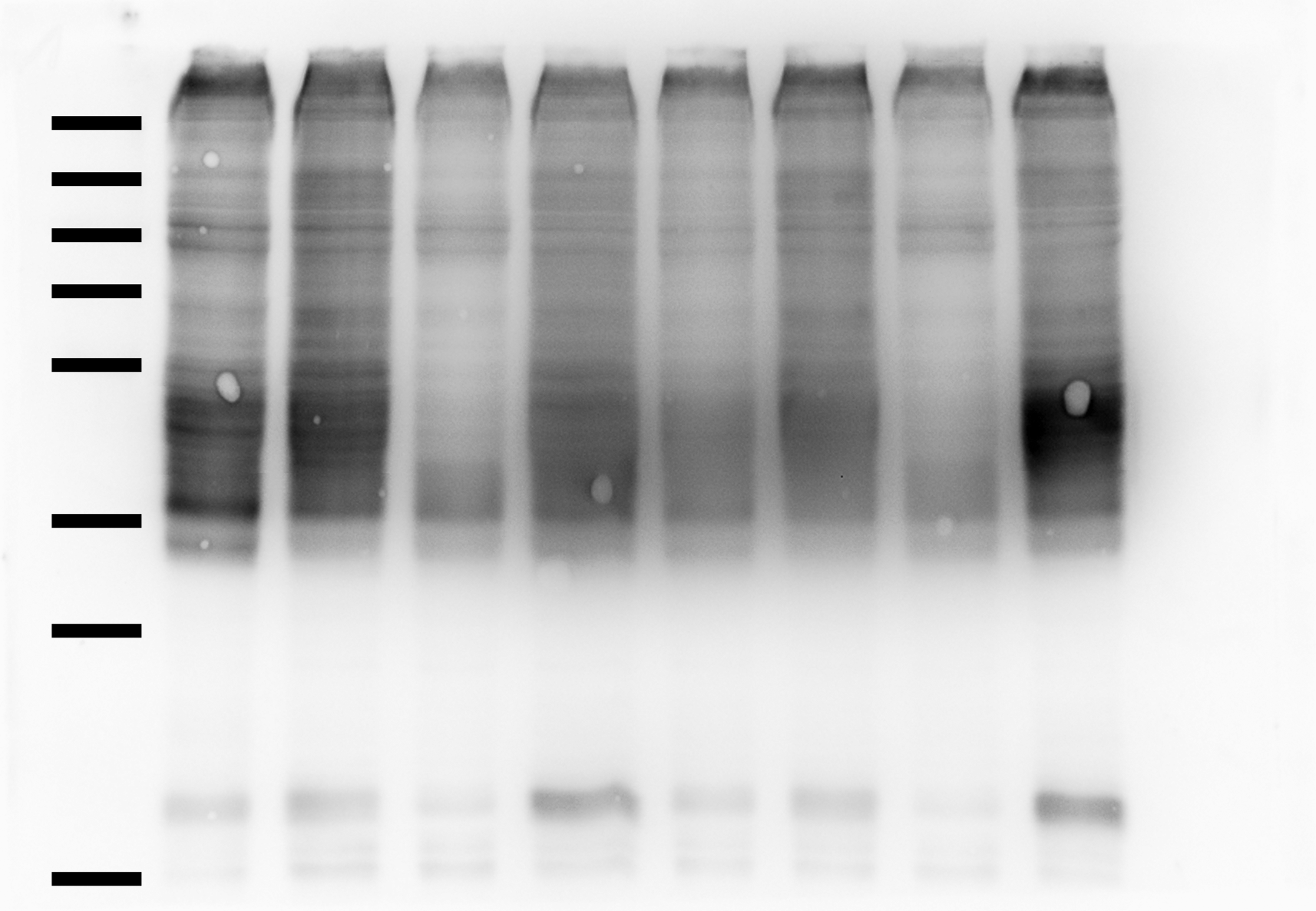

Supplement: Supplementary file 8 [file msb0011-0794-sd8.zip › Source Data for Figure 4/Fig4D-1-antiPA_InputC.tif]

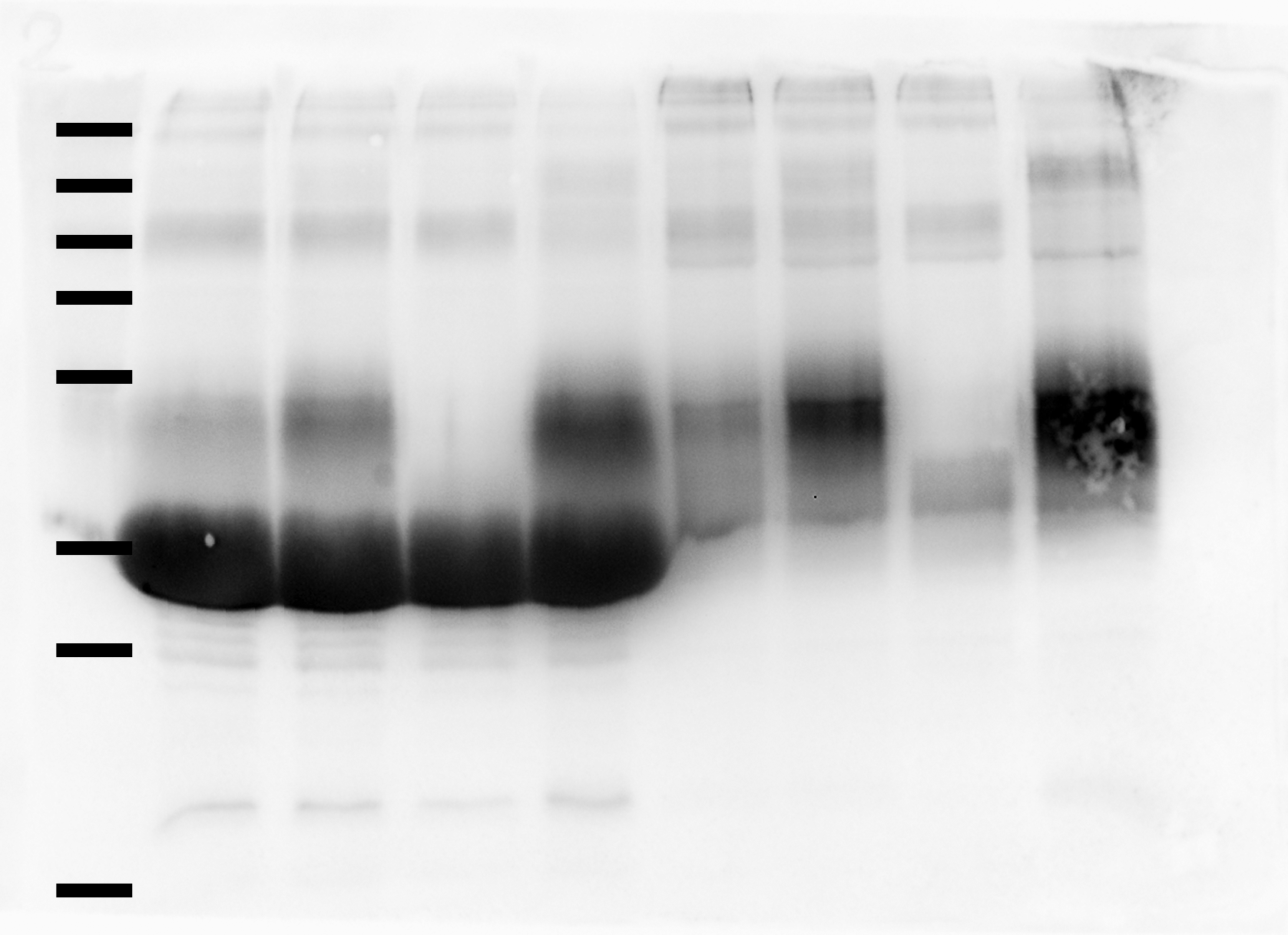

Supplement: Supplementary file 8 [file msb0011-0794-sd8.zip › Source Data for Figure 4/Fig4D-2-antiPA_outputP.tif]
