# Supplementary material for: Phospho-tyrosine dependent protein–protein interaction network
Source: Mol Syst Biol. 2015 Mar 26;11(3):0794. doi: 10.15252/msb.20145968 (PMC4380928; doi:10.15252/msb.20145968)
Supplement: Supplementary file 7 [file msb0011-0794-sd7.pdf]

Phospho-tyrosine dependent protein-protein interaction network

Arndt Grossmann, Nouhad Benlasfer, Petra Birth, Anna Hegele, Franziska Wachsmuth, Luise Apelt and Ulrich Stelzl

Corresponding author: Ulrich Stelzl, Max-Planck Institute for Molecular Genetics

Review timeline:

Submission date:	09 December 2014
Editorial Decision:	13 January 2015
Revision received:	26 January 2015
Editorial Decision:	17 February 2015
Revision received:	18 February 2015
Accepted:	19 February 2015

Editor: Maria Polychronidou

Transaction Report:

(Note: With the exception of the correction of typographical or spelling errors that could be a source of ambiguity, letters and reports are not edited. The original formatting of letters and referee reports may not be reflected in this compilation.)

1st Editorial Decision 13 January 2015

Thank you again for submitting your work to Molecular Systems Biology. We have now heard back from the three referees who agreed to evaluate your manuscript. As you will see from the reports below, the referees acknowledge that the presented approach seems interesting and they think that the identified interactions represent a potentially useful resource. However, they raise a series of concerns, which should be carefully addressed in a revision of the manuscript.

Most of the reviewers' comments refer to the need to provide further clarifications and to discuss several points in better detail. Without repeating all the points listed below, some of the more fundamental issues are the following:
- Additional evidence needs to be provided in order to better support the pY dependent TSPAN2 interactions and their potential functions.
- Further details should be provided regarding the validation of the detected interactions. While inclusion of further validation experiments is not mandatory, such data, if available, would enhance the conclusiveness of the study.

If you feel you can satisfactorily deal with these points and those listed by the referees, you may wish to submit a revised version of your manuscript. Please attach a covering letter giving details of the way in which you have handled each of the points raised by the referees. A revised manuscript will be once again subject to review and you probably understand that we can give you no guarantee at this stage that the eventual outcome will be favorable.

REFEREE COMMENTS

Reviewer #1:

The authors have executed a large-scale Y2H screening for about 188 bait ORFs representing 126 proteins with predicted phosphotyrosine recognition domains and 9 non-receptor tyrosine kinases against a prey matrix of about 17,000 ORFS. The hypothesis is that phosphorylation of the prey leads to association with the bait. From about 2 million possible interactions, they deduce with additional controls, 336 phosphorylation independent and 292 phosphorylation dependent interactions. After GO, gene neighbourhood and cancer gene census analysis, the authors focus on the protein C10orf81 (this is actually a protein commonly named Pleckstrin homology domain containing, family S member 1) that they show as a phosphorylation dependent interaction with PIK3R3 and demonstrate by mutational analysis the known Y68of this protein to be responsible for association with the C terminal SH2 domain of PIK3R3. The authors conclude that 81 % of their data represent new interactions previously unreported. In Hek293 cells, 76 of 147 pairs tested showed a positive signal by luminescence based coIP although phosphorylation dependence was minimal and concluded to be due to endogenous kinases since selected point mutations in SH2 domains of PIK3R3 and GRD2 reduced associations. The authors also focus on TSPAN2 that they showed interacted with GRB2 and PIK3R3. Mutation of 7 Y residues of TSPAN2 abolished interaction, with Y124 of TSPAN2 deduced to be of importance since it alone could restore binding in a pull down assay. The authors then express GFP-TSPAN2 in HEK293 cells and conclude that membrane protrusions and cell- cell contacts are now found but not with the Y124F mutant or 7F mutant but the phenotype is seen with the 6 F mutant restored with Y124. Through the YFP PCA assay, the authors conclude different interactions at different locations for GRB2-TSPAN2 and PIK3R3-TSPAN2.

This work is timely considering the large-scale Y2H effort recently published by the Vidal lab. To address the issues of false positives and false negatives it may be worthwhile to consider a detailed comparison of the authors' data with those of the Vidal studies. The authors may also consider adding the common names of the proteins. This would help in the easy scrutiny of false positives. For example c6orf125 is the well-known mitochondrial protein Ubiquinol-cytochrome c reductase complex assembly factor 2 and an assumed false positive as expected from these studies.
The authors may also wish to consider if the extension to TSPAN2 is also a consequence of a false positive. This is because the Y124 (and indeed all the Y residues) is expected to be unavailable to cytosolic exposed kinases since Y124 is extracellular (next to the glycosylation site) and several of the other Y residues are either extracellular or in the membrane spanning domains.
Removing this entire section (including all the expression work GFP TSPAN2 and the YFP PCA assays) may help the paper.
A detailed assessment (perhaps via the Human Protein Atlas) as to the location of the proteins and detailed comparisons to the data of Vidal's studies may enable a filtering out of false positives and an estimate of false negatives.
The paper should be encouraged for publication but only after a more through examination of all the data and less of an extension into biological validation that here may harm rather than help.

Reviewer #2:

Referee report on manuscript "Phospho-tyrosine dependent protein-protein interaction network" paper by Grossmann A. et al.

Summary

Grossmann et al. present a first large scale yeast two hybrid screen for tyrosine kinase (TK) dependent interactions between tyrosine phosphosite reader proteins containing SH2 and PTB domains and their binding partners. Among the 1223 initial interactions they found they confirmed 292 TK dependent and 336 TK independent PPIs. The majority of these interactions are novel and the authors could validate 50% of the tested PPIs using CoIP experiments in HEK293 cells. The authors conclude that the majority of the found binary tyrosine dependent interactions do not

involve known linear motif but other novel yet to be determined structural constraints. Inspecting the resulting network revealed a highly interlinked subnetwork suggesting a modular organization of the TK dependent interactome. Finally using TSPAN2 as an example they provide evidence that TK dependent interactions with specific reader proteins may be spatially constraint and may control different phenotypic outcomes following TSPAN2 overexpression.

General remarks

The authors state several times that they provide a proteome wide screen which implies a good coverage of the cellular phospho-tyrosine dependent interactome. Currently 5000 proteins have been identified to be tyrosine phosphorylated on over 30000 sites and we believe that the 292 phospho-tyrosine dependent PPIs found in their study represent a small fraction of the actual phospho-tyrosine interaction space that can be expected in human cells. Therefore the proteome scale term should be used with care. This may reflect several inherent technical limitations of the yeast system. One of the assumptions is that potential substrates among the tested 17000 candidate prey proteins are effectively phosphorylated by the nine tyrosine kinases co-expressed in yeast for which there is no evidence. It is clear that specificity of phosphorylation events depends on a number of constraints that are not met in the yeast systems (i.e. protein expression level, complex formation of substrates as well as kinases with other human proteins, human specific PTMs of kinases and substrates, compartmentalization etc.). The same is true for the interaction between reader and substrates. None of these technical limitations are discussed. We certainly view the presented binary interaction data as important but far away from being proteome wide when it comes to the situation in human cells. Nevertheless the presented study is the largest of its kind and will add important new information for modeling intracellular information processing on the basis of binary tyrosine phosphorylation dependent signaling networks. Besides basic signaling research we believe that the presented work will attract also biomedical scientists given the central role of altered phospho-tyrosine signaling systems in pathophysiological conditions.
I appreciate that the authors made significant efforts to validate their interaction data in mammalian cells which results in a robust set of novel candidate phospho-tyrosine interactions which can be further studied in more detail by the signaling community in human cells using methods that go beyond the scope of the presented systematic study. The notion that many of the identified interactions do not rely on known linear motifs is interesting and may encourage the quest for novel structural constraints for reading the phospho-tyrosine code.

Major points

1) At current state the main text and methods sections are not sufficient to fully understand how the final list of interactions has been assembled. For example: page 5 "We also tested a subset of interacting pairs with kinase-deficient version of the 9 kinases, rendered inactive by an arginine to methionine mutation in the ATP binding site" The subset is not described. Why did authors not retest instead the entire set of 1223 PPI with the kinase dead mutants? Furthermore "In this assay we additionally examined 37 interactions with a comprehensive set of 31 non-receptor tyrosine kinases." On what basis were these 37 interactions and the 31 kinases selected? The systematic character of the study is compromised when systematic screens and "subset" screens are pooled together. A clear flow diagram should be provided in Figure 1 with all essential details and numbers that fully explain where the 292 phospho-tyrosine dependent PPI are finally coming from the screen.

2) Page 5 bottom paragraph: "we report a large data resource comprising 336 independent and 292 phosphorylation-dependent protein-protein
interactions". The reoccurring term "phosphorylation dependent" should be clearly defined: do the authors mean kinase dependent or kinase activity dependent to define the set of 292 interactions? Since actual phosphorylation events are not measured the term "phosphorylation dependent" should be used very carefully.

3) Is there a fraction among the kinase dependent interactions that do not require catalytic activity but rather reflect trimeric complex formation between reader, kinase and a kinase interacting protein?

4) Fig. S3: It is surprising to see frequent cases were a phospho-tyrosine dependent PPI is controlled by so many different cellular TKs: i.e. IRS-Numb or GRB2-C10orf81 are controlled by more than

© European Molecular Biology Organization

10 TKs corresponding to about 1/3 of TK's tested. Is there any correlation between this observed redundancy to the abundance or activity of the human kinase expressed in yeast? What is the conclusion from the propagation clustering? Do the authors have evidence that the redundancy observed in their screen is also reflected in vivo in human cells or rather reflects compromised specificity in tyrosine phosphorylation in their yeast system?

5) Page 6: The authors state on page 6 that the substrates are enriched for cancer proteins but do not provide details in their supplement e.g. which and what fraction of their substrates was found in the cancer census set. Did they include kinase dependent prey proteins or all interacting proteins?

6) Validation experiments by CoIP presented in Figure 3: it is unclear whether all 147 PPI were tested in the presence/absence of Fyn and ABL2 (as indicated in the method section) or just a subset. Table S7 does also not indicate which kinase was cotransfected. One simple way to address the phospho-tyrosine dependency could involve inhibition of tyrosine phosphatases by vanadate treatment, which would boost the claimed phospho-tyrosine dependent interactions in their CoIP experiments.

7) Legend to Figure 4B is quite poor: please explain what has been done and what is shown in 4B: what represents "C", what is "P"? What is the anti Flag WB detecting in 4B? From the method section it appears that Abl2 was coexpressed with TSPAN. Is the presence of Abl2 required for PA-TSPAN -GST pull downs to work?

8) No experimental data for in vivo Y124 phosphorylation is provided to support the proposed model for the phospho-tyrosine controled TSPAN2 interactions

9) A WB should be included to control for the TSPAN2 levels in the IF experiments shown in 4F and G.

10) Page 11 the authors stated "Our data support a possible function of phospho-tyrosine 124 mediated TSPAN2 interactions connected to cell migration, adhesion and spreading and thus provides a mechanistic entry point to unravel the effects of elevated TSPAN2 levels in cancer such as lung adenocarinomas". While membrane protrusions have been observed upon TSPAN2 overexpression, the authors do not provide any data that cell migration and adhesion are indeed affected by TSPAN2. Either they tone down their claim or they should provide additional experimental evidence that directly establish a role for TSPAN2 in migration and adhesion.

11) Would be great if the authors could provide evidence that TSPAN2 phenotypes observed with wt and mutant proteins correlate with their interactions with endogenous Grb2.

12) On page 12 the authors conclude that "The more detailed characterization of the TSPAN2-GRB2 and TSPAN2-PIK3R3 interactions exemplarily demonstrates how PPIs are dynamically and spatially constrained". Whereas evidence is provided (based on PCA overexpression experiments) that these two interactions are spatially separated, there is no evidence provided how these PPI are dynamically constrained in the presented data.

Minor points

1) The reading of the text could improve by structuring the result section with headings

2) A graphical legend within the Figure 2A which explains the nature of edges and nodes should be provided

3) Legend to Figure 3C unclear "Selection of PIK3R3 and GRB2 co-IP results from HEK293 cells (serum conditions)": What do the dark and bright green bars represent? Where are the GRB2 co-IP results?

4) The PA-tag should be explained once. I assume it is protein A but it is nowhere explained in the text.

5) Page 8 The authors state "In the majority of the cases the C-terminal SH2-domain mutation (R383K) had a stronger diminishing effect on binding, however when both SH2-domains were mutated simultaneously, pY-dependent binding was typically decreased most strongly (Figure 3D)." however Figure 3D does not show the results obtained for R383K of PIK3R3 but only for the double mutant.

6) Please clarify in the method section whether the Co-IP validation experiments were performed in the presence of ABL2 or Fyn or in their absence - this is not clearly stated in the text.

7) The authors may add in the last paragraph on page 3 of the introduction that besides linear motifs, complex formation with other human proteins, compartmentalization and additional PTMs may add to the specificity of P-Y recognition.

8) Page 7/8: validation of literature data is not clear: what do the authors exactly mean with: 143 literature-curated interactions in the pY-reader - pY-reader search space? Please explain.

9) Fig.S2: Figure as well as legend should contain more information: what kinase construct and controls were used in the retesting? Otherwise the presented data is hard to interpret.

Reviewer #3:

In this manuscript the authors describe a new approach to determine human protein-protein interactions that are dependent on protein tyrosine phosphorylation. This was achieved with a modified yeast-two-hybrid (Y2H) protocol using as bait full proteins containing domain families that are known to be able to interact with phosphotyrosines. The dependence on phosphorylation is measured by testing the same interactions in the presence or absence of protein tyrosine kinases. The experimental set-up allowed the authors to determine 292 interactions that appear to depend on the presence of tyrosine kinases and an additional 336 interactions that are independent of the presence of the tyrosine kinases.

As expected for any novel experimental approach the authors have tried to provide extensive testing of the accuracy of the identified interactions. It is this aspect that is somewhat disappointing. To the authors credit they have tried many different approaches to corroborate the phosphorylation dependence of the identified interactions. They have: compared the identified interactions with previous knowledge from the literature; tested the over-representation of tyrosine binding motifs and previously known tyrosine phosphosites; re-tested the same interactions with co-IPs in the presence and absence of expressed kinases as well as with mutated versions of the binding domains. Many of the tests fall short of being overwhelmingly positive. However, the approach is very creative and novel. This is the first large-scale approach to identify in-vivo phosphorylation dependent protein interactions and can, in principle, be extended to other types of PTMs. The most related approach would be conditional dependent affinity-purification mass-spectrometry (AP-MS) but, as was the case for "static" protein interactions, conditional AP-MS and this Y2H approach are expected to provide different and complementary information. The authors have additionally provided more extensive validation for a specific interaction (TSPAN2 with GRB2/PIK3R3) which exemplifies how this resource may be used in the future by researchers interested in cell signalling. Given the novelty of the approach and the potential usefulness of the determined interactions I believe this work would be of interest to large audience.

Major concerns:
1 - I have only one major concern. As mentioned above, the tests performed to validate the phosphorylation dependence of the interactions are not overwhelmingly positive. For example the overlap with interactions known from the literature is very small (~6%) even if likely to be significant. The interactions were found to be fairly reproducible (50%) in co-IPs done in human cells but almost none of these were found to be dependent on the co-expression of a tyrosine kinase. As pointed out by the authors we might expect that the baseline tyrosine kinase activity of the cells

© European Molecular Biology Organization

used might make the phosphorylation dependence very hard to measure with these assays. Finally, for a small set of 13 interactions the authors show that impairing the phospho-tyrosine binding domains has a very significant effect on the interactions which indirectly implies that they are phosphorylation dependent. So although the authors have done very extensive computational and experimental tests it remains hard to evaluate to what extent the 292 interactions are indeed dependent on tyrosine phosphorylation. Performing detailed studies of additional interactions is clearly beyond the scope of this article and the authors have already done extensive validation work. It is possible that the only plausible resolution for this concern will be to improve the interpretation and discussion of these results being cautious about the claims.

Given that the mutations of the phosphotyrosine binding domains provided the strongest validation the authors may at least try to be clearer about this validation assay. Were the 13 interactions the only ones tested in the way ? This implies a 100% dependence on the phosphotyrosine domain families which is not even fully expected since this was done in the context of full proteins. If this validation assay could be extended to additional domain(s)/interactions it would provide additional assurance of the phospho dependence of the interaction network provided here. However, I would not suggest this is a requirement for publication.

Minor concerns:
1 - How exactly was the kinase dependence scored ? If I understood correctly, all 1223 robust Y2H interactions were re-tested with 5 biological replicates of empty vector and with a panel of tyrosine kinases (as in supplementary figure 3). How did the authors define the kinase dependence and/or kinase specificity ? Was this defined based on a quantitative model from the colony growth/sizes ? There are several interactions (e.g. YES1-OLIG1, PIK3R3-PELO, GRB2-LMX1A, GRB2-PPARA) that are listed as phospho dependent but in supplementary figure 3 the empty vector colonies appear to be growing well. Perhaps the authors could provide a quantitative metric for each interaction describing the kinase dependence and/or kinase specificity of the interaction. For example if all colony growth were quantified using image analysis then the distribution of scores for the empty vectors in each interaction could be used as a the null model to score each of the kinases. Ranking phospho dependence in this way might facilitate the re-use of this dataset.

2 - The kinases that drive the phosphorylation dependence are very often ABL2 (80% of interactions), FYN (53%) or TNK1 (39%). I was not expecting that a single kinase (i.e. ABL2) would explain so many of the phosphorylation dependent interactions. Do the proportions of phosphorylation dependent interactions for each kinase correspond also to the proportion of known protein-protein interactions that these kinases are known to take part in ? In other words, are kinases that are very often associated with the phosphorylation dependence also have many known protein-protein interactions in human and vice-versa ? If this is not the case, is there another reason for this skewness ? Are the top kinases more promiscuous ? I noticed that ABL2 is described to have a nuclear localization signal. Maybe the putative cellular localizations of the expressed kinases has an impact of their activity in yeast ?

3 - There are several assumptions and limitations that could be better discussed. For example, several of the proteins that have phosphotyrosine domains are themselves tyrosine kinases. I assume that these kinases were not mutated before using them as baits. The intrinsic kinase activity of these could make the kinase dependence hard to score. Conversely, many tyrosine kinases exist in an inactive state. The authors essentially look for positive signals but it is possible that several of the over-expressed kinases might not have enough basal activity to phosphorylate the target proteins. On the other hand it is probably good that the the authors did not use activated kinases since it is known that addition of active tyrosine kinases to yeast cause fitness defects. I don't think the authors mentioned that S. cerevisiae does not have tyrosine kinases which is an obvious advantage to performing these studies in yeast. I think several of these aspects should be better explained and discussed in the manuscript.

4 - Several figures could benefit from additional labels. In most cases where color is used to convey information the labels are not available in the figures and one has to read the figure legends to decode it. Examples include Figure 2A, 2B, 2C and 3C.

1st Revision - authors' response 26 January 2015

Point by point reply

Reviewer #1:

The authors have executed a large-scale Y2H screening for about 188 bait ORFs representing 126 proteins with predicted phosphotyrosine recognition domains and 9 non-receptor tyrosine kinases against a prey matrix of about 17,000 ORFS. The hypothesis is that phosphorylation of the prey leads to association with the bait. From about 2 million possible interactions, they deduce with additional controls, 336 phosphorylation independent and 292 phosphorylation dependent interactions. After GO, gene neighbourhood and cancer gene census analysis, the authors focus on the protein C10orf81 (this is actually a protein commonly named Pleckstrin homology domain containing, family S member 1) that they show as a phosphorylation dependent interaction with PIK3R3 and demonstrate by mutational analysis the known Y68of this protein to be responsible for association with the C terminal SH2 domain of PIK3R3. The authors conclude that 81 % of their data represent new interactions previously unreported. In Hek293 cells, 76 of 147 pairs tested showed a positive signal by luminescence based coIP although phosphorylation dependence was minimal and concluded to be due to endogenous kinases since selected point mutations in SH2 domains of PIK3R3 and GRD2 reduced associations. The authors also focus on TSPAN2 that they showed interacted with GRB2 and PIK3R3. Mutation of 7 Y residues of TSPAN2 abolished interaction, with Y124 of TSPAN2 deduced to be of importance since it alone could restore binding in a pull down assay. The authors then express GFP-TSPAN2 in HEK293 cells and conclude that membrane protrusions and cell- cell contacts are now found but not with the Y124F mutant or 7F mutant but the phenotype is seen with the 6 F mutant restored with Y124. Through the YFP PCA assay, the authors conclude different interactions at different locations for GRB2-TSPAN2 and PIK3R3-TSPAN2.

This work is timely considering the large-scale Y2H effort recently published by the Vidal lab. To address the issues of false positives and false negatives it may be worthwhile to consider a detailed comparison of the authors' data with those of the Vidal studies.

We have looked at the Vidal HI-2012 data for quite some time and we are very pleased to see it published in Cell recently. As demonstrated by the Vidal group in the paper (and witnessed by others including us) the data are of very high quality. However the data are extremely sparse. In other words the data only touch on the interactions that should be found in the huge search space that was screened - due to very low sensitivity. Of course we can simply query the interacting baits in this set: 49 of our baits are included in the data. Reassuringly, none of our pY-dependent PPI is contained in the Vidal data set. On the other hand, only three interactions of our independent data overlap in HI-2012. We think that no solid conclusions can be drawn from this comparison because of the low numbers.

The authors may also consider adding the common names of the proteins. This would help in the easy scrutiny of false positives. For example c6orf125 is the well-known mitochondrial protein Ubiquinol- cytochrome c reductase complex assembly factor 2 and an assumed false positive as expected from these studies.

We agree that often it is much more practical for colleagues who are very familiar with certain proteins to use the common names. But this is difficult in large scale approaches and bears the big danger of inconsistencies. Our reference is NCBI ENTREZ GeneID, which we think is currently one of the most stable identifier for human genes/proteins. The names used throughout the study are ENTREZ Gene Symbol, which admittedly undergo changes at a much faster pace (as happened e.g. for c6orf125). We have included alternative/common names for all proteins mentioned in the main and suppl. text; in particular we refer to C10orf81/PLEKHS1 throughout the manuscript now.

Two side notes on this point.

i) We have clearly indicated - by Refseq accession - which variant of C10orf81/PLEKHS1 we were considering (Figure 2D, NP_001180363). This variant, is currently the main isoform at NCBI,

however does not contain the predicted PLEKH domain.

ii) We agree that the biological context of the interactions involving C6orf125/UQCC2 remain unclear (putative biological false positive), however we find no evidence to suggest this interaction is not physically possible. C6orf125/UQCC2 has three reported pYs in phosphosite plus (reliably or not?).

The authors may also wish to consider if the extension to TSPAN2 is also a consequence of a false positive. This is because the Y124 (and indeed all the Y residues) is expected to be unavailable to cytosolic exposed kinases since Y124 is extracellular (next to the glycosylation site) and several of the other Y residues are either extracellular or in the membrane spanning domains. Removing this entire section (including all the expression work GFP TSPAN2 and the YFP PCA assays) may help the paper.

We agree with the comment of the reviewer in that there are open points with respect to the identified TSPAN2 interactions. On the other hand we want to review the data situation again:

*) The TSPAN2/GRB2 and PIK3R3 interactions were extensively tested in pull downs:

phosphatase treatment abolished the PPI

Y124F point mutation abolished the PPI

Y124 back mutation in on otherwise YtoF mutant protein restored interaction

*) On a peptide array strong relative binding was observed with the phospho-15mer peptide but not with the non-phosphorylated control. As positive controls, pYxN peptides similarly bound our purified
GRB2 and GRB2-SH2 domain proteins on the array. Please note the E at the (+2) position does not preclude binding as the ENLSKRpYEEIYLK (RB1, Y321) was reported to bind to GRB2-SH2 domain by Tinti et al. Cell reports (2013). However, the exact binding mode is elusive.

*) wt TSPAN2 protein localization matches exactly the expectation of a TSPAN family member. Please note that in Human Protein Atlas (Subcell atlas) TSPAN2 localization (nuclear) is very likely wrong.

*) The overexpression phenotype is strongly reminiscent of cellular phenotypes with other TSPAN family members, in particular the spreading and formation of protrusions.

*) It is very hard to ignore the phenotype in particular with the specific localization of TSPAN2 in the cellular protrusions. We have carefully quantified the phenotype, controlling e.g. for transfection efficiency. The effect / reversal of the phenotype in case of the Y124 mutant versions is striking.

*) We do not show *in vivo* phosphorylation of TSPAN2-Y124. However, several TSPANS are reported to be phosphorylated on Ys. Maybe most reliable and most relevant is the phosphorylation of Y122 in TSPAN8 (A. Moritz, Y. Li, A. Guo, J. Villén, Y. Wang, J. MacNeill, J. Kornhauser, K. Sprott, J. Zhou, A. Possemato, J. M. Ren, P. Hornbeck, L. C. Cantley, S. P. Gygi, J. Rush, M. J. Comb, Akt–RSK–S6 Kinase Signaling Networks Activated by Oncogenic Receptor Tyrosine Kinases. Sci. Signal. 3, ra64 (2010).) This Y aligns exactly at the right region with Y124 of TSPAN2.

*) We do not claim that cytosolic kinases are likely to phosphorylate TSPAN2. We can only speculate at this point but interaction with RTKs could provide a lead as does the recent report of extra cellular tyrosine kinase activity (Bordoli, Cell 2014).

In summary, we agree that there are open questions with regards to the biology of these interactions. In the revised version of the manuscript, we have included a discussion section (page 14, last paragraph ff.). This means we now clearly separate our results from a more thorough discussion about the TSPAN2 interactions including the extra literature data mentioned above. We also state that “we cannot rule out

indirect effects of the Y124F mutation, e.g. on TSPAN2 glycosylation, related to the observed cellular phenotype" (page 15, first paragraph).

A detailed assessment (perhaps via the Human Protein Atlas) as to the location of the proteins and detailed comparisons to the data of Vidal's studies may enable a filtering out of false positives and an estimate of false negatives.

The paper should be encouraged for publication but only after a more through examination of all the data and less of an extension into biological validation that here may harm rather than help.

Reviewer #2:

Summary

Grossmann et al. present a first large scale yeast two hybrid screen for tyrosine kinase (TK) dependent interactions between tyrosine phosphosite reader proteins containing SH2 and PTB domains and their binding partners. Among the 1223 initial interactions they found they confirmed 292 TK dependent and 336 TK independent PPIs. The majority of these interactions are novel and the authors could validate 50% of the tested PPIs using CoIP experiments in HEK293 cells. The authors conclude that the majority of the found binary tyrosine dependent interactions do not involve known linear motif but other novel yet to be determined structural constraints. Inspecting the resulting network revealed a highly interlinked subnetwork suggesting a modular organization of the TK dependent interactome. Finally using TSPAN2 as an example they provide evidence that TK dependent interactions with specific reader proteins may be spatially constraint and may control different phenotypic outcomes following TSPAN2 overexpression.

General remarks

The authors state several times that they provide a proteome wide screen which implies a good coverage of the cellular phospho-tyrosine dependent interactome. Currently 5000 proteins have been
identified to be tyrosine phosphorylated on over 30000 sites and we believe that the 292 phosphotyrosine dependent PPIs found in their study represent a small fraction of the actual phospho-tyrosine interaction space that can be expected in human cells.

Therefore the proteome scale term should be used with care. This may reflect several inherent technical limitations of the yeast system. One of the assumptions is that potential substrates among the tested 17000 candidate prey proteins are effectively phosphorylated by the nine tyrosine kinases co-expressed in yeast for which there is no evidence. It is clear that specificity of phosphorylation events depends on a number of constraints that are not met in the yeast systems (i.e. protein expression level, complex formation of substrates as well as kinases with other human proteins, human specific PTMs of kinases and substrates, compartmentalization etc.). The same is true for the interaction between reader and substrates. None of these technical limitations are discussed.

We have included a discussion section where we in detail mention limitations of our approach. We use the term proteome-scale only in conjunction with our prey matrix (covering a very good fraction of the proteome). We toned down and do not claim comprehensiveness of the interaction data, rather our analysis against the literature indicated that we cover only a small fraction of the pY-PPIs. We discuss the technical limitations of the approach (page 11, last paragraph ff.) and potential false negative results in the manuscript (page 13 last to page 14 first paragraph).

We certainly view the presented binary interaction data as important but far away from being proteome wide when it comes to the situation in human cells. Nevertheless the presented study is the largest of its kind and will add important new information for modeling intracellular information processing on the basis of binary tyrosine phosphorylation dependent signaling networks. Besides basic signaling research we believe that the presented work will attract also biomedical scientists given the central role of altered phospho-tyrosine signaling systems in pathophysiological conditions.

I appreciate that the authors made significant efforts to validate their interaction data in mammalian cells which results in a robust set of novel candidate phospho-tyrosine interactions which can be further studied in more detail by the signaling community in human cells using methods that go beyond the scope of the presented systematic study. The notion that many of the identified interactions do not rely on known linear motifs is interesting and may encourage the quest for novel structural constraints for reading the phospho-tyrosine code.

We thank the reviewer for the fair overall assessment of the study and for clearly recognizing the major conclusions drawn in the manuscript.

Major points

1) At current state the main text and methods sections are not sufficient to fully understand how the final list of interactions has been assembled. For example: page 5 "We also tested a subset of interacting pairs with kinase-deficient version of the 9 kinases, rendered inactive by an arginine to methionine mutation in the ATP binding site" The subset is not described.

As will be also clearer now from the flow chart (Suppl. Figure S2): All 292 interactions are the result of the pY-Y2H screening pipeline. The example mentioned here is a selected, representative subset for validation purposes trying to cover several hypothesis (a practical decision). We included known pairs (IRS1-PIK3R3, FYB-LCP2), two or more PPIs from the same prey (OLIG1 interactions) or added interactions deemed interesting. In any case the full experimental data of all 37 pairs present in the subset are shown in Suppl. Figure S4. We have specified this on page 6.

Why did authors not retest instead the entire set of 1223 PPI with the kinase dead mutants?

This is for technical reasons: The retest is designed to look for positive signals and was controlled with 12 empty vector control spots per interaction (Suppl. Figure S3). It uses fresh strains in screening configuration. The assay where we included the kinase-dead mutants is of different design, particularly suited to control for the absence of interactions. We use yeast strains that are co-transformed with the interacting bait and prey plasmid and mate this strain against 48 strains carrying the kinases (wild type and kinase death respectively). We then pipetted defined amounts of the diploid yeast onto selective agar, to mention just one more technical difference (for a detailed description of the assay see legend Suppl. Figure S4). This is very laborious but necessary to directly compare wild-type kinase with kinase-dead version. Please note that we did not obtain any comparable signal with the kinase-dead version for the tested interactions (see also answer to point 3 below).

Furthermore "In this assay we additionally examined 37 interactions with a comprehensive set of 31 non-receptor tyrosine kinases." On what basis were these 37 interactions and the 31 kinases selected?

Please see answer above for the 37 interactions. The kinases are 31 of 32 human non-receptor TKs. We simply failed to get a correct JAK1 clone to be 100% complete. This is now clearly stated in the legend to Suppl. Figure S4.

The systematic character of the study is compromised when systematic screens and "subset" screens are pooled together. A clear flow diagram should be provided in Figure 1 with all essential details and numbers that fully explain where the 292 phospho-tyrosine dependent PPI are finally coming from the screen.

This is an excellent suggestion. A flow diagram is included as Suppl. Figure S2, which ought to clearly point out that all 292 interactions are derived from the systematic pY-Y2H matrix screening.

2) Page 5 bottom paragraph: "we report a large data resource comprising 336 independent and 292 phosphorylation-dependent protein-protein interactions". The reoccurring term "phosphorylation dependent" should be clearly defined: do the authors mean kinase dependent or kinase activity dependent to define the set of 292 interactions? Since actual phosphorylation events are not measured the term "phosphorylation dependent" should be used very carefully.

We agree with the reviewer and point towards the important distinction between kinase-dependent and phosphorylation-dependent interactions twice in the main text (page 6, last paragraph and page 13 middle paragraph).

The wording phosphorylation-dependent is heavily inferred from the requirement for kinase activity in the pY-Y2H assays as well as from using YtoF mutations in substrates or RtoK mutations in SH2 domains in the validation experiments. See also answer to point 3 below.

3) Is there a fraction among the kinase dependent interactions that do not require catalytic activity but rather reflect trimeric complex formation between reader, kinase and a kinase interacting protein?

We have carefully tested the requirement for kinase activity for 37 pY-PPIs in parallel with the nine kinase dead mutations of the kinases used in the pY-Y2H screen. None of the kinase-dead mutants promoted the interaction. From this we conclude that "the identified kinase-dependent interactions are likely phosphorylation-dependent in the Y2H assay system".

From our Y2H experience (which is substantial) we can say that it is very hard to detect bridging interactions. All our attempts to specifically screen for trimeric interactions with the system used here failed in the past. Also there are probably only very few papers that actually ever reported a triple interaction with a Y2H system. The one I specifically know of is Pause et al. (PNAS 1999) who assayed the VHL-cullin2 interaction that is bridged by elongin B and elongin C.

4) Fig. S3: It is surprising to see frequent cases were a phospho-tyrosine dependent PPI is controlled by so many different cellular TKs: i.e. IRS-Numb or GRB2-C10orf81 are controlled by more than 10 TKs corresponding to about 1/3 of TK's tested. Is there any correlation between this observed redundancy to the abundance or activity of the human kinase expressed in yeast? What is the conclusion from the propagation clustering? Do the authors have evidence that the redundancy observed in their screen is also reflected in vivo in human cells or rather reflects compromised specificity in tyrosine phosphorylation in their yeast system?

A very good point which we elaborated on in the new discussion part (page 12 last to page 13 first paragraph): Whether or not a pY-PPI is promoted by a kinase depends on several parameters in our assay: including i) protein expression levels, ii) kinase activity, iii) kinase specificity and iv) interaction specificity. The parameters vary greatly between kinases and interacting pairs. Furthermore tyrosine kinases are not subject to regulation in the yeast system. So we cannot make conclusions about the kinases for the in vivo situation in human. However the system is "background free" and thus unambiguously identifies kinase candidates which can phosphorylate human proteins thereby promoting pY-dependent interactions.

The analysis of the interaction patterns using propagation clustering is not too revealing, the patterns are complex due to the many parameters in the assay, they are neither subsets nor are attributed to specific kinase activity. In only a few cases the clustering revealed that the kinase pattern can be explained by one of the interaction partners (see legend to Suppl. Figure S4). As a side note to this: this is the reason why we did not expand using this very laborious assay (see also point 1).

5) Page 6: The authors state on page 6 that the substrates are enriched for cancer proteins but do not provide details in their supplement e.g. which and what fraction of their substrates was found in the cancer census set. Did they include kinase dependent prey proteins or all interacting proteins?

This point is addressed with a new Suppl. Table S5 presenting the analysis including the fraction and identity of the cancer proteins. The analysis was performed on both sets, which is now also indicated in the main text (page 7, end of first paragraph).

6) Validation experiments by CoIP presented in Figure 3: it is unclear whether all 147 PPI were tested in the presence/absence of Fyn and ABL2 (as indicated in the method section) or just a subset. Table S7 does also not indicate which kinase was cotransfected. One simple way to address the phospho-tyrosine dependency could involve inhibition of tyrosine phosphatases by vanadate treatment, which would boost the claimed phospho-tyrosine dependent interactions in their CoIP experiments.

This is clarified in the revision: in the co-IP we used a HEK293 cell line stably expressing ABL2 including phosphatase inhibitory cocktail in the lysis buffer (material methods, page 17). However, as reported induction and serum starving did not change the result except for the cases shown in Figure 3B. This is clarified now on page 26 and a legend was added in panel Figure 3B.

7) Legend to Figure 4B is quite poor: please explain what has been done and what is shown in 4B: what represents "C", what is "P"? What is the anti Flag WB detecting in 4B? From the method section it appears that Abl2 was co-expressed with TSPAN. Is the presence of Abl2 required for PA-TSPAN -GST pull downs to work?

Legend is revised to provide more clarity! In the experiments either ABL2 was induced to raise pY-levels or unspecific H2O2 treatment was performed (stated on page 18 last paragraph). Both treatments give the same result in the pull-downs, which indicates that we need to increase pY-activity to get a good pulldown but not necessarily by ABL2 induction.

8) No experimental data for in vivo Y124 phosphorylation is provided to support the proposed model for the phospho-tyrosine controled TSPAN2 interactions.

Yes, this is correct and we refer to this point now specifically in the discussion (page 15, first paragaph). We present indirect support from the literature. Several TSPANS are reported to be phosphorylated on Ys. Maybe most reliable and most relevant is the phosphorylation of Y122 in TSPAN8 (A. Moritz, Y. Li, A. Guo, J. Villén, Y. Wang, J. MacNeill, J. Kornhauser, K. Sprott, J. Zhou, A. Possemato, J. M. Ren, P. Hornbeck, L. C. Cantley, S. P. Gygi, J. Rush, M. J. Comb, Akt–RSK–S6 Kinase Signaling Networks Activated by Oncogenic Receptor Tyrosine Kinases. Sci. Signal. 3, ra64 (2010).) This Y aligns exactly at the rightregion with Y124 of TSPAN2.

9) A WB should be included to control for the TSPAN2 levels in the IF experiments shown in 4F and G.

YFP-TSPAN2 is hard to detect via WB, in particular under the conditions of the IF experiment. We can monitor expression of the YFP-TSPAN2 proteins via WB in a scale up, which would then not serve as an appropriate control for the IF experiment.

Please note that images were taken in parallel with exactly the same microscopy settings and that the transfection efficiency was carefully assessed. Therefore, fluorescence intensity and transfection efficiency are quite comparable within panel F and G: Transfected YFP positive cells: G: wt 83%; Y124F 68%, "124Y only" 73%, Y5-7F 78%; F: PCA GRB2-TSPAN2: 67%, PIK3R3-TSPAN2: 68%. The PCA fluorescence signal is generally weaker than the fluorescence observed with the YFP-TSPAN2 constructs. We added exact numeric information of cells that were YFP positive and the numbers of the quantification in the figure legend (page 28).

10) Page 11 the authors stated "Our data support a possible function of phospho-tyrosine 124 mediated TSPAN2 interactions connected to cell migration, adhesion and spreading and thus provides a mechanistic entry point to unravel the effects of elevated TSPAN2 levels in cancer such as lung adenocarinomas". While membrane protrusions have been observed upon TSPAN2 overexpression, the authors do not provide any data that cell migration and adhesion are indeed affected by TSPAN2. Either they tone down their claim or they should provide additional experimental evidence that directly establish a role for TSPAN2 in migration and adhesion.

We toned down our statement and more explicitly refer to the migration and invasion assays that showed the TSPAN2 function reported by Otsubo et al. (Discussion page 15, first paragraph).

11) Would be great if the authors could provide evidence that TSPAN2 phenotypes observed with wt and mutant proteins correlate with their interactions with endogenous Grb2.

The point is well taken, however we provide an interesting lead to demonstrate the usefulness of the data set. Working this out with endogenous proteins is not in the scope of this manuscript.

12) On page 12 the authors conclude that "The more detailed characterization of the TSPAN2-

GRB2 and TSPAN2-PIK3R3 interactions exemplarily demonstrates how PPIs are dynamically and spatially constrained". Whereas evidence is provided (based on PCA overexpression experiments) that these two interactions are spatially separated, there is no evidence provided how these PPI are dynamically constrained in the presented data.

Ok, we have revised this sentence (now page 15 second paragraph).

Minor points

1) The reading of the text could improve by structuring the result section with headings

We included headings in the results section and provide a separate discussion part.

2) A graphical legend within the Figure 2A which explains the nature of edges and nodes should be provided

Ok, a legend was added to Figure 2A.

3) Legend to Figure 3C unclear "Selection of PIK3R3 and GRB2 co-IP results from HEK293 cells (serum conditions)": What do the dark and bright green bars represent? Where are the GRB2 co-IP results?

We apologize for the mistake but the labels (B and C) in the figure 3 were interchanged. This has been corrected, and the legends do match the figures now.

4) The PA-tag should be explained once. I assume it is protein A but it is nowhere explained in the text.

Ok, see improved figure legend to figure 4 and material and methods page 17, first sentence.

5) Page 8 The authors state "In the majority of the cases the C-terminal SH2-domain mutation (R383K) had a stronger diminishing effect on binding, however when both SH2-domains were mutated simultaneously, pY-dependent binding was typically decreased most strongly (Figure 3D)." however Figure 3D does not show the results obtained for R383K of PIK3R3 but only for the double mutant.

We have provided a full figure also showing the single mutations supporting our statement in the main text as Suppl. Figure S8. Additionally, the measured values are given in a new Suppl. Table S9.

6) Please clarify in the method section whether the Co-IP validation experiments were performed in the presence of ABL2 or Fyn or in their absence - this is not clearly stated in the text.

Ok see point 6. Clarified on page 17, material and methods.

7) The authors may add in the last paragraph on page 3 of the introduction that besides linear motifs, complex formation with other human proteins, compartmentalization and additional PTMs may add to the specificity of P-Y recognition.

Yes, done, page 4 end of middle paragraph.

8) Page 7/8: validation of literature data is not clear: what do the authors exactly mean with: 143 literature-curated interactions in the pY-reader - pY-reader search space? Please explain.

We have used ConsensusPathDB to get information about known interactions and checked each original paper. 147 interactions between pY-reader and pY-reader are indeed reliably described (in 117 publications) and used for benchmarking. Please note, that it is often not clear from the data in the papers whether the interaction is phosphorylation dependent or not. However we liberally inferred this and recorded it. The whole analysis is documented in detail in Suppl. Table S7 (literature data) and Suppl. Figure S7 (comparison). Manual curation is important when mining PPIs

© European Molecular Biology Organization

from databases in order to differentiate binary interaction data from protein complex purification data to create a real binary benchmarking set (see e.g. Hegele et al. Mol Cell 2012, Rolland et al. Cell 2014).

9) Fig.S2: Figure as well as legend should contain more information: what kinase construct and controls were used in the retesting? Otherwise the presented data is hard to interpret.

Yes, we and have added a legend that clarifies this point.

Reviewer #3:

In this manuscript the authors describe a new approach to determine human protein-protein interactions that are dependent on protein tyrosine phosphorylation. This was achieved with a modified yeast-two-hybrid (Y2H) protocol using as bait full proteins containing domain families that are known to be able to interact with phosphotyrosines. The dependence on phosphorylation is measured by testing the same interactions in the presence or absence of protein tyrosine kinases. The experimental set-up allowed the authors to determine 292 interactions that appear to depend on the presence of tyrosine kinases and an additional 336 interactions that are independent of the presence of the tyrosine kinases.

As expected for any novel experimental approach the authors have tried to provide extensive testing of the accuracy of the identified interactions. It is this aspect that is somewhat disappointing. To the authors credit they have tried many different approaches to corroborate the phosphorylation dependence of the identified interactions. They have: compared the identified interactions with previous knowledge from the literature; tested the over-representation of tyrosine binding motifs and previously known tyrosine phosphosites; re-tested the same interactions with co-IPs in the presence and absence of expressed kinases as well as with mutated versions of the binding domains. Many of the tests fall short of being overwhelmingly positive. However, the approach is very creative and novel. This is the first large-scale approach to identify in-vivo phosphorylation dependent protein interactions and can, in principle, be extended to other types of PTMs. The most related approach would be conditional dependent affinity-purification mass-spectrometry (AP-MS) but, as was the case for "static" protein interactions, conditional AP-MS and this Y2H approach are expected to provide different and complementary information. The authors have additionally provided more extensive validation for a specific interaction (TSPAN2 with GRB2/PIK3R3) which exemplifies how this resource may be used in the future by researchers interested in cell signalling. Given the novelty of the approach and the potential usefulness of the determined interactions I believe this work would be of interest to large audience.

Thanks for this positive assessment of our work.

Major concerns:

1 - I have only one major concern. As mentioned above, the tests performed to validate the phosphorylation dependence of the interactions are not overwhelmingly positive. For example the overlap with interactions known from the literature is very small (~6%) even if likely to be significant. The interactions were found to be fairly reproducible (50%) in co-IPs done in human cells but almost none of these were found to be dependent on the co-expression of a tyrosine kinase. As pointed out by the authors we might expect that the baseline tyrosine kinase activity of the cells used might make the phosphorylation dependence very hard to measure with these assays. Finally, for a small set of 13 interactions the authors show that impairing the phospho-tyrosine binding domains has a very significant effect on the interactions which indirectly implies that they are phosphorylation dependent. So although the authors have done very extensive computational and experimental tests it remains hard to evaluate to what extent the 292 interactions are indeed dependent on tyrosine phosphorylation. Performing detailed studies of additional interactions is clearly beyond the scope of this article and the authors have already done extensive validation work. It is possible that the only plausible resolution for this concern will be to improve the interpretation and discussion of these results being cautious about the claims.

As the reviewer pointed out, using a novel approach also poses difficulties with validation simply because of the lack of comparable data. We have now added a discussion part and specifically point

out limitations of our approach and set it in relationship to what can be done in human cells. Though in yeast the human proteins are obviously out of context etc. in our hands the pY-Y2H is the most specific approach with respect to assessing phosphorylation dependency, because the system is essentially free of background kinase activity and because we developed a stringent, very well controlled, systematic setup (page 13).

Given that the mutations of the phosphotyrosine binding domains provided the strongest validation the authors may at least try to be clearer about this validation assay. Were the 13 interactions the only ones tested in the way ? This implies a 100% dependence on the phosphotyrosine domain families which is not even fully expected since this was done in the context of full proteins. If this validation assay could be extended to additional domain(s)/interactions it would provide additional assurance of the phospho dependence of the interaction network provided here. However, I would not suggest this is a requirement for publication.

With this point the reviewer is completely correct: What we observe is a reduction in signal in the co-IP assay, and the statistically significant reductions are reported in Figure 3D. We added additional data from this assay: Suppl. Figure S8 shows also the single mutations from PIK3R3 interactions and Suppl. Table 9 the full data of the co-IP assay with all SH2 domain mutations. This also includes examples of pY-dependent interactions from the pY-Y2H where the GRB2-SH2 domain mutation does not diminish binding significantly. As the reviewer stated, this is expected, since GRB2 e.g. has two SH3 domains which support binding via PxxP motifs.

Minor concerns:

1 - How exactly was the kinase dependence scored ? If I understood correctly, all 1223 robust Y2H interactions were re-tested with 5 biological replicates of empty vector and with a panel of tyrosine kinases (as in supplementary figure 3). How did the authors define the kinase dependence and/or kinase specificity ? Was this defined based on a quantitative model from the colony growth/sizes ? There are several interactions (e.g. YES1-OLIG1, PIK3R3-PELO, GRB2-LMX1A, GRB2-PPARA) that are listed as phospho dependent but in supplementary figure 3 the empty vector colonies appear to be growing well. Perhaps the authors could provide a quantitative metric for each interaction describing the kinase dependence and/or kinase specificity of the interaction. For example if all colony growth were quantified using image analysis then the distribution of scores for the empty vectors in each interaction could be used as a the null model to score each of the kinases. Ranking phospho dependence in this way might facilitate the re-use of this dataset.

There is some misunderstanding to what was tested and we hope that the new Figure S2, a flow chart of the screen, will clarify this point. All 1223 hits were subject to retesting, examples of which are given in Suppl. Figure S3. The assay is designed to identify kinase-dependent interactions and is assaying the prey in parallel against 24 bait strains in duplicate each including 12 empty vector controls (please see new figure legend in Suppl. Figure S3). The assay is very stringent and kinase-dependent interactions as well as independent interactions can be identified with high selectivity, so that quantification does not add anything.

The assay the reviewer is referring to in this point is the kinase plate assay (now Suppl. Figure S4). It serves the purpose of testing kinase dead mutations and assays an almost complete set of human non- RTKs (31 of 32) with a selected set of 37 interactions (all shown). The assay is particularly suited to control for the absence of interactions. We use yeast strains that are co-transformed with the interacting bait and prey plasmid and mate this strain against 48 strains carrying the kinases (wild type and kinase death respectively), one interacting pair per plate (for a detailed description of the assay see legend Suppl. Figure S4). This is very laborious but necessary to compare kinase with kinase-dead versions. Put simply, we put much too much yeast on the plates which is why you see some background growth on some of the plates. Please note, that we did not obtain any signal comparable to wild type kinases with the kinase-dead version for any of the tested interactions. Since the strength of a Y2H signal in general depends on many parameters (see discussion, page 13 first paragraph), which do not necessarily reflect the binding event (e.g. affinity), we do not quantify growth of Y2H colonies in the lab.

2 - The kinases that drive the phosphorylation dependence are very often ABL2 (80% of interactions), FYN (53%) or TNK1 (39%). I was not expecting that a single kinase (i.e. ABL2) would

explain so many of the phosphorylation dependent interactions. Do the proportions of phosphorylation dependent interactions for each kinase correspond also to the proportion of known protein-protein interactions that these kinases are known to take part in ? In other words, are kinases that are very often associated with the phosphorylation dependence also have many known protein-protein interactions in human and vice-versa ? If this is not the case, is there another reason for this skewness ? Are the top kinases more promiscuous ? I noticed that ABL2 is described to have a nuclear localization signal. Maybe the putative cellular localizations of the expressed kinases has an impact of their activity in yeast ?

We have added a discussion part that tries to pick up these questions. Kinase specificity may contribute to the interaction specificity we observe in the pY-Y2H approach, but there are other parameters contributing as well. We only identified kinase candidates which can phosphorylate human proteins thereby promoting pY-dependent interactions. Again we tried to address these questions as part of discussion of the limitations of the method (page 13).

3 - There are several assumptions and limitations that could be better discussed. For example, several of the proteins that have phosphotyrosine domains are themselves tyrosine kinases. I assume that these kinases were not mutated before using them as baits. The intrinsic kinase activity of these could make the kinase dependence hard to score. Conversely, many tyrosine kinases exist in an inactive state. The authors essentially look for positive signals but it is possible that several of the over-expressed kinases might not have enough basal activity to phosphorylate the target proteins. On the other hand it is probably good that the the authors did not use activated kinases since it is known that addition of active tyrosine kinases to yeast cause fitness defects. I don't think the authors mentioned that S. cerevisiae does not have tyrosine kinases which is an obvious advantage to performing these studies in yeast. I think several of these aspects should be better explained and discussed in the manuscript.

These points are well taken and we have included them in the discussion (page 13). As indicated in this comment by the reviewer already, kinases are expressed at very low levels not only to avoid fitness defects, but also to gain specificity. Therefore, some kinases may not be active enough under the conditions used, but this goes on the account of false negative signals.

Using kinases that contain SH2 domains as bait we indeed observe both phospho-dependent and independent interactions. We cannot rule out that some of the independent are rather phospho-dependent but catalyzed by the bait (see discussion on page 13 middle paragraph).

4 - Several figures could benefit from additional labels. In most cases where color is used to convey information the labels are not available in the figures and one has to read the figure legends to decode it. Examples include Figure 2A, 2B, 2C and 3C.

We improved the figures and added legends for the color information.

2nd Editorial Decision 17 February 2015

Thank you again for submitting your work to Molecular Systems Biology. We have now heard back from the referee who was asked to evaluate your manuscript. As you will see below, this reviewer (#2) is satisfied with the modifications made and thinks that the study is now suitable for publication.

Reviewer #2:
The authors greatly improved their manuscript in the revised version and have addressed most points I have raised. I am convinced that this work will raise significant interest in cell signalling and thus strongly support publication of their work in MSB.
